# DNA methylation profiling reveals novel diagnostic biomarkers in renal cell carcinoma

**DOI:** 10.1186/s12916-014-0235-x

**Published:** 2014-12-04

**Authors:** Brittany N Lasseigne, Todd C Burwell, Mohini A Patil, Devin M Absher, James D Brooks, Richard M Myers

**Affiliations:** HudsonAlpha Institute for Biotechnology, 601 Genome Way, Huntsville, AL 35806 USA; Department of Biological Sciences, University of Alabama in Huntsville, Shelby Center for Science and Technology, Room 369, 301 Sparkman Drive, Huntsville, Alabama 35899 USA; Department of Urology, Stanford University, 875 Blake Wilbur Dr. Clinic E, Stanford, California 94305-5118 USA

**Keywords:** Cancer, Diagnostic biomarker, DNA methylation, Kidney, Renal cell carcinoma

## Abstract

**Background:**

Renal cell carcinoma (RCC) is the tenth most commonly diagnosed cancer in the United States. While it is usually lethal when metastatic, RCC is successfully treated with surgery when tumors are confined to the kidney and have low tumor volume. Because most early stage renal tumors do not result in symptoms, there is a strong need for biomarkers that can be used to detect the presence of the cancer as well as to monitor patients during and after therapy.

**Methods:**

We examined genome-wide DNA methylation alterations in renal cell carcinomas of diverse histologies and benign adjacent kidney tissues from 96 patients.

**Results:**

We observed widespread methylation differences between tumors and benign adjacent tissues, particularly in immune-, G-protein coupled receptor-, and metabolism-related genes. Additionally, we identified a single panel of DNA methylation biomarkers that reliably distinguishes tumor from benign adjacent tissue in all of the most common kidney cancer histologic subtypes, and a second panel does the same specifically for clear cell renal cell carcinoma tumors. This set of biomarkers were validated independently with excellent performance characteristics in more than 1,000 tissues in The Cancer Genome Atlas clear cell, papillary, and chromophobe renal cell carcinoma datasets.

**Conclusions:**

These DNA methylation profiles provide insights into the etiology of renal cell carcinoma and, most importantly, demonstrate clinically applicable biomarkers for use in early detection of kidney cancer.

**Electronic supplementary material:**

The online version of this article (doi:10.1186/s12916-014-0235-x) contains supplementary material, which is available to authorized users.

## Background

In 2013, approximately 65,000 cases of renal cell carcinoma (RCC) were diagnosed in the United States and 13,600 patients died of the disease. RCC incidence is rising by approximately 2 to 3% per year [1-4], in large part due to the increasing use of abdominal imaging [5]. Nearly half of all renal tumors are discovered incidentally [5,6], 20% of small tumors (less than 4 cm) are benign [7], and there are no imaging features or biomarkers that reliably distinguish benign from malignant disease [8]. For cancers confined to the kidney, the standard of care is resection, with high 5-year survival rates. Survival rates are directly correlated with tumor stage and size, demonstrating the importance of early detection of lesions when the lesions are small. Following tumor resection, patients must be monitored for recurrence at regular intervals by imaging studies (usually CT scanning), and thus incur significant radiation exposure with the attendant risks [9,10]. Once metastatic, RCC is usually fatal, despite treatment with targeted therapies, although a small fraction of patients show durable responses to IL-2 immunotherapy [3,11,12].

RCC is classified into histological subtypes with distinct clinical and pathogenic features [13]. Clear cell renal cell carcinoma (ccRCC), the most clinically aggressive subtype, comprises 75% of cases and is characterized by inactivation of the von Hippel-Lindau (*VHL*) tumor suppressor gene, a regulator of oxygen sensing in the cell by controlling HIF1α protein levels [14]. Papillary RCC (pRCC; 10% of cases) commonly has trisomy of chromosomes 7 and 17 and may be less clinically aggressive than ccRCC. Chromophobe carcinomas (chRCC) are the least aggressive tumors and comprise 5% of cases. Additionally, less common RCC subtypes arise from various cells of the nephron and present diverse clinical behavior [15]. Perhaps because of the histologic, molecular, genetic, and clinical diversity of RCC and its origin from different cell types in the nephron, biomarkers for use across the most common histologic subtypes types of RCC for detection or monitoring have not been previously reported.

Detection of DNA methylation at candidate loci in RCC suggests that tumor-specific methylation changes could be used diagnostically. However, the performance characteristics of these markers have limited their utility. Small studies have shown that simultaneous measurement of several differentially methylated loci could improve performance [16-18]. We hypothesized that better biomarkers could be identified by using genome-wide studies of DNA methylation in RCC. Such previous studies indicated that DNA methylation changes are early events in carcinogenesis, but these experiments were not designed to identify cancer-specific diagnostic biomarkers [19-24]. In this study, we profiled DNA methylation in tumor and adjacent normal tissue in 96 RCC patients with Illumina HumanMethylation27 microarrays. Using the predictive analysis of microarray (PAM) classification tool [25], we identified a biomarker panel capable of differentiating kidney tumors from benign adjacent kidney tissue, irrespective of tumor histology. This study provides insight into RCC etiology, presents validated tissue-based diagnostic biomarkers, and supplies a framework for the development of DNA methylation-based molecular diagnostics for RCC detection in patients.

## Methods

### Sample collection and preparation

Kidney samples used in this study were collected at Stanford University in accordance with approved institutional review board protocol (6208, Panel: 8) with patients’ informed consent under protocols approved by the Stanford University and HudsonAlpha Institute for Biotechnology Institutional Review Board. Signed patient consent for use of kidney tissue states that clinical and pathological data can be associated with their clinical samples and tissue would otherwise be discarded after processing for clinical care. Consent forms are stored at Stanford University and available for review according to local, state, and federal regulations. Immediately after surgical removal of the kidney, fresh normal and tumor tissue samples were harvested, flash frozen in liquid nitrogen, and stored at −80°C until they were used. For each sample, a frozen section was taken, stained with hematoxylin and eosin, and evaluated by a genitourinary pathologist. Normal samples were harvested distant from the tumor and confirmed by histology to have no contamination with malignant cells. Cancer samples were also confirmed to be enriched (>80% epithelial cells) for cancer cells relative to stroma. Cancer samples were macroscopically dissected to remove normal contamination and necrotic tissue using the hematoxylin and eosin-stained sections as a guide.

Clinical information associated with each patient is summarized in Additional file 1: Table S1. We isolated DNA from fresh-frozen tissue samples using the QIAGEN AllPrep DNA kit (QIAGEN) following the manufacturer’s protocol.

### Sodium bisulfite conversion and Illumina Infinium HumanMethylation27 assay

We performed sodium bisulfite conversion of gDNA using the EZ-96 DNA Methylation Kit (Deep-well format, ZymoResearch) with the alternative incubation protocol for the Illumina Infinium Methylation Assay, as described by the manufacturer. We assayed 500 ng of sodium bisulfite-converted gDNA from patient tissues by Infinium HumanMethylation27 RevB Beadchip Kits (Illumina) per the manufacturer’s protocol.

### Beta score calculations, filtering, and batch normalization of methylation data

We analyzed HumanMethylation27 array results using Illumina BeadStudio software with the Methylation Module v3.2. Any negative beta scores were converted to a zero and any beta scores with an associated detection *P* value of >0.01 were converted to “NA” and filtered from analysis. To correct any array-by-array variation, we imputed all missing values with KNN Impute, followed by array batch normalization using the ComBat R-package [26]. Previously imputed values were converted back to “NA” for all further analyses. CpGs with “NA” in more than 10% of samples were removed from the data set. As previously reported, we removed CpGs with questionable mapping or those including a SNP of >3% minor allele frequency within 15 bp of the assayed CpG to avoid potential variation in probe hybridization [27]. After quality control and filtering, we had 96 patients with 26,148 CpGs assayed in both kidney tumor and benign adjacent tissues.

### Linear mixed and logistic regression analysis

For the regression analysis we used RStudio (version 0.97.551) in R (version 3.0.0). For the linear mixed model analysis of the methylation data we used the lme command treating patients as a random effect and age and gender as fixed effects. We used the glm command with family set to binomial for the logistic regression of the diagnostic biomarkers. We selected our best model based on a maximum receiver operating characteristic (ROC) curve area and a minimum Akaike Information Criterion (AIC) value. All regression models have *P* values adjusted for multiple hypothesis testing (false discovery rate, FDR) using the Benjamini and Hochberg (BH) algorithm and significant CpGs have an adjusted *P* <0.05.

### Hierarchical clustering

Prior to hierarchical clustering, we mean-centered beta scores. We performed hierarchical clustering of the methylation data by both gene and array using Cluster 3.0 with average linkage [28].

### Prediction analysis of microarrays (PAM)

We performed PamR (version 1.54) analysis on all filtered CpGs as described in the PamR manual with RStudio (version 0.97.551) in R (version 3.0.0) [25]. Based on visual examination of the training errors and cross-validation results, we minimized the miss-rate and set the shrinkage threshold to 10.74 for all tumor and benign adjacent normal classification, and 14.8 for clear cell tumor and benign adjacent normal classification.

### Gene ontology (GO)-term and gene set enrichment analysis (GSEA)

We associated CpGs identified as significant with the closest gene and then those genes were analyzed for common pathways and functions. Terms reported have an adjusted (FDR) *P* <0.05. We performed GO-term analysis using the web version of GOrilla [29] and we performed GSEA using the web version of GSEA [30,31] with KEGG, BIOCARTA, and REACTOME gene sets selected.

### The Cancer Genome Atlas (TCGA) data

We downloaded TCGA Illumina HumanMethylation27 and HumanMethylation450 Level 3 array results for all kidney cancer patients available at the time of manuscript preparation. Diagnostic biomarker validation for ccRCC patients utilized HumanMethylation27 tumor and matched benign adjacent normal ccRCC TCGA data only. Diagnostic biomarker validation for the general RCC patients utilized both HumanMethylation27 and HumanMethylation450 tumor and matched benign adjacent normal ccRCC, pRCC, and ChRCC TCGA data. We downloaded RNA expression data for ccRCC patients using the RNA-seq Level 3 data available at the time of manuscript preparation.

## Results

### Identification of differential methylation between kidney tumor tissue and benign adjacent kidney tissue

We collected clinical data, including histologic subtype, tumor grade and stage, and clinical follow-up, for 96 patients (Additional file 1: Table S1). We profiled DNA methylation of both kidney tumor and adjacent benign normal tissue from each patient by using Illumina HumanMethylation27 arrays, which interrogate 27,578 CpGs located primarily in the promoter regions of genes in the human genome. After quality control and filtering, we performed DNA methylation analysis on results from 96 patients and 26,148 CpGs. Methylation at 127 CpGs was validated in 19 of the benign adjacent kidney tissues with Bis-seq, as reported previously [32]; concordance was 0.82. Additionally, our group has previously validated this same platform with PyroMark in prostate cancer [27]. To identify CpGs carrying tumor-specific aberrant methylation, we performed linear mixed modeling with paired tumor/normal data at each CpG. Our models treated patient ID as a random effect and included gender and age as fixed effects. When we analyzed all CpGs and patients with these models, 9,800 CpGs were significantly different between kidney tumor and benign adjacent kidney tissue (FDR <0.05). Of these, 5,155 CpGs had increased methylation and 4,645 had decreased methylation in tumors compared to benign adjacent tissue (Additional file 1: Table S2).

**Figure 1:**
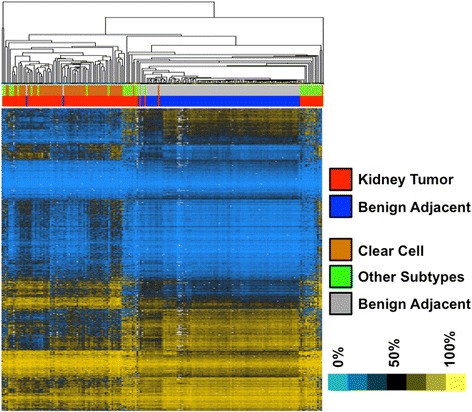
**Hierarchical clustering of kidney tumor and benign adjacent tissues with most significant DNA methylation changes.** Hierarchical clustering by both sample and CpG of 192 kidney tumor (red color bar) and kidney benign adjacent (blue color bar) tissues with linear mixed model significant CpGs (FDR <1 × 10^-10^; 1,172 CpGs); (blue pixels) low DNA methylation; (yellow pixels) high DNA methylation; (orange color bar) ccRCC tissues; (green color bar) other subtype RCC tissues; (grey color bar) benign adjacent tissues.

Using the most significant CpGs from the linear mixed effects model (1,172 CpGs, FDR <1 X 10^-10^), we performed hierarchical clustering by sample and CpG (Figure 1). We observed one cluster with shorter branch lengths that contained all but three of the normal tissue samples (Figure 1). Most of the tumors were in the surrounding clusters and off-shoots with longer branch lengths, indicating greater heterogeneity in the tumor methylation profiles [33].

We performed a GSEA of the GO-terms and pathways associated with genes nearest CpGs exhibiting increased and decreased methylation in tumors compared to benign adjacent normal tissues (Additional file 1: Table S3) [29-31] to assist interpretation of significant findings. In the tumors, decreased methylation showed enrichment in genes associated with immune function and G-protein coupled receptor (GPCR) signaling. Previous reports have indicated upregulation of immune-related genes and suggested some of that deregulation might be explained by epigenetic changes [34]. Terms associated with increased methylation included cell-cell signaling and gated channel activity. Genes associated with GPCR signaling also showed increased methylation. While gene expression changes in GPCR signaling have been previously reported [35,36], to our knowledge, this is the first report of widespread differential methylation in genes related to GPCR signaling in RCC. Additionally, pathways associated with integration of energy metabolism, extracellular matrix organization, and WNT signaling were significantly enriched.

We repeated the regression analysis including only the 63 patients with ccRCC in our patient collection and found that about 85% of the differentially methylated CpGs continued to be significant (FDR <0.05) (Additional file 1: Table S2). These included CpG loci near *VHL*, *SETD2*, *BAP1*, and *UQCRH*, all genes previously shown to be mutated in RCC patients [37,38]. However, we did not see significant DNA methylation changes for *PBRM1*, a gene previously shown to have truncating mutations in RCC at a level superseded only by *VHL* [38]. Likewise, a gene set enrichment and GO-term analysis of the significantly altered CpGs in the ccRCC specimens revealed functions and pathways very similar to the results we obtained when analyzing all 96 of the tumors in our study set (Additional file 1: Table S3).

### Diagnostic methylation markers

**Figure 2:**
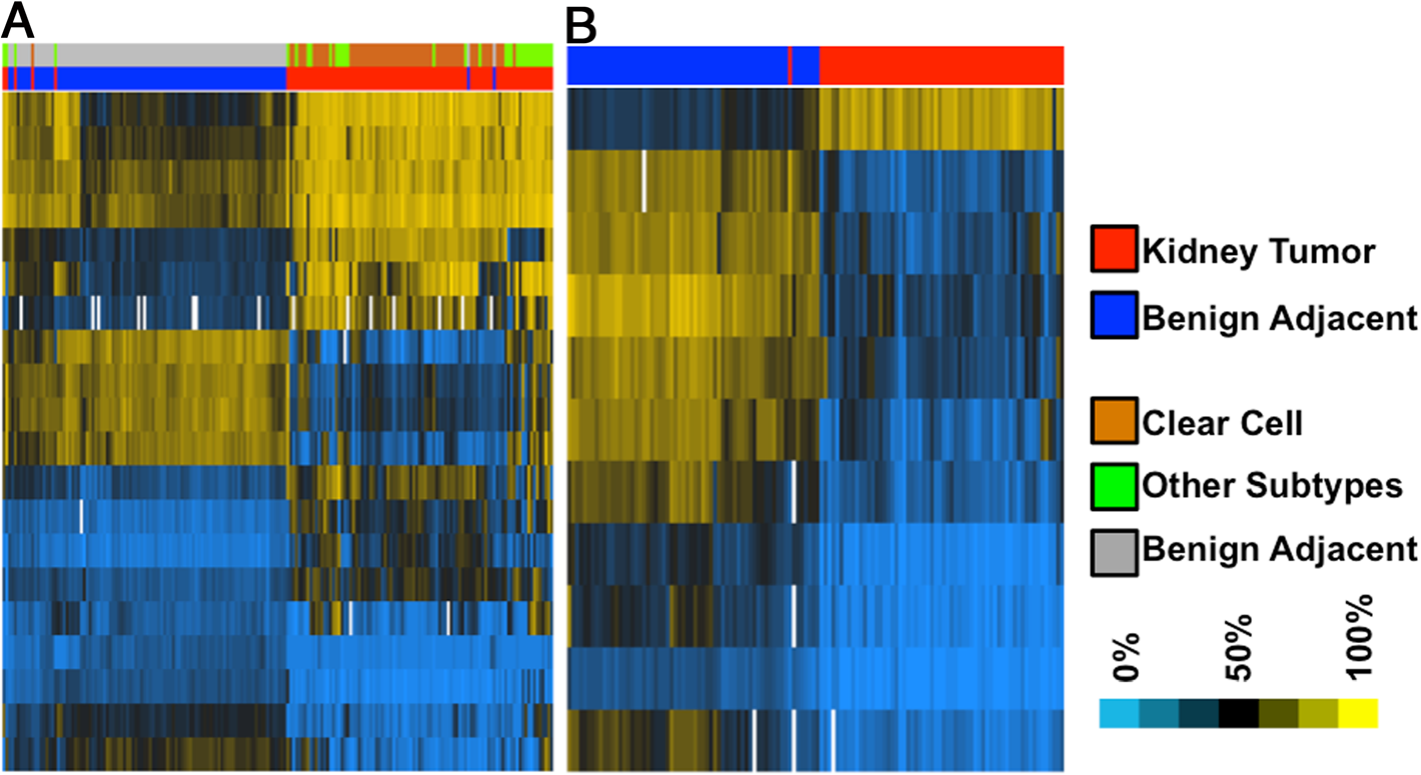
**Hierarchical clustering of kidney tumor and kidney benign adjacent tissues with PAM classifier panel CpGs. (A)** Hierarchical clustering by both sample and CpG of all 192 kidney tumor and kidney benign adjacent tissues with PAM classifier panel CpGs (20 CpGs). **(B)** Hierarchical clustering by both sample and CpG of 126 clear cell kidney tumor and kidney benign adjacent tissues with PAM classifier panel CpGs (11 CpGs); (blue pixels) low DNA methylation; (yellow pixels) high DNA methylation; (red color bar) tumor tissues; (orange color bar) ccRCC tissues; (green color bar) other subtype RCC tissues; (blue/grey color bar) benign adjacent tissues.

We used PAM to identify a set of markers that best distinguished normal from malignant kidney samples [25,27]. PAM uses a shrunken centroid classification algorithm to identify significant CpGs whose methylation distinguishes tumor tissue from benign adjacent tissue. When all 192 tissues were included in the analysis, we identified 20 CpGs that discriminated between benign adjacent tissue and tumor tissue (Additional file 1: Table S4). Using this list of 20 CpGs, we performed hierarchical clustering on both the samples and CpGs (Figure 2, panel A). Visual inspection showed that 91 of 96 tumors cluster together and 93 of 96 benign adjacent normal tissues cluster together. Nineteen of these 20 CpGs were significant in our linear mixed model analysis of CpGs that are differentially methylated between tumor and benign adjacent kidney tissue (FDR <0.05).

When we repeated this analysis with only the 63 ccRCC patient specimens, PAM identified 11 CpGs that discriminated between benign adjacent and tumor tissues (Additional file 1: Table S4). Hierarchical clustering on both the samples and the CpGs (Figure 2, panel B) showed almost perfect classification with only one tumor sample clustering with the benign adjacent normal tissues. These 11 CpGs were also significant in our linear mixed model analysis of the ccRCC tumor data and the benign adjacent normal data (FDR <0.05). The better classification of the ccRCCs compared to all of the subtypes was most likely because of the greater heterogeneity in the DNA methylation profiles between all of the subtypes. There were 4 CpGs that overlapped between the ccRCC and all tumor CpG loci identified by PAM.

Using our data as a training set, we built a logistic regression model from the PAM diagnostic list capable of discriminating between tumor and benign adjacent tissues. From the 20 CpG panel for the multiple subtype PAM list, we selected a model with the greatest ROC area under the curve (AUC) and an AIC at a local minima compared to models with 1 to 20 CpGs. This 5 CpG model (comprised of cg13156411, cg14456683, cg18003231, cg12782180, and cg22719623) had a ROC area of 0.991 and a BH-adjusted highly significant *P* value of 8.10 × 10^-31^ for the null hypothesis that the ROC curve area is 0.5 (which would indicate that a model would not discriminate between tumor and benign adjacent tissue).

**Figure 3:**
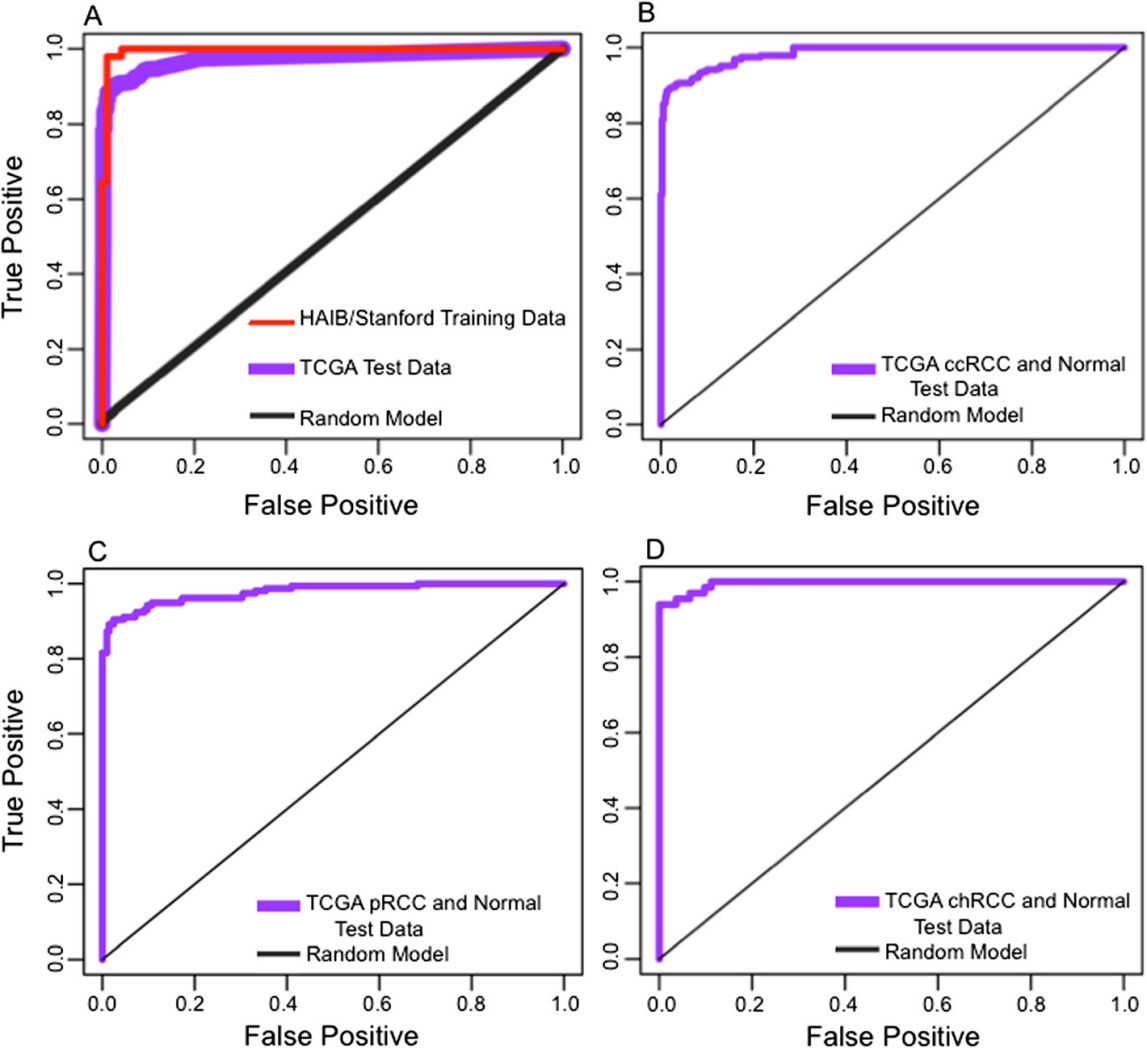
**PAM diagnostic panel model for renal cell carcinoma. (A)** ROC curve of best 5 CpG model (Benjamini and Hochberg-adjusted P = 8.10 × 10^-31^) from PAM diagnostic panel produced via the HudsonAlpha/Stanford data (ROC AUC = 0.991), and applied to the TCGA data (ROC AUC = 0.990). **(B)** ROC curve of best 5 CpG model applied to TCGA ccRCC and normal kidney tissue data (ROC AUC = 0.98). **(C)** ROC curve of best 5 CpG model applied to TCGA pRCC and normal kidney tissue data (ROC AUC = 0.97). **(D)** ROC curve of best 5 CpG model applied to TCGA chRCC and normal kidney tissue data (ROC AUC = 0.99). Random model is 50 random draws of 5 non-significant training set CpGs.

We used publicly available renal cell carcinoma genomic data from TCGA as a validation test set for our predictive model. TCGA has DNA methylation data available for 732 RCC (ccRCC, papillary, and chromophobe) tissues and 410 normal kidney tissues. When we applied our 5 CpG model to all of the TCGA samples (Additional file 2: Figure S1), the ROC AUC was 0.990 and we correctly predict 87.8% of the normal and 96.2% of the tumor tissues (Figure 3, panel A). When this model was applied to each histologic subtype of kidney cancer and the normal tissues (n = 410), the ROC AUC remained outstanding in all tumor types. For ccRCC (n = 509), the AUC was 0.98, and we correctly predicted 96.1% of the tumor samples (Figure 3, panel B). For pRCC (n = 157), the ROC AUC was 0.97 and we correctly predicted 94.9% of the tumor samples (Figure 3, panel C). The ROC AUC for chRCC (n = 66) was 0.99 and we correctly predicted 100% of the tumor tissues (Figure 3, panel D). Model sensitivity was largely independent of cancer stage and primary tumor size (Additional file 1: Table S5).

**Figure 4:**
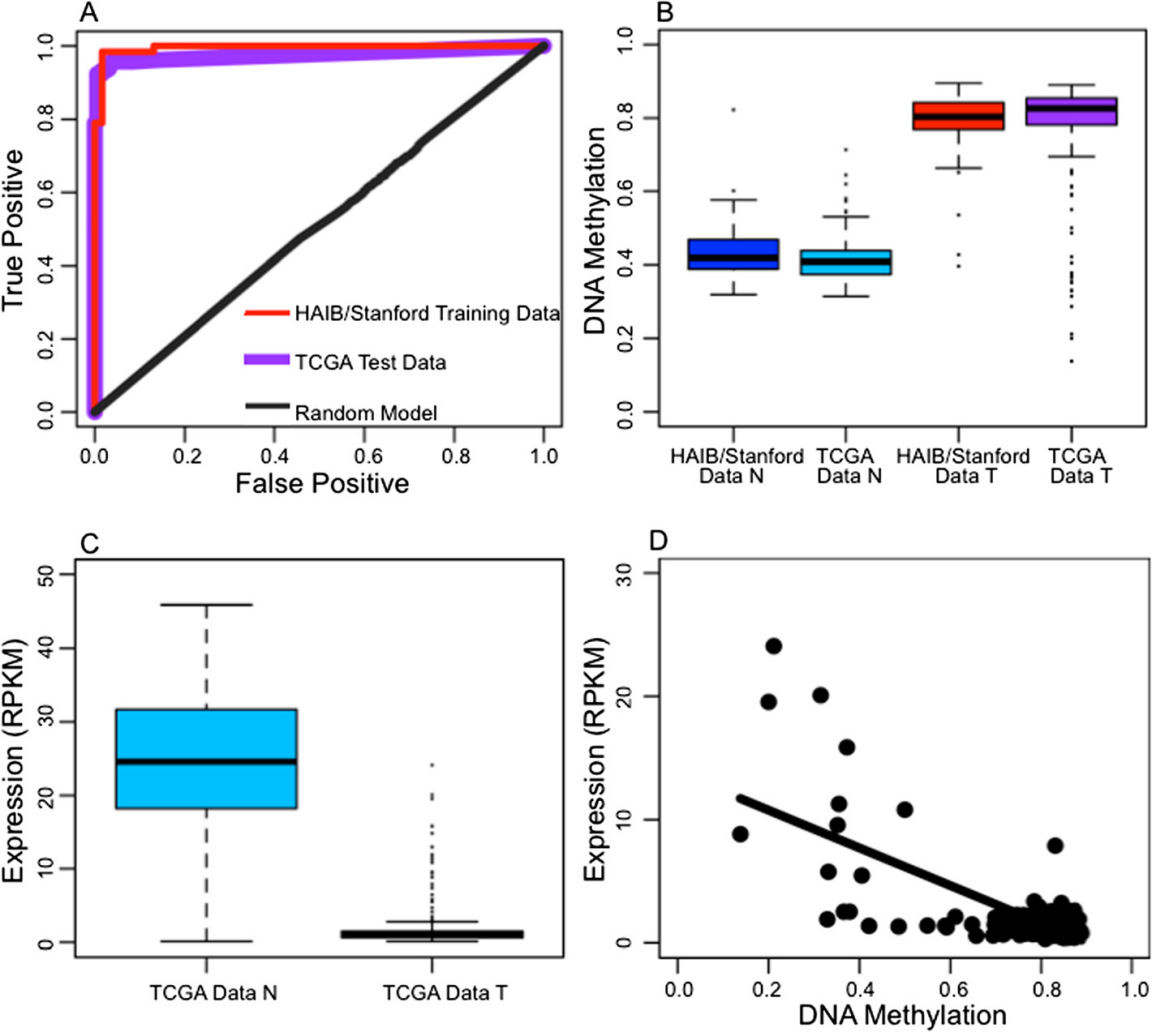
**PAM diagnostic panel model for clear cell renal cell carcinoma. (A)** ROC curve of best 4 CpG model (Benjamini and Hochberg-adjusted P = 1.46 × 10^-20^) from PAM diagnostic panel produced in the HudsonAlpha/Stanford data (ROC AUC = 0.990) and applied to TCGA (ROC AUC = 0.972). **(B)** DNA methylation at cg04511534, a CpG in the most predictive HudsonAlpha/Stanford model (Mann-Whitney test; Bonferroni-adjusted P = 0.2524 for HudsonAlpha/Stanford normal tissues versus TCGA normal tissues; Bonferroni-adjusted *P* = 0.1848 for HudsonAlpha/Stanford tumor tissues versus TCGA tumor tissues; Bonferroni-adjusted P <0.0001 for HudsonAlpha/Stanford normal tissues versus TCGA tumor tissues, Bonferroni-adjusted P <0.0001 for HudsonAlpha/Stanford tumor tissues versus TCGA normal tissues). **(C)** Expression of *GGT6* in TCGA tumor and normal tissue data (Mann-Whitney test; P <0.0001). **(D)** *GGT6* expression versus cg04511534 methylation in TCGA tumor tissue data (linear regression; P <0.0001, R^2^ = 0.5030). Random model is 50 random draws of 5 non-significant training set CpGs.

When the ccRCC patients were analyzed separately, 4 CpGs (cg04511534, cg11098259, cg14391855, and cg26366091) produced a ROC AUC of 0.990 with a BH-adjusted *P* = 1.46 × 10^-20^ for the null hypothesis that the ROC AUC is 0.5. In TCGA specimens (208 tumor tissues and 200 normal tissues), the 4 CpG model showed an AUC of 0.972 (Figure 4, Panel A) and we correctly identified 91.4% of the tumors and 98.9% of the benign adjacent tissues. Model sensitivity remained high regardless of cancer stage and primary tumor size (Additional file 1: Table S6). A comparison of the DNA methylation differences between tumor and benign adjacent normal tissues demonstrated good agreement between our and the TCGA datasets. For example, at cg04511534, we saw no statistical difference between our data and TCGA data (*P* = 0.25 and 0.18); however, the difference between tumor and normal was consistent between the two data sets (*P* <0.0001) (Figure 4, Panel B) (Additional file 2: Figure S1).

RNA-seq expression data was available for a subset of ccRCC patients in the TCGA data, allowing us to investigate whether the changes in DNA methylation we observed are correlated with transcript levels at nearby genes. In 9 of 11 genes, significantly different levels of gene expression were observed in the gene closest to the significant CpG (Mann-Whitney test, *P* <0.05) in the expected direction based on methylation status. For example, cg04511534, located in the first intron of the gene encoding gamma-glutamyltransferase 6 (*GGT6*), is hypermethylated in tumors compared to normal tissues. Transcript levels of *GGT6* were significantly decreased in the tumor samples compared to the normal tissues (Mann-Whitney, *P* <0.0001; Figure 4, Panel C) (Additional file 3: Figure S2). The relationship between cg04511534 DNA methylation and *GGT6* expression shows significant correlation, following the expected canonical model of increased DNA methylation in the tumors leading to decreased expression (linear regression, *P* <0.0001; R^2^ = 0.503; Figure 4, Panel D). For the other 10 CpGs from the diagnostic panel and the genes to which they are near, we found a range of *P* values (0.0001 to 0.8540) and R^2^ values (0.0001671 to 0.503), with significant correlation between RNA expression and DNA methylation in 8 of 11 pairs.

## Discussion

We found large scale, genome-wide changes in 5-methylcytosine encompassing 9,800 CpGs that differentiated malignant from normal kidney tissues. Furthermore, we developed a panel of 20 CpGs that can discriminate ccRCC, pRCC, and chRCC from normal renal tissue, as well as a panel of 11 CpGs that discriminate between ccRCC tumor and normal tissues. These methylation differences were validated independently in TCGA data and retained high sensitivity and specificity for distinguishing malignant from adjacent normal tissue for all three histologic subtypes. Because of the high sensitivity and specificity of our biomarkers in both data sets, these panels are strong candidates for development of a clinical test for detection of all three major histologic types of RCC.

The candidate biomarkers we identified were all located in the promoter regions or first introns of nearby genes, many of which are interesting in RCC. For example, our most predictive CpG is in the first intron of gamma-glutamyltransferase 6 (*GGT6*)*. GGT6* participates in leukotriene synthesis, glutathione metabolism, and gamma-glutamyl transfer. *GGT6* has been linked to immune infiltration and inflammation and putatively to alterations in DNA methylation via changing levels of glutathione and methyl group availability [39,40]. Other CpGs are associated with genes related to immune function, including T cell regulation and inflammation, tumor initiation and angiogenesis (*EBI3*) [41,42], while other genes have been implicated in cancer aggressiveness (*AQP9*, *RIN1*) [43-47].

Some panel CpGs are in the promoters of genes previously implicated as biomarkers, or involved in carcinogenesis, cancer progression, or treatment response. For example, *PENK* promoter methylation was demonstrated in prostate cancer, breast cancer brain metastasis, and pulmonary adenocarcinoma, and as a potential methylation biomarker for colorectal, meningioma, and bladder cancer [48-53]. *KLK10* promoter methylation has been implicated in head and neck squamous cell carcinoma progression, as well as a potential prostate cancer and non-small cell lung cancer biomarker [54-56]. *ZIC1* was implicated as a tumor suppressor silenced via promoter methylation in malignant pleural mesothelioma and gastric cancer [57,58]. *ZIC1* promoter methylation has also been suggested as a potential prognostic marker in ovarian cancer [59]. Additionally, *C21ORF123* promoter methylation has been implicated in cisplatin resistance in non-small cell lung cancer and *RIN1* is a tumor suppressor that is deregulated through aberrant promoter methylation in breast cancer [60,61]. Interestingly, one of the CpGs showing decreased DNA methylation in tumors is in the promoter of serum amyloid A1 (*SAA1*), which has been previously implicated in RCC. *SAA1* is highly expressed during inflammation and has been identified as a potential Wilms’ tumor marker [62], as one of a two-protein signature model for prognosis prediction in metastatic RCC [63], and as a predictor in fatal outcome in RCC [64].

While it is possible that additional genomic biomarkers could be identified with higher resolution methylation assays, the exceptional performance of these biomarkers in training and test sets, as well as their ability to identify RCC tumors of diverse histology, make them outstanding candidates for development of clinical grade assays. As an increasing number of small renal lesions are being detected through cross-sectional imaging, targeted biopsies of lesions are used to determine whether these lesions are malignant or benign. An assay based on detection of methylated sequences could find immediate application in these diagnostic renal biopsies. However, in a significant fraction of biopsies, insufficient tissue is obtained for histologic diagnosis of malignancy and a genomic assay incorporating our methylation loci could be useful for detecting malignant cells in small biopsies. Furthermore, detection of these methylated sequences could be deployed as a non-invasive detection test in patient blood or urine. Measurement of differential genomic marks in patient blood and urine has already proven useful for diagnosis of cancer in the clinical setting. For example, differential methylation of *Sept9* in patient blood is used for diagnosis of colorectal cancer [65,66] and detection of the non-coding RNA PCA3 in patient urine is used for risk assessment of prostate cancer [67-70]. In RCC of all types, a blood or urine assay could be used for early detection in high risk populations, for monitoring patients after definitive surgical treatment, or possibly in monitoring response to therapy in patients with advanced disease.

Additional work will be necessary to evaluate the performance of these methylation markers in less common RCC histologies, as well as benign entities such as angiomyolipoma or hemorrhagic cysts. Further investigation of the consequences of DNA methylation at these predictive loci, including assessments of gene expression and other DNA alterations, could provide insights into important biological processes common to ccRCC, pRCC, and chRCC.

## Conclusions

RCC survival rates are directly correlated with tumor stage and size and, once metastatic, RCC is usually fatal, demonstrating the importance of early detection when lesions are small. However, RCC tumors are difficult to diagnose because of non-specific symptoms and a reliance on imaging technologies. Due to the molecular and clinical diversity of RCC, biomarkers for use across the most common histologic subtypes of RCC for detection or monitoring have not been previously reported. We have discovered and validated a DNA methylation biomarker panel that is capable of differentiating kidney tumor from benign adjacent kidney tissue, irrespective of tumor histology and with high sensitivity and specificity across all tumor stages. These biomarkers could potentially aide in early clinical detection of kidney cancer, distinguishing between benign and malignant lesions, and monitoring patients after therapy.

## Additional files

Additional file 1: Table S1. Clinical information associated with kidney patients. For each patient, Tumor ID# (unique identification ID for tumor tissue sample), Normal ID# (unique identification ID for benign adjacent normal tissue sample), Age (patient age at time of resection), Gender, Surg type (surgical type for tissue resection), Tumor type, Grade, Stage, Margins, Tumor size (in centimeters), Recurrence status, Status code, Time FU event (time to follow-up event after surgery, in months), and Time death (time to death after surgery, in months). **Table S2.** Significant linear mixed model analysis results. For each CpG significant in a linear mixed model analysis of the methylation data treating patient as a random effect and age and gender as fixed effects (FDR <0.05), the CpG and which model it was significant in are reported. **Table S3.** Significant GO and GSEA terms (adjusted *P* <0.05) for genes identified in linear mixed model analysis. For each significant GO and GSEA term identified from the linear mixed model analysis, the term identifier, term description, the model the term was significant for, and whether the term was significant for genes associated with CpGs with increasing or decreasing methylation in tumors compared to benign adjacent normal is reported. **Table S4.** Significant CpGs by PAM analysis. For each CpG significant in the PAM analysis, the cancer type the CpG was significant for, and the associated gene is reported. **Table S5.** Model sensitivity by cancer stage for five CpG multiple cancer models. For each cancer subtype, model sensitivity was calculated by stage for TCGA patients. Asterisks (*) indicate the inclusion of patients in the total without a specified cancer stage: ccRCC (n = 11), pRCC (n = 38), chRCC (n = 2), and total (n = 51). Model sensitivity in tumors smaller than 4 cm (T1a) was also calculated for each subtype: ccRCC (0.92, n = 125), pRCC (0.92, n = 37), chRCC (1.00, n = 5), and total (0.92, n = 167). **Table S6.** Model sensitivity by cancer stage for four CpG ccRCC model. Model sensitivity was calculated by stage for TCGA patients. Model sensitivity in tumors smaller than 4 cm (T1a) was also calculated (0.86, n = 50). Additional file 2: Figure S1. Remaining diagnostic panel biomarker CpG values from models. DNA methylation of the five CpGs in both HudsonAlpha/Stanford patients and TCGA patients in the best RCC diagnostic model (cg13156411, cg14456683, cg18003231, cg12782180, and cg12782180; panel A–E), and the other three CpGs in both HudsonAlpha/Stanford patient and TCGA patients in the best ccRCC diagnostic model (cg11098259, cg14391855, and cg26366091; panel F–H). In all panels, HudsonAlpha/Stanford Data N versus HudsonAlpha/Stanford Data T and TCGA Data N versus TCGA Data T comparisons are significant (Mann-Whitney test; Bonferroni-adjusted *P* <0.0001). Additional file 3: Figure S2. RNA expression values for genes nearest other three predictive CpGs in ccRCC. RNA expression of the three other genes in TCGA patients in the best ccRCC diagnostic model (panel A–C, Mann-Whitney test; *P* <0.0001). The CpGs are all in the promoter regions of the respective genes (cg11098259 and AQP9; cg14391855 and RIN1; cg26366091 and CHI3L2; panel A–C).

## Abbreviations

AIC: Akaike information criterion
AUC: Area Under Curve
BH: Benjamini-Hochberg
ccRCC: Clear cell renal cell carcinoma
chRCC: Chromophobe renal cell carcinoma
FDR: False discovery rate
GGT6: Gamma-glutamyltransferase 6
GO: Gene ontology
GPCR: G-protein coupled receptor
GSEA: Gene set enrichment analysis
PAM: Probability Analysis of Microarrays
pRCC: Papillary renal cell carcinoma
RCC: Renal cell carcinoma
ROC: Receiver operating characteristic
TCGA: The Cancer Genome Atlas
VHL: von Hippel-Lindau
