# Supplementary material for: DNA methylation profiling reveals novel diagnostic biomarkers in renal cell carcinoma
Source: BMC Med. 2014 Dec 4;12:235. doi: 10.1186/s12916-014-0235-x (PMC4265327; doi:10.1186/s12916-014-0235-x)
Supplement: Additional file 1: Table S1. — Clinical information associated with kidney patients. For each patient, Tumor ID# (unique identification ID for tumor tissue sample), Normal ID# (unique identification ID for benign adjacent normal tissue sample), Age (patient age at time of resection), Gender, Surg type (surgical type for tissue resection), Tumor type, Grade, Stage, Margins, Tumor size (in centimeters), Recurrence status, Status code, Time FU event (time to follow-up event after surgery, in months), and Time death (time to death after surgery, in months). Table S2. Significant linear mixed model analysis results. For each CpG significant in a linear mixed model analysis of the methylation data treating patient as a random effect and age and gender as fixed effects (FDR <0.05), the CpG and which model it was significant in are reported. Table S3. Significant GO and GSEA terms (adjusted P <0.05) for genes identified in linear mixed model analysis. For each significant GO and GSEA term identified from the linear mixed model analysis, the term identifier, term description, the model the term was significant for, and whether the term was significant for genes associated with CpGs with increasing or decreasing methylation in tumors compared to benign adjacent normal is reported. Table S4. Significant CpGs by PAM analysis. For each CpG significant in the PAM analysis, the cancer type the CpG was significant for, and the associated gene is reported. Table S5. Model sensitivity by cancer stage for five CpG multiple cancer models. For each cancer subtype, model sensitivity was calculated by stage for TCGA patients. Asterisks (*) indicate the inclusion of patients in the total without a specified cancer stage: ccRCC (n = 11), pRCC (n = 38), chRCC (n = 2), and total (n = 51). Model sensitivity in tumors smaller than 4 cm (T1a) was also calculated for each subtype: ccRCC (0.92, n = 125), pRCC (0.92, n = 37), chRCC (1.00, n = 5), and total (0.92, n = 167). Table S6. Model sensitivity by cancer stage for four CpG [file 12916_2014_235_MOESM1_ESM.pdf]

Tumor ID#	Normal ID#	Age	Gender	Surgical type	Tumor type	Grade	Stage	Margins	Tumor size (cm)	Recurrence status	Status code	Time FU event (mos)	Time death (mos)
98	100	72	F	Open NX	Clear cell RCC	2	3	Neg	12.00	NED	0	1	N/A
177	178	45	M	Open NX	Clear cell RCC	2	1	Neg	4.50	NED	0	110	N/A
257	258	65	M	Open NX	Clear cell RCC	3	2	Neg	10.30	NED	0	128	N/A
282	281	72	M	Open NX	Tubulopapillary RCC	2	4	Neg	3	UNK	N/A	N/A	N/A
334	337	44	F	Open NX	Unclass. RCC Oncocytic	3	3	Neg	20.00	NED	0	59	N/A
512	511	87	F	Open NX	Clear cell RCC	3	2	Neg	9	NED	0	103	N/A
525	526	71	M	Open NX	Chromophobe RCC	N/A	3	Neg	4.00	NED	0	125	N/A
596	597	54	F	Open NX	Clear cell RCC	3	3	Neg	6.00	AWD	1	1	N/A
655	656	60	M	Open NX	Sarcomatoid/chrom.	4	4	Neg?	22.00	UNK	N/A	N/A	N/A
695	696	58	F	Open NX	Clear cell RCC	2	3	Pos	5	UNK	N/A	N/A	N/A
997	996	2	M	Open NX	Wilms tumor	low	2	Neg	17.00	UNK	N/A	N/A	N/A
1017	1018	77	M	Open NX	Clear cell RCC	3	2	Neg	10	NED	0	12	N/A
1026	1027	77	F	Open NX	Clear cell RCC	2	3	Neg	2.50	NED	0	5	N/A
1060	1061	39	F	Open NX	Clear cell RCC	2	1	Neg	5.30	AWD	1	91	N/A
1221	1222	71	F	Open NX	Papillary	2	1	Neg	4.5	NED	0	115	N/A
1266	1267	66	F	Open NX	Clear cell RCC	3	3	Neg	9.50	DOD	2	2	4
1381	1382	73	F	Open NX	Transitional cell CA	High	3	Neg	6.50	DOD	2	19	42
1534	1535	69	F	Lap NX	Clear cell RCC	2	1	Neg	3.00	NED	0	1	N/A
1610	1611	65	F	Open NX	Chromophobe	2	2	Neg	10.00	NED	0	62	N/A
1615	1616	57	M	Open NX	Papillary RCC	1	2	Neg	4.80	NED	0	108	N/A
1709	1710	69	M	Open NX	Clear cell RCC	4	2	Neg	8.00	NED	0	108	N/A
1944	1945	69	M	Open NX	Clear cell RCC	3	4	Neg	10.50	DOD	2	17	24
1967	1968	78	M	Open NX	Papillary RCC	3	4	Neg	10.50	DOD	2	17	40
2019	2020	70	M	Open NX	Papillary RCC	2	1	Neg	3.50	UNK	N/A	N/A	N/A
2297	2298	47	M	Open NX	Clear cell RCC	2	2	Neg	9.00	NED	0	69	N/A
2435	2436	70	F	Open NX	Chromophobe RCC	2	3	Neg	6.50	NED	0	59	N/A
2575	2576	35	F	Rad NX	Clear cell RCC	2	1	Neg	5.00	NED	0	6	N/A
2589	2590	52	F	Open NX	Clear cell RCC	2	1	Neg	2.00	NED	0	3	N/A
2765	2766	61	M	Lap NX	Clear cell RCC	3	2	Neg	7.00	AWD	1	12	N/A
2800	2801	56	F	Open NX	Chromophobe	1	2	Neg	8.4	NED	0	60	N/A
2845	2846	53	F	Lap NX	Chromophobe	2	1	Neg	3.5	NED	0	75	N/A
2848	2849	49	M	Open NX	Clear cell RCC	2	1	Neg	5.30	NED	0	17	N/A
2864	2865	58	M	Open NX	Clear/sarcomatoid	4	4	Neg	12.00	DOD	2	0	38
2886	2887	66	M	Lap NX	Clear cell RCC	2	1	Neg	4.00	NED	0	83	N/A
2901	2902	75	M	Open NX	Clear cell RCC	3	3	Neg	8.00	NED	0	81	N/A
3015	3016	72	M	Open NX	Clear cell RCC	2	2	Neg	8	UNK	N/A	N/A	N/A
3022	3023	51	F	Open NX	Sarcomatoid/Chrom.	4	3	Neg	17.00	AWD	1	6	N/A
3033	3034	74	M	Open NX	Sarcomatoid	4	4	Pos	13.50	DOD	2	1	12
3035	3036	62	M	Open NX	Oncocytoma	1	2	Neg	9.80	NED	0	3	N/A
3057	3058	69	M	Open NX	Clear cell RCC	2	3	Neg	4	NED	0	80	N/A
3064	3065	66	M	Open NX	Clear cell RCC	2	3b	Pos	11.50	AWD	1	17	N/A
3106	3107	61	F	Open NX	Clear cell RCC	3	2	Neg	7.20	DOD	2	5	49
3125	3126	82	M	Open NX	Oncocytoma	1	2	Neg	16.00	UNK	N/A	N/A	N/A
5038	5039	66	M	Open NX	Clear cell RCC	3	3	Neg	4.60	UNK	N/A	N/A	N/A
5049	5050	57	M	Lap NX	Clear/sarcomatoid	4	2	Neg	8.70	DOD	2	17	48
5080	5081	64	M	Open NX	Clear cell RCC	2	4	Neg	10.00	DOD	2	0	13
5083	5084	44	M	Open NX	Clear cell RCC	2	3	Neg	12.50	UNK	N/A	N/A	N/A
5118	5119	58	M	Open NX	Clear cell RCC	4	4	Neg	10.30	DOD	2	0	37
5264	5265	71	F	Lap NX	Chromophobe RCC	N/A	1	Neg	5.00	NED	0	72	N/A
5273	5274	51	M	Lap NX	Clear cell RCC	3	3	Neg	3.50	DOC	3	15	N/A
5299	5300	58	M	Open NX	Clear cell RCC	2	3	Neg	7.10	UNK	N/A	N/A	N/A
5313	5314	59	M	Lap NX	Clear cell RCC	2	3	Neg	9.00	NED	0	72	N/A
5316	5317	57	M	Lap NX	Papillary	2	2	Neg	13.00	NED	0	67	N/A
5353	5354	58	M	Open NX	Clear cell RCC	4	4	Pos	13.00	AWD	1	1	N/A
5360	5361	76	F	Lap NX	Clear cell RCC	2	1	Neg	4.00	NED	0	24	N/A
5555	5556	58	M	Lap NX	Clear cell RCC	2	3	Neg	7.40	NED	0	72	N/A
5557	5558	74	M	Lap NX	Papillary RCC	2	3	Neg	10.00	AWD	1	6	N/A
5566	5567	66	M	Lap NX	Clear cell RCC	2	1	Neg	2.50	NED	0	10	N/A
5589	5590	70	F	Lap NX	Clear cell RCC	4	3	Neg	10	NED	0	1	N/A
5644	5645	36	M	Lap NX	Chromophobe RCC	1	1	Neg	5.20	NED	0	19	N/A
5646	5647	59	M	Lap NX	Tubulopapillary RCC	1	1	Neg	2.00	NED	0	61	N/A
5652	5653	84	F	Lap NX	Clear cell RCC	3	1	Neg	4.40	DOC	3	17	17
5654	5655	59	F	Lap NX	Clear cell RCC	2	1	Neg	5.50	NED	0	45	N/A
5656	5657	74	M	Lap NX	Clear/sarcomatoid	3	1	Neg	6.20	DOD	2	6	10
5865	5866	65	F	Lap NX	Clear cell RCC	2	1	Neg	4.40	UNK	N/A	N/A	N/A
5903	5904	58	M	Lap NX	Clear cell RCC	4	4	Neg	9.20	AWD	1	0	N/A
5989	5990	71	M	Lap NX	Clear cell RCC	2	1	Neg	4.20	NED	0	66	N/A
6069	6070	41	F	Lap NX	Clear cell RCC	2	1	Neg	6.00	NED	0	15	N/A
6099	6100	55	M	Open NX	Clear cell RCC	2	2	Neg	15.40	NED	0	27	N/A
6281	6282	69	M	Lap NX	Oncocytoma	1	1	Neg	2.5	NED	0	60	N/A
6594	6595	35	M	Lap NX	Clear cell RCC	3	1	Neg	4.50	UNK	N/A	N/A	N/A
6679	6680	83	M	Lap NX	Oncocytoma	1	3	Neg	3.9	NED	0	53	N/A
6717	6718	64	F	Lap NX	Clear cell RCC	2	1	Neg	4.10	NED	0	17	N/A
6785	6786	42	F	Open NX	Clear cell RCC	4	3	Neg	12.00	UNK	N/A	N/A	N/A
6788	6789	81	M	Lap NX	Clear cell RCC	2	3	Neg	7.30	NED	0	57	N/A
6816	6817	50	F	Lap NX	Clear cell RCC	2	1	Neg	4.00	DOC	3	4	4
6874	6875	74	F	Open NX	Clear cell RCC	3	3	Neg	10.00	AWD	1	27	N/A
6881	6882	73	M	Open NX	Papillary	1	1	Neg	6	NED	0	53	N/A
6927	6928	84	M	Lap NX	Clear cell RCC	2	1	Neg	6.00	NED	0	30	N/A

6976	6977	51	M	Lap NX	Clear cell RCC	1	1	Neg	2.90	UNK	N/A	N/A	N/A
7026	7027	56	M	Lap NX	Clear cell RCC	3	3	Neg	5.60	DOD	2	14	52
7057	7058	81	M	Lap NX	Clear cell RCC	3	1	Neg	6.20	UNK	N/A	N/A	N/A
7073	7074	57	F	Open NX	Clear cell RCC	3	4	Neg	6.50	AWD	1	0	N/A
7352	7353	55	M	Lap NX	Clear cell RCC	2	1	Neg	3.70	NED	0	31	N/A
7440	7441	49	F	Open NX	Chromophobe	N/A	2	Neg	12.50	UNK	N/A	N/A	N/A
7475	7476	86	M	Lap NX	Tubulopapillary RCC	2	1	Neg	3.90	DOC	3	10	10
7480	7481	65	M	Lap NX	Clear cell RCC	2	3	Neg	4.80	NED	0	45	N/A
7483	7484	57	M	Open NX	Clear cell RCC	4	2	Neg	9.00	UNK	N/A	N/A	N/A
7654	7655	53	F	Open NX	Clear cell RCC	3	2	Neg	8.00	NED	0	41	N/A
7689	7690	70	M	Lap NX	Clear/sarcomatoid	4	1	Neg	6.20	DOD	2	6	38
0-1-1069	0-1-1070	86	F	Open NX	Clear cell RCC	3	3	Neg	8.80	NED	0	1	N/A
0-1-1235	0-1-1029	58	M	Open NX	Clear cell RCC	2	1	Neg	7.50	AWD	1	103	N/A
0-1-1312	0-1-1303	62	M	Open NX	Clear cell RCC	3	1	Neg	7	NED	0	120	N/A
0-1-1577	0-1-1590	77	F	Open NX	TCCA/mixed CA	N/A	N/A	Pos	3.30	UNK	N/A	N/A	N/A
0-1-1641	0-1-1634	43	F	Lap NX	Chrom./oncocytic/unclass.	2	1	Neg	4.50	NED	0	130	N/A
0-1-206	0-1-262	44	M	Open NX	Chromophobe RCC	3	2	Neg	7.50	UNK	N/A	N/A	N/A
0-1-433	0-1-414	67	M	Open NX	Clear cell RCC	2	1	Neg	3.00	NED	0	24	N/A
0-1-460	0-1-484	50	M	Open NX	Clear cell RCC	2	3	Pos	6.50	UNK	N/A	N/A	N/A
0-1-672	0-1-673	46	F	Open NX	Clear cell RCC	3	4	Neg	12.00	NED	0	24	N/A
0-1-692	0-1-693	62	M	Open NX	Clear cell RCC	4	3	Neg	10.00	DOD	2	4	22
0-1-71	0-1-241	65	F	Open NX	Clear cell RCC	3	3	Neg	11.00	UNK	N/A	N/A	N/A

Significant LMM Analysis CpGs	Model CpG Significant In
cg11802013	General
cg09017174	General
cg07480567	General
cg19663795	General
cg19228118	General
cg19437319	General
cg11450827	General
cg03214697	General
cg06630567	General
cg25332298	General
cg23187653	General
cg20727362	General
cg14341418	General
cg10150530	General
cg09494546	General
cg09626634	General
cg19328294	General
cg22174355	General
cg08040428	General
cg24898753	General
cg11783497	General
cg21277505	General
cg18942631	General
cg18994063	General
cg20034100	General
cg27596068	General
cg26952188	General
cg01733599	General
cg12846567	General
cg11668512	General
cg27263448	General
cg17452257	General
cg21252483	General
cg17706173	General
cg24498554	General
cg27433062	General
cg19305227	General
cg14802310	General
cg19903333	General
cg09103232	General
cg18070061	General
cg19937039	General
cg12099051	General
cg08032971	General
cg01500097	General
cg16698623	General
cg22054164	General
cg00410895	General
cg20530056	General
cg25551168	General
cg07028869	General
cg04710641	General

cg20876010	General
cg02717866	General
cg07027075	General
cg01053621	General
cg20416179	General
cg11946165	General
cg19764407	General
cg26934034	General
cg13352306	General
cg18596794	General
cg27433088	General
cg10521852	General
cg15637528	General
cg07937272	General
cg15142488	General
cg14772955	General
cg25677688	General
cg11546621	General
cg20782689	General
cg20131968	General
cg24895052	General
cg16984812	General
cg05669210	General
cg07359545	General
cg07740640	General
cg04011402	General
cg04689061	General
cg06213598	General
cg10211252	General
cg05346140	General
cg00323915	General
cg05257479	General
cg17943999	General
cg03177025	General
cg20967220	General
cg13006591	General
cg01344518	General
cg06776256	General
cg14356946	General
cg12402251	General
cg19714296	General
cg16356956	General
cg22753768	General
cg12073594	General
cg20392764	General
cg17625032	General
cg05812599	General
cg27349244	General
cg11376198	General
cg12387247	General
cg10342590	General
cg02141675	General
cg13473336	General

cg00295206	General
cg03406535	General
cg10115873	General
cg14972271	General
cg05266460	General
cg15798153	General
cg14757492	General
cg24922992	General
cg01221484	General
cg01511567	General
cg24019851	General
cg03821311	General
cg13376158	General
cg17279839	General
cg24697184	General
cg05114625	General
cg04350646	General
cg05958352	General
cg03459809	General
cg02286642	General
cg06378107	General
cg16408565	General
cg06415153	General
cg18138552	General
cg04438497	General
cg13127386	General
cg08072716	General
cg16293656	General
cg06546607	General
cg16153267	General
cg04224968	General
cg23698287	General
cg04317940	General
cg19514961	General
cg12243271	General
cg24777710	General
cg18194038	General
cg18952560	General
cg16620032	General
cg09259772	General
cg27440002	General
cg22940988	General
cg02906939	General
cg04752565	General
cg07278207	General
cg01957330	General
cg12106945	General
cg07579404	General
cg27188703	General
cg16774604	General
cg25563456	General
cg12398332	General
cg27584171	General

cg11034861	General
cg16159313	General
cg09325711	General
cg10429262	General
cg13216351	General
cg01275830	General
cg27400772	General
cg05976325	General
cg18474934	General
cg25655096	General
cg13060154	General
cg06832039	General
cg03705396	General
cg15639951	General
cg26335299	General
cg00231644	General
cg07588664	General
cg03521113	General
cg04498679	General
cg23625458	General
cg00811081	General
cg02860543	General
cg02421543	General
cg00887101	General
cg04886198	General
cg05985767	General
cg07992625	General
cg07080946	General
cg11869721	General
cg08899632	General
cg03399971	General
cg10732834	General
cg11780934	General
cg02860342	General
cg24407065	General
cg15700197	General
cg03028472	General
cg09831311	General
cg17338258	General
cg14196790	General
cg23242017	General
cg04950789	General
cg02862835	General
cg14039952	General
cg04582938	General
cg13431205	General
cg15441973	General
cg21932814	General
cg02880176	General
cg01934797	General
cg25808839	General
cg21030598	General
cg17171916	General

cg01986577	General
cg05677402	General
cg18372353	General
cg27619475	General
cg07150830	General
cg10200408	General
cg12080675	General
cg23240961	General
cg17732044	General
cg09000112	General
cg09816471	General
cg09985279	General
cg04736140	General
cg05782445	General
cg16218424	General
cg27024922	General
cg15238200	General
cg21762589	General
cg15534366	General
cg00462994	General
cg23926526	General
cg19945840	General
cg05373276	General
cg07753583	General
cg25682559	General
cg16068780	General
cg24877842	General
cg23606023	General
cg00282347	General
cg06585027	General
cg13669583	General
cg14972143	General
cg20360244	General
cg01797043	General
cg09616556	General
cg14992108	General
cg23746359	General
cg10031456	General
cg02917381	General
cg03483654	General
cg15297650	General
cg10145725	General
cg23001650	General
cg17647273	General
cg11630242	General
cg12398397	General
cg05655556	General
cg13129046	General
cg13474750	General
cg12455562	General
cg05662500	General
cg27377213	General
cg14502651	General

cg01127428	General
cg17505757	General
cg08579995	General
cg05729093	General
cg17524624	General
cg24649641	General
cg25593948	General
cg09985635	General
cg22769406	General
cg17416146	General
cg15134628	General
cg04164824	General
cg10869069	General
cg24056567	General
cg20804555	General
cg14602164	General
cg02460349	General
cg06039624	General
cg03014628	General
cg09060914	General
cg22686523	General
cg11505048	General
cg07640820	General
cg12743894	General
cg16908782	General
cg11518240	General
cg13696012	General
cg23491309	General
cg05294095	General
cg15460516	General
cg17547792	General
cg01677098	General
cg17302852	General
cg12576844	General
cg04894993	General
cg10451565	General
cg05142115	General
cg16847051	General
cg20639263	General
cg27015174	General
cg03165700	General
cg12034229	General
cg19237753	General
cg16614332	General
cg16538604	General
cg12762680	General
cg02007463	General
cg25048564	General
cg23877831	General
cg01758870	General
cg11787522	General
cg03292388	General
cg09234474	General

cg21908259	General
cg20021244	General
cg05964373	General
cg18699916	General
cg17225169	General
cg12781218	General
cg14123992	General
cg08629913	General
cg23397015	General
cg17132967	General
cg07221454	General
cg06379754	General
cg15701622	General
cg15456206	General
cg24378421	General
cg07085271	General
cg13466487	General
cg27015047	General
cg22281207	General
cg17820459	General
cg07487535	General
cg05815906	General
cg12466095	General
cg13183539	General
cg10864501	General
cg07422345	General
cg09618682	General
cg19777783	General
cg23452458	General
cg15301700	General
cg02342494	General
cg13878010	General
cg01409734	General
cg06095560	General
cg08839620	General
cg17688525	General
cg23147597	General
cg25960567	General
cg24888049	General
cg15411984	General
cg06626655	General
cg11913104	General
cg06043042	General
cg11166252	General
cg18071588	General
cg16491909	General
cg17026456	General
cg04662451	General
cg10731022	General
cg24934431	General
cg01120307	General
cg02077793	General
cg21597649	General

cg21818252	General
cg12004730	General
cg17356181	General
cg27258787	General
cg16816226	General
cg18222235	General
cg25683185	General
cg16107172	General
cg03792199	General
cg08614528	General
cg00136736	General
cg21885525	General
cg11386746	General
cg23792978	General
cg13726463	General
cg08519865	General
cg08465862	General
cg17834752	General
cg23815000	General
cg04816348	General
cg09794131	General
cg03662459	General
cg10935064	General
cg15619125	General
cg26788107	General
cg24003542	General
cg21874193	General
cg23164327	General
cg06242827	General
cg05654164	General
cg10442358	General
cg24635866	General
cg02200132	General
cg22424444	General
cg11481490	General
cg07586911	General
cg06088032	General
cg07465480	General
cg08719486	General
cg01334437	General
cg05079794	General
cg09087966	General
cg25842633	General
cg13153865	General
cg11653271	General
cg12393697	General
cg14724364	General
cg19951443	General
cg21060854	General
cg24511869	General
cg15540054	General
cg11594415	General
cg23555120	General

cg16319578	General
cg15279364	General
cg15057581	General
cg11730691	General
cg14706739	General
cg27504991	General
cg08608800	General
cg25202404	General
cg02077702	General
cg11084035	General
cg10784067	General
cg04076481	General
cg07707498	General
cg05486551	General
cg09781054	General
cg17729667	General
cg00537910	General
cg21949305	General
cg03241282	General
cg14520511	General
cg23261233	General
cg18103150	General
cg01430807	General
cg10994126	General
cg13347296	General
cg20452583	General
cg04063348	General
cg21458116	General
cg21230435	General
cg08679985	General
cg11649846	General
cg04757243	General
cg09730836	General
cg08709276	General
cg03718539	General
cg14119774	General
cg27167601	General
cg01513611	General
cg08612871	General
cg10190509	General
cg22722802	General
cg25593040	General
cg19335327	General
cg01994779	General
cg16178491	General
cg16268563	General
cg10784813	General
cg06493386	General
cg09721427	General
cg16884042	General
cg07818646	General
cg07617246	General
cg00406188	General

cg15936718	General
cg06255524	General
cg26222229	General
cg14427173	General
cg06766367	General
cg06098051	General
cg27440834	General
cg12249575	General
cg06836736	General
cg15957394	General
cg20831492	General
cg13369981	General
cg04118154	General
cg26277709	General
cg26609691	General
cg01909487	General
cg06864275	General
cg22933847	General
cg14289511	General
cg09618387	General
cg25915982	General
cg13980719	General
cg10154655	General
cg05522288	General
cg11075556	General
cg15443822	General
cg13581475	General
cg08373610	General
cg23376526	General
cg17829314	General
cg22607339	General
cg24649713	General
cg25685640	General
cg00338893	General
cg10944783	General
cg19007602	General
cg19896347	General
cg25277950	General
cg04286933	General
cg24219058	General
cg06885782	General
cg01653110	General
cg21264329	General
cg13847113	General
cg06383088	General
cg18607470	General
cg10016608	General
cg25414165	General
cg14698961	General
cg23456306	General
cg01804844	General
cg12897458	General
cg10309835	General

cg00954003	General
cg11628487	General
cg13332925	General
cg19162158	General
cg08525145	General
cg05674036	General
cg11249173	General
cg24043307	General
cg16786703	General
cg00186954	General
cg08718071	General
cg10551759	General
cg25159610	General
cg20559736	General
cg04444006	General
cg11808757	General
cg24653967	General
cg10457895	General
cg23154272	General
cg26137790	General
cg22730042	General
cg15448599	General
cg02387613	General
cg01875775	General
cg10968815	General
cg11468711	General
cg01264826	General
cg06100324	General
cg14046365	General
cg08453021	General
cg00076645	General
cg15397506	General
cg21804950	General
cg02628930	General
cg20011974	General
cg13682722	General
cg20573242	General
cg07455279	General
cg19830147	General
cg25563642	General
cg02131853	General
cg10764357	General
cg10574499	General
cg27177839	General
cg08704884	General
cg15107670	General
cg01515426	General
cg16746737	General
cg22282941	General
cg11696575	General
cg16770799	General
cg05157725	General
cg12029639	General

cg26668713	General
cg07777378	General
cg19450025	General
cg06493994	General
cg06445611	General
cg08441170	General
cg05520656	General
cg20546002	General
cg21062347	General
cg11983245	General
cg23032316	General
cg10467681	General
cg08128768	General
cg05868316	General
cg22074666	General
cg10906135	General
cg26847093	General
cg08805338	General
cg13969584	General
cg17397493	General
cg15842483	General
cg04969808	General
cg10262425	General
cg17504464	General
cg18343862	General
cg11136251	General
cg08810779	General
cg14543592	General
cg17886028	General
cg09449490	General
cg09260441	General
cg03316864	General
cg25018329	General
cg24914860	General
cg17009433	General
cg03774732	General
cg09694403	General
cg04745805	General
cg17283268	General
cg13668397	General
cg04598744	General
cg27118825	General
cg23940655	General
cg16863447	General
cg12788313	General
cg19486271	General
cg02756845	General
cg11159727	General
cg13551243	General
cg26139360	General
cg07143898	General
cg05857825	General
cg06344807	General

cg13856728	General
cg11203041	General
cg16007680	General
cg09663323	General
cg10714284	General
cg10789261	General
cg05911610	General
cg00545573	General
cg20235051	General
cg00280814	General
cg05046097	General
cg26649834	General
cg24579896	General
cg00519627	General
cg08761447	General
cg24365867	General
cg17905198	General
cg22336401	General
cg22475430	General
cg12663253	General
cg09147213	General
cg26502594	General
cg16953064	General
cg25946758	General
cg14201417	General
cg21096915	General
cg15287183	General
cg19822214	General
cg01602430	General
cg06643227	General
cg11212312	General
cg12787624	General
cg01479232	General
cg03510310	General
cg12920798	General
cg26478992	General
cg22520471	General
cg09498007	General
cg07000831	General
cg11128808	General
cg22063653	General
cg23657252	General
cg13463367	General
cg16236759	General
cg25156443	General
cg18112005	General
cg03075662	General
cg10500283	General
cg15612063	General
cg01431114	General
cg27477990	General
cg20913782	General
cg16075384	General

cg12858460	General
cg13382714	General
cg22325715	General
cg16046465	General
cg20131596	General
cg10478221	General
cg08430598	General
cg23230018	General
cg23881971	General
cg26910651	General
cg00381076	General
cg00367438	General
cg04280119	General
cg26267341	General
cg15974062	General
cg26519339	General
cg19101893	General
cg08935243	General
cg05607127	General
cg15415509	General
cg10301990	General
cg23723933	General
cg25175370	General
cg00782854	General
cg03439805	General
cg02154186	General
cg23824713	General
cg26363196	General
cg00547018	General
cg17378989	General
cg17834443	General
cg26926521	General
cg04402875	General
cg08980578	General
cg04660617	General
cg02456292	General
cg02450959	General
cg12830694	General
cg17948627	General
cg07935151	General
cg25234611	General
cg14334099	General
cg25013852	General
cg13980834	General
cg08015496	General
cg17655576	General
cg26743024	General
cg03833068	General
cg10227731	General
cg07881407	General
cg01288598	General
cg14839257	General
cg09412782	General

cg08552999	General
cg00353953	General
cg15250507	General
cg09674867	General
cg17091770	General
cg04614380	General
cg03813215	General
cg11422541	General
cg10583632	General
cg11677722	General
cg02364038	General
cg07242414	General
cg03731616	General
cg26850503	General
cg24478387	General
cg13613532	General
cg14821083	General
cg00405070	General
cg25459778	General
cg02257681	General
cg08803129	General
cg13703437	General
cg02949544	General
cg25067197	General
cg25307902	General
cg12497298	General
cg15993402	General
cg26408003	General
cg20610545	General
cg03363175	General
cg27606341	General
cg25094569	General
cg11998307	General
cg07155664	General
cg01124961	General
cg23239052	General
cg25565479	General
cg07063745	General
cg12689475	General
cg16793150	General
cg24988345	General
cg19531130	General
cg25055403	General
cg20270599	General
cg01787047	General
cg12991365	General
cg05059825	General
cg03245962	General
cg11951066	General
cg26152597	General
cg10773869	General
cg15860839	General
cg19949550	General

cg20651681	General
cg00426963	General
cg08247612	General
cg26128441	General
cg03312124	General
cg13793354	General
cg21034676	General
cg09054876	General
cg01541629	General
cg09163317	General
cg11957986	General
cg09194436	General
cg22309489	General
cg06536578	General
cg04915182	General
cg06166863	General
cg18712119	General
cg07957491	General
cg11011938	General
cg05659947	General
cg24499411	General
cg14298726	General
cg09585781	General
cg22885821	General
cg08550026	General
cg09259332	General
cg18264687	General
cg09735598	General
cg25853020	General
cg02740128	General
cg13808561	General
cg20300655	General
cg01468621	General
cg04319097	General
cg03427831	General
cg13412063	General
cg26746469	General
cg02874605	General
cg08097755	General
cg05294243	General
cg12554476	General
cg01407797	General
cg23910835	General
cg23242898	General
cg14710524	General
cg01723747	General
cg23250700	General
cg24822446	General
cg21850254	General
cg15002214	General
cg01765461	General
cg19105504	General
cg01078871	General

cg15643724	General
cg22116290	General
cg16225753	General
cg00840516	General
cg16152813	General
cg19567046	General
cg09431062	General
cg04220579	General
cg08496998	General
cg11372831	General
cg05093686	General
cg00834796	General
cg10779183	General
cg17264470	General
cg16933388	General
cg09573435	General
cg25243721	General
cg23858360	General
cg08738269	General
cg10242476	General
cg06762858	General
cg24408511	General
cg05940536	General
cg02664205	General
cg05674944	General
cg11594137	General
cg00824109	General
cg16590151	General
cg12532667	General
cg06905862	General
cg24997562	General
cg18801292	General
cg23499129	General
cg16837769	General
cg01274324	General
cg17733100	General
cg24091474	General
cg15359413	General
cg09559551	General
cg14311811	General
cg01090445	General
cg23412875	General
cg27648946	General
cg25856179	General
cg03429034	General
cg05028306	General
cg15768270	General
cg09480162	General
cg25117976	General
cg26774430	General
cg03586029	General
cg10894512	General
cg15523238	General

cg24043192	General
cg10949322	General
cg16500334	General
cg14026971	General
cg11609154	General
cg18953280	General
cg15576195	General
cg02443089	General
cg17599586	General
cg01651364	General
cg19070338	General
cg22802739	General
cg07577263	General
cg13562911	General
cg11611600	General
cg18388972	General
cg17582250	General
cg20226593	General
cg20482364	General
cg22214414	General
cg11460364	General
cg10377451	General
cg05352541	General
cg12163490	General
cg16257040	General
cg01714932	General
cg20176532	General
cg25298754	General
cg04425624	General
cg12970081	General
cg02159896	General
cg22909962	General
cg19941758	General
cg11336428	General
cg22084336	General
cg07435592	General
cg07780979	General
cg25536300	General
cg27190537	General
cg05671018	General
cg06649520	General
cg03770548	General
cg17711587	General
cg03458191	General
cg06887170	General
cg16293105	General
cg07114894	General
cg02823866	General
cg17351385	General
cg27000831	General
cg04765929	General
cg24353466	General
cg06815817	General

cg25788012	General
cg18694780	General
cg05680424	General
cg01261535	General
cg23307264	General
cg15989091	General
cg05902852	General
cg07269146	General
cg05689121	General
cg07656391	General
cg06919956	General
cg14623518	General
cg16645539	General
cg13120814	General
cg24091698	General
cg26422060	General
cg27318318	General
cg22973042	General
cg02505689	General
cg27254601	General
cg02252907	General
cg22909609	General
cg24731756	General
cg12219753	General
cg18943599	General
cg13514050	General
cg22936016	General
cg04956790	General
cg02200584	General
cg09721659	General
cg09144422	General
cg06665799	General
cg03177084	General
cg19781133	General
cg23411876	General
cg23002907	General
cg01692815	General
cg24219962	General
cg15602969	General
cg06331674	General
cg21802726	General
cg22780428	General
cg22511633	General
cg05422352	General
cg16873863	General
cg18676803	General
cg15021292	General
cg26012103	General
cg11400333	General
cg25018881	General
cg15185001	General
cg27204739	General
cg25817752	General

cg22281080	General
cg06462703	General
cg03168582	General
cg18501026	General
cg00223950	General
cg21518938	General
cg01745657	General
cg14028823	General
cg14532519	General
cg06071083	General
cg11697652	General
cg19058629	General
cg09126273	General
cg01515887	General
cg12548454	General
cg16625901	General
cg00862290	General
cg22228205	General
cg10453040	General
cg10833576	General
cg17740399	General
cg09276883	General
cg11065693	General
cg16120811	General
cg22975712	General
cg11476211	General
cg13439299	General
cg20122491	General
cg09961373	General
cg03996793	General
cg12783776	General
cg16063112	General
cg10326447	General
cg24102360	General
cg11819429	General
cg04916200	General
cg12405833	General
cg19428336	General
cg24687335	General
cg23833452	General
cg08585897	General
cg02904235	General
cg06077733	General
cg12683641	General
cg21494337	General
cg16701105	General
cg26565975	General
cg03354772	General
cg18248891	General
cg01819863	General
cg15627502	General
cg04828892	General
cg11718315	General

cg27242945	General
cg04898512	General
cg23089438	General
cg14282941	General
cg26783353	General
cg15781316	General
cg06291334	General
cg24979630	General
cg03294619	General
cg18619398	General
cg23934633	General
cg19133883	General
cg04251662	General
cg17891123	General
cg05556717	General
cg11528101	General
cg14592406	General
cg20969846	General
cg03438904	General
cg00201234	General
cg13704271	General
cg18700967	General
cg17188169	General
cg19280968	General
cg24205633	General
cg17977362	General
cg15733507	General
cg24441911	General
cg09914773	General
cg24473633	General
cg17414107	General
cg22223602	General
cg07034269	General
cg14338887	General
cg18181161	General
cg19423196	General
cg08783491	General
cg10194829	General
cg09079593	General
cg03580247	General
cg07639959	General
cg12425673	General
cg25741319	General
cg11429632	General
cg19973884	General
cg23972869	General
cg08749122	General
cg07766612	General
cg15396310	General
cg19063972	General
cg21578906	General
cg04455064	General
cg27626424	General

cg00003994	General
cg09462826	General
cg03308985	General
cg20426860	General
cg20919133	General
cg23353982	General
cg03509024	General
cg07446572	General
cg22837289	General
cg13686115	General
cg26171231	General
cg19234509	General
cg04091078	General
cg01959848	General
cg23735442	General
cg27505538	General
cg03681481	General
cg23434459	General
cg20757912	General
cg11649825	General
cg05711886	General
cg12428447	General
cg08120190	General
cg16932672	General
cg23512028	General
cg20717059	General
cg01352882	General
cg26022315	General
cg08244028	General
cg16340268	General
cg00224234	General
cg11960393	General
cg22421699	General
cg17610929	General
cg07186707	General
cg11695601	General
cg27626318	General
cg25923856	General
cg04112019	General
cg01545079	General
cg24101359	General
cg07339327	General
cg22663080	General
cg00598858	General
cg00973334	General
cg00498604	General
cg23364287	General
cg17803807	General
cg12266551	General
cg02863947	General
cg24474998	General
cg22467216	General
cg08736146	General

cg07356771	General
cg21669679	General
cg06760035	General
cg27056853	General
cg13173909	General
cg02161046	General
cg07775675	General
cg09866173	General
cg12971958	General
cg17809798	General
cg12785689	General
cg22766145	General
cg13515674	General
cg15278948	General
cg01776246	General
cg06648029	General
cg13604223	General
cg08584430	General
cg04140754	General
cg20900524	General
cg14659547	General
cg03660451	General
cg03822159	General
cg24352530	General
cg15796978	General
cg03205208	General
cg16695354	General
cg17072268	General
cg22140675	General
cg22640452	General
cg09881917	General
cg11194725	General
cg18022193	General
cg01667702	General
cg26023751	General
cg02344078	General
cg25827666	General
cg21722680	General
cg16257685	General
cg03804985	General
cg22552669	General
cg16382382	General
cg10708675	General
cg17895149	General
cg04422896	General
cg06321883	General
cg26091510	General
cg20748065	General
cg14402472	General
cg22892904	General
cg19413110	General
cg18678763	General
cg25477928	General

cg26003813	General
cg15597540	General
cg19847271	General
cg12932195	General
cg25287375	General
cg15861540	General
cg26872475	General
cg14145762	General
cg17471102	General
cg15417900	General
cg15149938	General
cg09370442	General
cg11158760	General
cg07807817	General
cg08001895	General
cg13615396	General
cg24999924	General
cg19516009	General
cg03359285	General
cg19404832	General
cg22325646	General
cg23022999	General
cg08807541	General
cg20163288	General
cg25425078	General
cg19023700	General
cg10771262	General
cg05131524	General
cg22925639	General
cg20630655	General
cg25680829	General
cg09745307	General
cg15873715	General
cg25943131	General
cg17788832	General
cg09513026	General
cg10633491	General
cg03403539	General
cg20555507	General
cg06803253	General
cg11406695	General
cg07448460	General
cg04345908	General
cg23828595	General
cg02995295	General
cg25602457	General
cg06222120	General
cg14399447	General
cg08047457	General
cg06438300	General
cg02393447	General
cg05038121	General
cg21223353	General

cg22039287	General
cg18081313	General
cg14399656	General
cg04339860	General
cg07220442	General
cg05368089	General
cg03863149	General
cg17277529	General
cg01491225	General
cg16321029	General
cg13890487	General
cg24000189	General
cg19905754	General
cg02890926	General
cg00571634	General
cg03109047	General
cg20319405	General
cg23282559	General
cg03954587	General
cg26781886	General
cg00044245	General
cg13125997	General
cg24264506	General
cg10303487	General
cg16664233	General
cg12241297	General
cg16495265	General
cg07551659	General
cg06139836	General
cg02111587	General
cg18221862	General
cg00611397	General
cg12240824	General
cg23920917	General
cg01848594	General
cg09750183	General
cg07128330	General
cg04349311	General
cg07109287	General
cg20193288	General
cg24438655	General
cg04856203	General
cg25557858	General
cg08452348	General
cg03608974	General
cg08909157	General
cg00988256	General
cg09453737	General
cg10494773	General
cg05990214	General
cg05399697	General
cg03550002	General
cg04269351	General

cg02981703	General
cg19445598	General
cg12825252	General
cg08724636	General
cg19180828	General
cg02028524	General
cg07354440	General
cg06960698	General
cg00291877	General
cg08825571	General
cg13236854	General
cg01293647	General
cg17015234	General
cg13828758	General
cg17063929	General
cg22499237	General
cg14161241	General
cg24385322	General
cg15413566	General
cg17356532	General
cg15937081	General
cg06405090	General
cg14458834	General
cg14147105	General
cg19292008	General
cg26277809	General
cg05554936	General
cg02056135	General
cg05937453	General
cg06605933	General
cg26715540	General
cg15153383	General
cg02668581	General
cg15560112	General
cg08269321	General
cg13121699	General
cg18690395	General
cg15612847	General
cg20241335	General
cg02755525	General
cg08201421	General
cg12821045	General
cg18249244	General
cg25351036	General
cg20887241	General
cg13935507	General
cg21554249	General
cg23048366	General
cg03822011	General
cg15284635	General
cg23610820	General
cg15321195	General
cg02631906	General

cg10149329	General
cg06380007	General
cg20308817	General
cg08287471	General
cg09262269	General
cg04936930	General
cg17606683	General
cg03035419	General
cg17274742	General
cg23189044	General
cg17161537	General
cg13469311	General
cg14631703	General
cg16384137	General
cg01699584	General
cg17257175	General
cg20813773	General
cg07125991	General
cg08634539	General
cg17136126	General
cg18619267	General
cg05497616	General
cg23422659	General
cg10488637	General
cg18226566	General
cg17819635	General
cg25632001	General
cg12107692	General
cg20401181	General
cg15727320	General
cg03812679	General
cg07871503	General
cg22631642	General
cg04600076	General
cg26710722	General
cg15778350	General
cg17522601	General
cg05722918	General
cg00027674	General
cg12867448	General
cg08367026	General
cg23652526	General
cg27416437	General
cg25510610	General
cg17598875	General
cg14654875	General
cg24107581	General
cg25301180	General
cg19037167	General
cg23660999	General
cg15963417	General
cg18393722	General
cg13294594	General

cg17896249	General
cg01876612	General
cg16028934	General
cg01176271	General
cg27303880	General
cg03041841	General
cg22539738	General
cg05032550	General
cg15784615	General
cg09421020	General
cg06463913	General
cg13882835	General
cg18306327	General
cg03679581	General
cg20780953	General
cg26361780	General
cg16169604	General
cg10061138	General
cg23092072	General
cg15683743	General
cg03271651	General
cg08966023	General
cg07200897	General
cg26163537	General
cg05330360	General
cg00834958	General
cg02420102	General
cg03751414	General
cg18020749	General
cg19235307	General
cg17640322	General
cg16969623	General
cg14981132	General
cg05637892	General
cg07265622	General
cg06698742	General
cg11039072	General
cg01446393	General
cg21803417	General
cg04087608	General
cg22584138	General
cg08952029	General
cg20033731	General
cg25873362	General
cg04876451	General
cg15761233	General
cg17983064	General
cg19538485	General
cg04106641	General
cg15447479	General
cg10883303	General
cg22012054	General
cg03328804	General

cg23771603	General
cg13744070	General
cg03549571	General
cg03716942	General
cg20707333	General
cg23268677	General
cg24110063	General
cg15062795	General
cg17829936	General
cg08804892	General
cg06018072	General
cg01322897	General
cg04483623	General
cg17832162	General
cg23350446	General
cg25995916	General
cg13260633	General
cg12076344	General
cg25806808	General
cg25886284	General
cg23772557	General
cg05945622	General
cg26499611	General
cg27316224	General
cg20537325	General
cg18855178	General
cg13562542	General
cg18027946	General
cg12911791	General
cg01137708	General
cg27446185	General
cg01033938	General
cg24272559	General
cg17470637	General
cg12808992	General
cg18003231	Both
cg03283421	Clear Cell
cg04595372	Clear Cell
cg03483626	Clear Cell
cg08107272	Clear Cell
cg02723533	Clear Cell
cg10468702	Clear Cell
cg10795646	Clear Cell
cg12894629	Clear Cell
cg16427670	Clear Cell
cg19439399	Clear Cell
cg10088985	Clear Cell
cg23173455	Clear Cell
cg25423576	Clear Cell
cg19570545	Clear Cell
cg03569412	Clear Cell
cg19282714	Clear Cell
cg14323928	Clear Cell

cg24480859	Clear Cell
cg10516359	Clear Cell
cg06144905	Clear Cell
cg22968401	Clear Cell
cg08097973	Clear Cell
cg00899659	Clear Cell
cg03993463	Clear Cell
cg19971655	Clear Cell
cg27221338	Clear Cell
cg20173259	Clear Cell
cg23901896	Clear Cell
cg01108476	Clear Cell
cg20774846	Clear Cell
cg21537939	Clear Cell
cg25081201	Clear Cell
cg21615663	Clear Cell
cg12650635	Clear Cell
cg17759354	Clear Cell
cg02491878	Clear Cell
cg00518911	Clear Cell
cg02331561	Clear Cell
cg16297030	Clear Cell
cg24525573	Clear Cell
cg17105014	Clear Cell
cg23801057	Clear Cell
cg11154879	Clear Cell
cg01684579	Clear Cell
cg20769774	Clear Cell
cg27244482	Clear Cell
cg05890484	Clear Cell
cg23732182	Clear Cell
cg11984608	Clear Cell
cg07388493	Clear Cell
cg25263140	Clear Cell
cg12603043	Clear Cell
cg00319692	Clear Cell
cg04653308	Clear Cell
cg22381955	Clear Cell
cg06958211	Clear Cell
cg09529667	Clear Cell
cg19186356	Clear Cell
cg04435377	Clear Cell
cg00075967	Clear Cell
cg18877514	Clear Cell
cg02026235	Clear Cell
cg10482024	Clear Cell
cg06394229	Clear Cell
cg12999109	Clear Cell
cg14833385	Clear Cell
cg25025243	Clear Cell
cg17044311	Clear Cell
cg02218324	Clear Cell
cg19921353	Clear Cell

cg03455024	Clear Cell
cg17386185	Clear Cell
cg03964111	Clear Cell
cg15302379	Clear Cell
cg23817637	Clear Cell
cg01186777	Clear Cell
cg08403419	Clear Cell
cg22505977	Clear Cell
cg17361154	Clear Cell
cg26292028	Clear Cell
cg26980692	Clear Cell
cg23192899	Clear Cell
cg05674199	Clear Cell
cg03297731	Clear Cell
cg27146152	Clear Cell
cg15565872	Clear Cell
cg15464148	Clear Cell
cg17971003	Clear Cell
cg00491404	Clear Cell
cg02397720	Clear Cell
cg01169610	Clear Cell
cg26385743	Clear Cell
cg26267310	Clear Cell
cg03855656	Clear Cell
cg03557698	Clear Cell
cg12671744	Clear Cell
cg11158374	Clear Cell
cg08624249	Clear Cell
cg19612574	Clear Cell
cg14425294	Clear Cell
cg21245652	Clear Cell
cg11405695	Clear Cell
cg19339848	Clear Cell
cg02555579	Clear Cell
cg06948937	Clear Cell
cg22340747	Clear Cell
cg15037004	Clear Cell
cg12535715	Clear Cell
cg27089714	Clear Cell
cg09538582	Clear Cell
cg04533291	Clear Cell
cg09360083	Clear Cell
cg13119609	Clear Cell
cg24833277	Clear Cell
cg02280309	Clear Cell
cg04096767	Clear Cell
cg11860203	Clear Cell
cg07361385	Clear Cell
cg17285325	Clear Cell
cg24818418	Clear Cell
cg15381313	Clear Cell
cg02719634	Clear Cell
cg03054529	Clear Cell

cg11360718	Clear Cell
cg13112511	Clear Cell
cg17749456	Clear Cell
cg18382305	Clear Cell
cg19426827	Clear Cell
cg22061523	Clear Cell
cg21129531	Clear Cell
cg06615154	Clear Cell
cg04048249	Clear Cell
cg02915544	Clear Cell
cg18967533	Clear Cell
cg27491887	Clear Cell
cg13553204	Clear Cell
cg11328541	Clear Cell
cg15383087	Clear Cell
cg18992201	Clear Cell
cg20367961	Clear Cell
cg23280807	Clear Cell
cg19096540	Clear Cell
cg16501028	Clear Cell
cg27549944	Clear Cell
cg08831744	Clear Cell
cg10337819	Clear Cell
cg12788467	Clear Cell
cg26457013	Clear Cell
cg07008350	Clear Cell
cg24492022	Clear Cell
cg03404502	Clear Cell
cg11498156	Clear Cell
cg24712395	Clear Cell
cg17720233	Clear Cell
cg00187380	Clear Cell
cg04378886	Clear Cell
cg15296858	Clear Cell
cg03387497	Clear Cell
cg11484576	Clear Cell
cg22986999	Clear Cell
cg20199333	Clear Cell
cg08598221	Clear Cell
cg07371530	Clear Cell
cg07890954	Clear Cell
cg23001457	Clear Cell
cg18172186	Clear Cell
cg05750321	Clear Cell
cg25400358	Clear Cell
cg11072113	Clear Cell
cg06269753	Clear Cell
cg16825643	Clear Cell
cg20202438	Clear Cell
cg15700739	Clear Cell
cg27065979	Clear Cell
cg05722906	Clear Cell
cg14333565	Clear Cell

cg25341653	Clear Cell
cg02192520	Clear Cell
cg02973416	Clear Cell
cg27041096	Clear Cell
cg20777437	Clear Cell
cg01182697	Clear Cell
cg00691625	Clear Cell
cg03679305	Clear Cell
cg24849648	Clear Cell
cg02601403	Clear Cell
cg15822411	Clear Cell
cg24070292	Clear Cell
cg15928132	Clear Cell
cg03294491	Clear Cell
cg01292265	Clear Cell
cg27431396	Clear Cell
cg16983211	Clear Cell
cg23523368	Clear Cell
cg25043279	Clear Cell
cg21678388	Clear Cell
cg05598246	Clear Cell
cg03856723	Clear Cell
cg00597076	Clear Cell
cg04517429	Clear Cell
cg10073042	Clear Cell
cg10179196	Clear Cell
cg19961522	Clear Cell
cg13685294	Clear Cell
cg09205751	Clear Cell
cg15958424	Clear Cell
cg24526899	Clear Cell
cg00174901	Clear Cell
cg22971191	Clear Cell
cg04749372	Clear Cell
cg16717225	Clear Cell
cg22956254	Clear Cell
cg04428453	Clear Cell
cg27519140	Clear Cell
cg00673191	Clear Cell
cg25368651	Clear Cell
cg01269795	Clear Cell
cg08924430	Clear Cell
cg10618882	Clear Cell
cg21152662	Clear Cell
cg08359956	Clear Cell
cg08097657	Clear Cell
cg02988947	Clear Cell
cg14540297	Clear Cell
cg21942546	Clear Cell
cg23743114	Clear Cell
cg27272402	Clear Cell
cg20707345	Clear Cell
cg20793071	Clear Cell

cg20576510	Clear Cell
cg01119135	Clear Cell
cg13217373	Clear Cell
cg23807646	Clear Cell
cg26050734	Clear Cell
cg16992787	Clear Cell
cg04143809	Clear Cell
cg13301014	Clear Cell
cg13364756	Clear Cell
cg14106085	Clear Cell
cg20281815	Clear Cell
cg03138091	Clear Cell
cg12815142	Clear Cell
cg06981182	Clear Cell
cg00515905	Clear Cell
cg19770955	Clear Cell
cg21636577	Clear Cell
cg15241708	Clear Cell
cg00551244	Clear Cell
cg26610808	Clear Cell
cg02065795	Clear Cell
cg11903880	Clear Cell
cg04338788	Clear Cell
cg05174079	Clear Cell
cg27285056	Clear Cell
cg10787197	Clear Cell
cg10057218	Clear Cell
cg21022435	Clear Cell
cg05507459	Clear Cell
cg15187606	Clear Cell
cg24579667	Clear Cell
cg03440846	Clear Cell
cg12237269	Clear Cell
cg18047970	Clear Cell
cg25953146	Clear Cell
cg16449972	Clear Cell
cg12619162	Clear Cell
cg26087862	Clear Cell
cg27443224	Clear Cell
cg13509147	Clear Cell
cg03380645	Clear Cell
cg01820777	Clear Cell
cg11492403	Clear Cell
cg07991621	Clear Cell
cg22721827	Clear Cell
cg17903316	Clear Cell
cg18253802	Clear Cell
cg21550483	Clear Cell
cg07351267	Clear Cell
cg20059312	Clear Cell
cg01252496	Clear Cell
cg10917602	Clear Cell
cg17820591	Clear Cell

cg20289949	Clear Cell
cg11204562	Clear Cell
cg15467759	Clear Cell
cg24652919	Clear Cell
cg20630386	Clear Cell
cg18680834	Clear Cell
cg06616245	Clear Cell
cg12582008	Clear Cell
cg08404227	Clear Cell
cg01333011	Clear Cell
cg23495733	Clear Cell
cg22546318	Clear Cell
cg25668626	Clear Cell
cg07763768	Clear Cell
cg18003698	Clear Cell
cg20645065	Clear Cell
cg06550629	Clear Cell
cg08678755	Clear Cell
cg13486556	Clear Cell
cg06241300	Clear Cell
cg14069619	Clear Cell
cg07086380	Clear Cell
cg06491116	Clear Cell
cg23286660	Clear Cell
cg24012708	Clear Cell
cg01103730	Clear Cell
cg22705225	Clear Cell
cg08116137	Clear Cell
cg07977490	Clear Cell
cg03017264	Clear Cell
cg05442902	Clear Cell
cg05819268	Clear Cell
cg17904739	Clear Cell
cg03386903	Clear Cell
cg14324200	Clear Cell
cg08113203	Clear Cell
cg27019278	Clear Cell
cg15439078	Clear Cell
cg08263647	Clear Cell
cg17500962	Clear Cell
cg27219973	Clear Cell
cg05185584	Clear Cell
cg14615807	Clear Cell
cg23696886	Clear Cell
cg15869642	Clear Cell
cg22083798	Clear Cell
cg06190732	Clear Cell
cg10922280	Clear Cell
cg08927738	Clear Cell
cg23913400	Clear Cell
cg25782229	Clear Cell
cg03330516	Clear Cell
cg07543883	Clear Cell

cg15312323	Clear Cell
cg04907257	Clear Cell
cg25011395	Clear Cell
cg21358381	Clear Cell
cg10725344	Clear Cell
cg00626466	Clear Cell
cg19107595	Clear Cell
cg03166779	Clear Cell
cg25552889	Clear Cell
cg05449607	Clear Cell
cg21459867	Clear Cell
cg26385286	Clear Cell
cg06855803	Clear Cell
cg23613030	Clear Cell
cg14424579	Clear Cell
cg08779777	Clear Cell
cg02675652	Clear Cell
cg19034028	Clear Cell
cg21792737	Clear Cell
cg03245641	Clear Cell
cg03364781	Clear Cell
cg04106785	Clear Cell
cg26825412	Clear Cell
cg21068030	Clear Cell
cg20721467	Clear Cell
cg25954162	Clear Cell
cg13353683	Clear Cell
cg16334519	Clear Cell
cg01473816	Clear Cell
cg18140857	Clear Cell
cg07193504	Clear Cell
cg27126442	Clear Cell
cg27525902	Clear Cell
cg07842062	Clear Cell
cg16142218	Clear Cell
cg21917349	Clear Cell
cg13044136	Clear Cell
cg10576828	Clear Cell
cg21504624	Clear Cell
cg00433406	Clear Cell
cg09793796	Clear Cell
cg27446233	Clear Cell
cg20277250	Clear Cell
cg19370284	Clear Cell
cg13883681	Clear Cell
cg25599242	Clear Cell
cg01185754	Clear Cell
cg07608333	Clear Cell
cg11761535	Clear Cell
cg11452221	Clear Cell
cg04914105	Clear Cell
cg16907514	Clear Cell
cg21360828	Clear Cell

cg02609880	Clear Cell
cg07067241	Clear Cell
cg07792737	Clear Cell
cg24104611	Clear Cell
cg20169062	Clear Cell
cg18509239	Clear Cell
cg12216205	Clear Cell
cg13204181	Clear Cell
cg19815720	Clear Cell
cg11726593	Clear Cell
cg23978557	Clear Cell
cg22395019	Clear Cell
cg11062095	Clear Cell
cg13578652	Clear Cell
cg11540692	Clear Cell
cg23002761	Clear Cell
cg13066963	Clear Cell
cg03343942	Clear Cell
cg12493160	Clear Cell
cg12319004	Clear Cell
cg21513385	Clear Cell
cg19831077	Clear Cell
cg20792294	Clear Cell
cg07425555	Clear Cell
cg08965324	Clear Cell
cg16986720	Clear Cell
cg17239761	Clear Cell
cg08137716	Clear Cell
cg25539131	Clear Cell
cg20066612	Clear Cell
cg15182360	Clear Cell
cg20090497	Clear Cell
cg12346881	Clear Cell
cg02124291	Clear Cell
cg05468303	Clear Cell
cg15316289	Clear Cell
cg27196467	Clear Cell
cg05111036	Clear Cell
cg01982597	Clear Cell
cg05881762	Clear Cell
cg11715966	Clear Cell
cg03533858	Clear Cell
cg22908581	Clear Cell
cg21695020	Clear Cell
cg20500081	Clear Cell
cg22262964	Clear Cell
cg08179907	Clear Cell
cg25553916	Clear Cell
cg26033681	Clear Cell
cg11719283	Clear Cell
cg02603784	Clear Cell
cg00698688	Clear Cell
cg02740947	Clear Cell

cg04898797	Clear Cell
cg09354267	Clear Cell
cg06572974	Clear Cell
cg19382175	Clear Cell
cg06501084	Clear Cell
cg14960043	Clear Cell
cg01036779	Clear Cell
cg19233472	Clear Cell
cg18433380	Clear Cell
cg09382850	Clear Cell
cg05697231	Clear Cell
cg00579402	Clear Cell
cg13285447	Clear Cell
cg16396948	Clear Cell
cg16879596	Clear Cell
cg24150528	Clear Cell
cg04968426	Clear Cell
cg26294551	Clear Cell
cg19393233	Clear Cell
cg11801374	Clear Cell
cg23124451	Clear Cell
cg06339706	Clear Cell
cg17063201	Clear Cell
cg21295911	Clear Cell
cg14951292	Clear Cell
cg00063144	Clear Cell
cg20075229	Clear Cell
cg08141989	Clear Cell
cg12738007	Clear Cell
cg21750887	Clear Cell
cg14634738	Clear Cell
cg18338021	Clear Cell
cg08121954	Clear Cell
cg27226214	Clear Cell
cg01031400	Clear Cell
cg23384185	Clear Cell
cg05482722	Clear Cell
cg20340242	Clear Cell
cg24951114	Clear Cell
cg15158783	Clear Cell
cg03908676	Clear Cell
cg26023389	Clear Cell
cg21808053	Clear Cell
cg23442323	Clear Cell
cg02238504	Clear Cell
cg25041439	Clear Cell
cg11666924	Clear Cell
cg14401897	Clear Cell
cg23655719	Clear Cell
cg26523005	Clear Cell
cg05636175	Clear Cell
cg07084746	Clear Cell
cg06470471	Clear Cell

cg00158308	Clear Cell
cg25821963	Clear Cell
cg03469082	Clear Cell
cg16112050	Clear Cell
cg03262773	Clear Cell
cg09434995	Clear Cell
cg21229859	Clear Cell
cg21201109	Clear Cell
cg15275890	Clear Cell
cg27631817	Clear Cell
cg10844844	Clear Cell
cg14592065	Clear Cell
cg19601035	Clear Cell
cg03907174	Clear Cell
cg20714328	Clear Cell
cg05111110	Clear Cell
cg01803059	Clear Cell
cg04794268	Clear Cell
cg04182865	Clear Cell
cg02556718	Clear Cell
cg02216250	Clear Cell
cg08649013	Clear Cell
cg00268009	Clear Cell
cg20429911	Clear Cell
cg06778853	Clear Cell
cg20368904	Clear Cell
cg25956985	Clear Cell
cg26764555	Clear Cell
cg22657536	Clear Cell
cg13003163	Clear Cell
cg01056568	Clear Cell
cg22458082	Clear Cell
cg08728865	Clear Cell
cg05723825	Clear Cell
cg07408456	Clear Cell
cg11136562	Clear Cell
cg23871659	Clear Cell
cg21421701	Clear Cell
cg16396488	Clear Cell
cg13140740	Clear Cell
cg21458041	Clear Cell
cg18870231	Clear Cell
cg21279865	Clear Cell
cg14704941	Clear Cell
cg04757093	Clear Cell
cg14049461	Clear Cell
cg26683005	Clear Cell
cg00226904	Clear Cell
cg26717133	Clear Cell
cg11946769	Clear Cell
cg00474004	Clear Cell
cg10707565	Clear Cell
cg27218220	Clear Cell

cg06908474	Clear Cell
cg19700658	Clear Cell
cg27258399	Clear Cell
cg06456031	Clear Cell
cg09546307	Clear Cell
cg01294695	Clear Cell
cg27091787	Clear Cell
cg11781389	Clear Cell
cg26036443	Clear Cell
cg11618577	Clear Cell
cg08554114	Clear Cell
cg18130069	Clear Cell
cg25070010	Clear Cell
cg02727423	Clear Cell
cg04235146	Clear Cell
cg21492378	Clear Cell
cg03362494	Clear Cell
cg06488678	Clear Cell
cg06783668	Clear Cell
cg07967308	Clear Cell
cg21435394	Clear Cell
cg00410576	Clear Cell
cg16400825	Clear Cell
cg21283680	Clear Cell
cg11668844	Clear Cell
cg08424427	Clear Cell
cg25421002	Clear Cell
cg00441136	Clear Cell
cg19731122	Clear Cell
cg07068998	Clear Cell
cg21351102	Clear Cell
cg06284244	Clear Cell
cg10660256	Clear Cell
cg16612699	Clear Cell
cg26718420	Clear Cell
cg20603888	Clear Cell
cg26091981	Clear Cell
cg26870337	Clear Cell
cg06471905	Clear Cell
cg14496375	Clear Cell
cg10453365	Clear Cell
cg20402382	Clear Cell
cg11384427	Clear Cell
cg04845628	Clear Cell
cg25058957	Clear Cell
cg10094443	Clear Cell
cg19357849	Clear Cell
cg09811393	Clear Cell
cg01399317	Clear Cell
cg24552358	Clear Cell
cg22963452	Clear Cell
cg21163415	Clear Cell
cg16357381	Clear Cell

cg02624129	Clear Cell
cg10345936	Clear Cell
cg10493166	Clear Cell
cg24642820	Clear Cell
cg02017041	Clear Cell
cg14462830	Clear Cell
cg19282452	Clear Cell
cg06378976	Clear Cell
cg13520715	Clear Cell
cg19906926	Clear Cell
cg16752583	Clear Cell
cg10124201	Clear Cell
cg18678185	Clear Cell
cg08794763	Clear Cell
cg21905630	Clear Cell
cg23328124	Clear Cell
cg14561282	Clear Cell
cg19680672	Clear Cell
cg05335315	Clear Cell
cg09848420	Clear Cell
cg16729794	Clear Cell
cg05389183	Clear Cell
cg25775449	Clear Cell
cg01605984	Clear Cell
cg26147480	Clear Cell
cg06786424	Clear Cell
cg18081258	Clear Cell
cg07618900	Clear Cell
cg00095526	Clear Cell
cg24355006	Clear Cell
cg01580568	Clear Cell
cg24920358	Clear Cell
cg07131544	Clear Cell
cg26504906	Clear Cell
cg14958018	Clear Cell
cg16585619	Clear Cell
cg20998885	Clear Cell
cg20424530	Clear Cell
cg20570279	Clear Cell
cg10493739	Clear Cell
cg25344367	Clear Cell
cg17010112	Clear Cell
cg25567232	Clear Cell
cg17289734	Clear Cell
cg01168201	Clear Cell
cg26391080	Clear Cell
cg19007731	Clear Cell
cg19782598	Clear Cell
cg04033650	Clear Cell
cg18023080	Clear Cell
cg20322862	Clear Cell
cg08104838	Clear Cell
cg24105685	Clear Cell

cg09473585	Clear Cell
cg07908874	Clear Cell
cg08099701	Clear Cell
cg21207418	Clear Cell
cg18524091	Clear Cell
cg13320626	Clear Cell
cg22117143	Clear Cell
cg24165760	Clear Cell
cg22974215	Clear Cell
cg16715722	Clear Cell
cg24331162	Clear Cell
cg02198582	Clear Cell
cg20366832	Clear Cell
cg08529852	Clear Cell
cg24377133	Clear Cell
cg20630151	Clear Cell
cg00347729	Clear Cell
cg04276232	Clear Cell
cg08747889	Clear Cell
cg15081561	Clear Cell
cg04969878	Clear Cell
cg09382492	Clear Cell
cg09296212	Clear Cell
cg00973286	Clear Cell
cg06785429	Clear Cell
cg16050349	Clear Cell
cg05103623	Clear Cell
cg05799317	Clear Cell
cg25822709	Clear Cell
cg20950277	Clear Cell
cg03557733	Clear Cell
cg07236769	Clear Cell
cg01894895	Clear Cell
cg21367957	Clear Cell
cg06534172	Clear Cell
cg24660086	Clear Cell
cg20500126	Clear Cell
cg26757722	Clear Cell
cg27398499	Clear Cell
cg00062776	Clear Cell
cg22417398	Clear Cell
cg04289385	Clear Cell
cg15361231	Clear Cell
cg10864941	Clear Cell
cg12888039	Clear Cell
cg22290566	Clear Cell
cg13813391	Clear Cell
cg14116596	Clear Cell
cg22247240	Clear Cell
cg06481786	Clear Cell
cg05779272	Clear Cell
cg23761196	Clear Cell
cg02332073	Clear Cell

cg20366831	Clear Cell
cg03740216	Clear Cell
cg12188416	Clear Cell
cg05155595	Clear Cell
cg06905514	Clear Cell
cg16977035	Clear Cell
cg17449882	Clear Cell
cg00164898	Clear Cell
cg05089968	Clear Cell
cg24387380	Clear Cell
cg19403377	Clear Cell
cg04994456	Clear Cell
cg21130124	Clear Cell
cg21369988	Clear Cell
cg15006973	Clear Cell
cg11233228	Clear Cell
cg01074640	Clear Cell
cg07360692	Clear Cell
cg24912560	Clear Cell
cg11724759	Clear Cell
cg16019620	Clear Cell
cg04450876	Clear Cell
cg21012874	Clear Cell
cg16907566	Clear Cell
cg15725933	Clear Cell
cg06220208	Clear Cell
cg15711744	Clear Cell
cg18110535	Clear Cell
cg27239921	Clear Cell
cg27345946	Clear Cell
cg12624641	Clear Cell
cg10245048	Clear Cell
cg25092283	Clear Cell
cg22828602	Clear Cell
cg07930578	Clear Cell
cg04837071	Clear Cell
cg10281770	Clear Cell
cg24429836	Clear Cell
cg05668853	Clear Cell
cg17501569	Clear Cell
cg17907567	Clear Cell
cg04992673	Clear Cell
cg25635500	Clear Cell
cg02192965	Clear Cell
cg19635712	Clear Cell
cg21301440	Clear Cell
cg13102585	Clear Cell
cg11843304	Clear Cell
cg13553498	Clear Cell
cg26704579	Clear Cell
cg27178345	Clear Cell
cg05684195	Clear Cell
cg17749384	Clear Cell

cg16433922	Clear Cell
cg20708411	Clear Cell
cg02247582	Clear Cell
cg10559803	Clear Cell
cg03030757	Clear Cell
cg09408780	Clear Cell
cg23082877	Clear Cell
cg20123891	Clear Cell
cg25836326	Clear Cell
cg10604168	Clear Cell
cg07496902	Clear Cell
cg20550118	Clear Cell
cg25250998	Clear Cell
cg03464655	Clear Cell
cg03875195	Clear Cell
cg06840801	Clear Cell
cg00020533	Clear Cell
cg18611122	Clear Cell
cg19145398	Clear Cell
cg04038932	Clear Cell
cg11826486	Clear Cell
cg23323879	Clear Cell
cg12775479	Clear Cell
cg12799265	Clear Cell
cg10823926	Clear Cell
cg05065037	Clear Cell
cg13791589	Clear Cell
cg02888247	Clear Cell
cg11373429	Clear Cell
cg19237879	Clear Cell
cg11277126	Clear Cell
cg02043477	Clear Cell
cg22476084	Clear Cell
cg24073022	Clear Cell
cg01651593	Clear Cell
cg03017946	Clear Cell
cg18493182	Clear Cell
cg09325101	Clear Cell
cg01892689	Clear Cell
cg25020850	Clear Cell
cg13224710	Clear Cell
cg14902389	Clear Cell
cg25424525	Clear Cell
cg12086773	Clear Cell
cg27387222	Clear Cell
cg07664027	Clear Cell
cg03853987	Clear Cell
cg11015241	Clear Cell
cg25799433	Clear Cell
cg11809091	Clear Cell
cg19564367	Clear Cell
cg05600174	Clear Cell
cg25668368	Clear Cell

cg17346022	Clear Cell
cg17850932	Clear Cell
cg03085377	Clear Cell
cg23679141	Clear Cell
cg17093267	Clear Cell
cg03688818	Clear Cell
cg19308222	Clear Cell
cg14804557	Clear Cell
cg12598198	Clear Cell
cg03777459	Clear Cell
cg25519930	Clear Cell
cg22442090	Clear Cell
cg13382694	Clear Cell
cg19166875	Clear Cell
cg16825000	Clear Cell
cg09805010	Clear Cell
cg09848074	Clear Cell
cg18876189	Clear Cell
cg22718139	Clear Cell
cg07220939	Clear Cell
cg06469542	Clear Cell
cg18023724	Clear Cell
cg22844623	Clear Cell
cg21561142	Clear Cell
cg18303397	Clear Cell
cg13434852	Clear Cell
cg11594228	Clear Cell
cg06233985	Clear Cell
cg11871280	Clear Cell
cg03098721	Clear Cell
cg17237881	Clear Cell
cg17386181	Clear Cell
cg09343150	Clear Cell
cg09169633	Clear Cell
cg04461802	Clear Cell
cg03845435	Clear Cell
cg22476295	Clear Cell
cg05585544	Clear Cell
cg03860768	Clear Cell
cg04551925	Clear Cell
cg17740305	Clear Cell
cg06834261	Clear Cell
cg07373172	Clear Cell
cg05968233	Clear Cell
cg04183425	Clear Cell
cg06748315	Clear Cell
cg05745457	Clear Cell
cg22136365	Clear Cell
cg08955609	Clear Cell
cg15206445	Clear Cell
cg27558666	Clear Cell
cg18428563	Clear Cell
cg03665457	Clear Cell

cg15507817	Clear Cell
cg22105022	Clear Cell
cg17207590	Clear Cell
cg26039806	Clear Cell
cg21519900	Clear Cell
cg13334054	Clear Cell
cg18773223	Clear Cell
cg20176648	Clear Cell
cg07499806	Clear Cell
cg10247252	Clear Cell
cg03919781	Clear Cell
cg00278366	Clear Cell
cg11564670	Clear Cell
cg20661303	Clear Cell
cg21785536	Clear Cell
cg13410437	Clear Cell
cg16474696	Clear Cell
cg24879595	Clear Cell
cg11754206	Clear Cell
cg21167159	Clear Cell
cg11947493	Clear Cell
cg06049972	Clear Cell
cg26709950	Clear Cell
cg01892727	Clear Cell
cg21198021	Clear Cell
cg19657082	Clear Cell
cg07747970	Clear Cell
cg00544557	Clear Cell
cg12598178	Clear Cell
cg01587454	Clear Cell
cg19555986	Clear Cell
cg11903151	Clear Cell
cg24928161	Clear Cell
cg10570177	Clear Cell
cg20340596	Clear Cell
cg16194715	Clear Cell
cg01255591	Clear Cell
cg03278643	Clear Cell
cg18468219	Clear Cell
cg23434919	Clear Cell
cg24981018	Clear Cell
cg25946374	Clear Cell
cg21399079	Clear Cell
cg04298323	Clear Cell
cg00024396	Clear Cell
cg07614786	Clear Cell
cg14483391	Clear Cell
cg13672342	Clear Cell
cg10117369	Clear Cell
cg04774694	Clear Cell
cg21902327	Clear Cell
cg23221013	Clear Cell
cg21493583	Clear Cell

cg00202702	Clear Cell
cg00643392	Clear Cell
cg14934821	Clear Cell
cg04290964	Clear Cell
cg03963654	Clear Cell
cg20811607	Clear Cell
cg18987220	Clear Cell
cg20750215	Clear Cell
cg01342792	Clear Cell
cg16501235	Clear Cell
cg02001410	Clear Cell
cg21584430	Clear Cell
cg11793380	Clear Cell
cg14014613	Clear Cell
cg14292823	Clear Cell
cg01794265	Clear Cell
cg21788470	Clear Cell
cg02071074	Clear Cell
cg07011110	Clear Cell
cg21165219	Clear Cell
cg23683201	Clear Cell
cg19823847	Clear Cell
cg20472189	Clear Cell
cg21817450	Clear Cell
cg24611631	Clear Cell
cg14170423	Clear Cell
cg20609368	Clear Cell
cg06094150	Clear Cell
cg03969906	Clear Cell
cg26381783	Clear Cell
cg10909324	Clear Cell
cg20540428	Clear Cell
cg00209066	Clear Cell
cg10453758	Clear Cell
cg15692239	Clear Cell
cg11221132	Clear Cell
cg13234848	Clear Cell
cg23152772	Clear Cell
cg12360886	Clear Cell
cg25020204	Clear Cell
cg11221513	Clear Cell
cg24866437	Clear Cell
cg04683240	Clear Cell
cg27212977	Clear Cell
cg19252175	Clear Cell
cg00585846	Clear Cell
cg18440048	Clear Cell
cg19465374	Clear Cell
cg16358738	Clear Cell
cg00014085	Clear Cell
cg21215336	Clear Cell
cg18711066	Clear Cell
cg26158980	Clear Cell

cg17043101	Clear Cell
cg21035142	Clear Cell
cg19008809	Clear Cell
cg17675150	Clear Cell
cg01762581	Clear Cell
cg15590526	Clear Cell
cg12762799	Clear Cell
cg15096140	Clear Cell
cg18754342	Clear Cell
cg15926585	Clear Cell
cg23704362	Clear Cell
cg06172871	Clear Cell
cg17711870	Clear Cell
cg11065385	Clear Cell
cg18680021	Clear Cell
cg21625881	Clear Cell
cg26203861	Clear Cell
cg19796273	Clear Cell
cg17775713	Clear Cell
cg12610744	Clear Cell
cg07133445	Clear Cell
cg17830754	Clear Cell
cg23029519	Clear Cell
cg17269548	Clear Cell
cg22827524	Clear Cell
cg12588301	Clear Cell
cg08145590	Clear Cell
cg19254235	Clear Cell
cg07052880	Clear Cell
cg08356693	Clear Cell
cg16225429	Clear Cell
cg11635563	Clear Cell
cg19188060	Clear Cell
cg06513075	Clear Cell
cg09906309	Clear Cell
cg26270746	Clear Cell
cg08093398	Clear Cell
cg00493400	Clear Cell
cg16537367	Clear Cell
cg21755709	Clear Cell
cg23517677	Clear Cell
cg26279025	Clear Cell
cg22740835	Clear Cell
cg07264679	Clear Cell
cg06101212	Clear Cell
cg20324165	Clear Cell
cg14740251	Clear Cell
cg23244421	Clear Cell
cg17746675	Clear Cell
cg18263686	Clear Cell
cg19393006	Clear Cell
cg01705587	Clear Cell
cg02688643	Clear Cell

cg23609672	Clear Cell
cg14898639	Clear Cell
cg04432009	Clear Cell
cg16514995	Clear Cell
cg20543571	Clear Cell
cg22197708	Clear Cell
cg23753610	Clear Cell
cg10645113	Clear Cell
cg26550234	Clear Cell
cg25402049	Clear Cell
cg07114024	Clear Cell
cg15272684	Clear Cell
cg06023257	Clear Cell
cg10608333	Clear Cell
cg14315198	Clear Cell
cg26701826	Clear Cell
cg25830052	Clear Cell
cg27298878	Clear Cell
cg07153965	Clear Cell
cg11375102	Clear Cell
cg14847483	Clear Cell
cg10986043	Clear Cell
cg23264413	Clear Cell
cg22332306	Clear Cell
cg05291178	Clear Cell
cg07874520	Clear Cell
cg22764341	Clear Cell
cg01808706	Clear Cell
cg21302727	Clear Cell
cg02657438	Clear Cell
cg03283694	Clear Cell
cg26323655	Clear Cell
cg16173067	Clear Cell
cg15120497	Clear Cell
cg17527798	Clear Cell
cg26762198	Clear Cell
cg18062196	Clear Cell
cg12062995	Clear Cell
cg21201659	Clear Cell
cg14445076	Clear Cell
cg11849692	Clear Cell
cg05969697	Clear Cell
cg03835296	Clear Cell
cg12400041	Clear Cell
cg12989650	Clear Cell
cg17384214	Clear Cell
cg27356438	Clear Cell
cg11176095	Clear Cell
cg03136712	Clear Cell
cg15146752	Clear Cell
cg05348123	Clear Cell
cg24254120	Clear Cell
cg24014020	Clear Cell

cg04824716	Clear Cell
cg02075593	Clear Cell
cg25629694	Clear Cell
cg07512345	Clear Cell
cg06689536	Clear Cell
cg12995941	Clear Cell
cg09699159	Clear Cell
cg22322184	Clear Cell
cg23855989	Clear Cell
cg19325985	Clear Cell
cg02965078	Clear Cell
cg15755406	Clear Cell
cg18538812	Clear Cell
cg12567315	Clear Cell
cg24278076	Clear Cell
cg02620769	Clear Cell
cg18760752	Clear Cell
cg26045434	Clear Cell
cg20091959	Clear Cell
cg02951021	Clear Cell
cg14365123	Clear Cell
cg01739965	Clear Cell
cg00331237	Clear Cell
cg15538427	Clear Cell
cg20791593	Clear Cell
cg14445437	Clear Cell
cg06890553	Clear Cell
cg21750602	Clear Cell
cg00501366	Clear Cell
cg19642007	Clear Cell
cg01753375	Clear Cell
cg14724265	Clear Cell
cg27187881	Clear Cell
cg23663332	Clear Cell
cg17606785	Clear Cell
cg20573420	Clear Cell
cg10213821	Clear Cell
cg24794992	Clear Cell
cg18943195	Clear Cell
cg06134964	Clear Cell
cg11706911	Clear Cell
cg04622802	Clear Cell
cg03027037	Clear Cell
cg06193397	Clear Cell
cg22044848	Clear Cell
cg10770524	Clear Cell
cg09451092	Clear Cell
cg21022395	Clear Cell
cg15900519	Clear Cell
cg02309431	Clear Cell
cg13448625	Clear Cell
cg07986525	Clear Cell
cg24512303	Clear Cell

cg26454191	Clear Cell
cg12564453	Clear Cell
cg05996042	Clear Cell
cg06983551	Clear Cell
cg06168324	Clear Cell
cg04057106	Clear Cell
cg11206763	Clear Cell
cg11492856	Clear Cell
cg04574507	Clear Cell
cg03802231	Clear Cell
cg07495664	Clear Cell
cg01284619	Clear Cell
cg11762629	Clear Cell
cg27020690	Clear Cell
cg26738080	Clear Cell
cg26096837	Clear Cell
cg26059153	Clear Cell
cg27504117	Clear Cell
cg25459323	Clear Cell
cg04297093	Clear Cell
cg26394940	Clear Cell
cg11784281	Clear Cell
cg07685869	Clear Cell
cg18655584	Clear Cell
cg03377682	Clear Cell
cg03879902	Clear Cell
cg02515725	Clear Cell
cg12067261	Clear Cell
cg21518208	Clear Cell
cg22271212	Clear Cell
cg19035993	Clear Cell
cg05532892	Clear Cell
cg07964538	Clear Cell
cg27160701	Clear Cell
cg09931793	Clear Cell
cg25921910	Clear Cell
cg08149333	Clear Cell
cg13105904	Clear Cell
cg26589285	Clear Cell
cg13236107	Clear Cell
cg21432842	Clear Cell
cg04304927	Clear Cell
cg03907363	Clear Cell
cg23920441	Clear Cell
cg08185241	Clear Cell
cg15389153	Clear Cell
cg08596000	Clear Cell
cg04633384	Clear Cell
cg05921581	Clear Cell
cg02844051	Clear Cell
cg11738543	Clear Cell
cg21685427	Clear Cell
cg19790294	Clear Cell

cg18540305	Clear Cell
cg18940763	Clear Cell
cg12888961	Clear Cell
cg13053396	Clear Cell
cg25759381	Clear Cell
cg03545635	Clear Cell
cg26796283	Clear Cell
cg07478208	Clear Cell
cg02797569	Clear Cell
cg03349251	Clear Cell
cg08775793	Clear Cell
cg24747396	Clear Cell
cg17266238	Clear Cell
cg21363706	Clear Cell
cg07546360	Clear Cell
cg11175476	Clear Cell
cg09464487	Clear Cell
cg12989642	Clear Cell
cg19385139	Clear Cell
cg22403851	Clear Cell
cg07677850	Clear Cell
cg17030820	Clear Cell
cg07366967	Clear Cell
cg18891404	Clear Cell
cg00463848	Clear Cell
cg13802364	Clear Cell
cg10135717	Clear Cell
cg17703658	Clear Cell
cg09497789	Clear Cell
cg25054311	Clear Cell
cg17304433	Clear Cell
cg25077328	Clear Cell
cg13199822	Clear Cell
cg14378057	Clear Cell
cg00410921	Clear Cell
cg15778232	Clear Cell
cg00146096	Clear Cell
cg20331177	Clear Cell
cg00368022	Clear Cell
cg25607161	Clear Cell
cg09002165	Clear Cell
cg12727374	Clear Cell
cg23938476	Clear Cell
cg26102760	Clear Cell
cg08462247	Clear Cell
cg24641352	Clear Cell
cg04473030	Clear Cell
cg01578324	Clear Cell
cg23324787	Clear Cell
cg02198044	Clear Cell
cg15937958	Clear Cell
cg19592945	Clear Cell
cg08647446	Clear Cell

cg17539235	Clear Cell
cg13565723	Clear Cell
cg26074851	Clear Cell
cg21039822	Clear Cell
cg23548920	Clear Cell
cg13530039	Clear Cell
cg15032239	Clear Cell
cg17726022	Clear Cell
cg18113270	Clear Cell
cg07482936	Clear Cell
cg07152925	Clear Cell
cg12780322	Clear Cell
cg13253729	Clear Cell
cg16009558	Clear Cell
cg25377358	Clear Cell
cg23470272	Clear Cell
cg23218877	Clear Cell
cg06852652	Clear Cell
cg09111484	Clear Cell
cg13791658	Clear Cell
cg25852941	Clear Cell
cg14688272	Clear Cell
cg14480463	Clear Cell
cg08440425	Clear Cell
cg16632280	Clear Cell
cg27235662	Clear Cell
cg21380842	Clear Cell
cg11976616	Clear Cell
cg12639234	Clear Cell
cg01427567	Clear Cell
cg24705286	Clear Cell
cg14308452	Clear Cell
cg11743795	Clear Cell
cg08507270	Clear Cell
cg21431091	Clear Cell
cg06500476	Clear Cell
cg19906550	Clear Cell
cg04433306	Clear Cell
cg01837574	Clear Cell
cg09839960	Clear Cell
cg15790852	Clear Cell
cg06796611	Clear Cell
cg20847746	Clear Cell
cg11465971	Clear Cell
cg07550362	Clear Cell
cg05354432	Clear Cell
cg05441133	Clear Cell
cg09837169	Clear Cell
cg01991150	Clear Cell
cg07948472	Clear Cell
cg14603345	Clear Cell
cg17966192	Clear Cell
cg06166767	Clear Cell

cg26415633	Clear Cell
cg04624659	Clear Cell
cg21571937	Clear Cell
cg04686412	Clear Cell
cg00250430	Clear Cell
cg05454446	Clear Cell
cg19859270	Clear Cell
cg07554030	Clear Cell
cg12775613	Clear Cell
cg22619563	Clear Cell
cg24611092	Clear Cell
cg07569756	Clear Cell
cg02545106	Clear Cell
cg01446692	Clear Cell
cg12447832	Clear Cell
cg21484834	Clear Cell
cg22947000	Clear Cell
cg01507173	Clear Cell
cg26767897	Clear Cell
cg27270218	Clear Cell
cg16636571	Clear Cell
cg15051063	Clear Cell
cg17006282	Clear Cell
cg03622689	Clear Cell
cg07886712	Clear Cell
cg22090592	Clear Cell
cg16109297	Clear Cell
cg07535475	Clear Cell
cg06597861	Clear Cell
cg23657409	Clear Cell
cg03293882	Clear Cell
cg18043195	Clear Cell
cg07177852	Clear Cell
cg00796728	Clear Cell
cg13488201	Clear Cell
cg26832211	Clear Cell
cg10966500	Clear Cell
cg14523284	Clear Cell
cg03520136	Clear Cell
cg12261786	Clear Cell
cg01956781	Clear Cell
cg03665605	Clear Cell
cg05433460	Clear Cell
cg08289932	Clear Cell
cg18886444	Clear Cell
cg05069385	Clear Cell
cg25974617	Clear Cell
cg09448875	Clear Cell
cg12854483	Clear Cell
cg26701198	Clear Cell
cg16202564	Clear Cell
cg11442717	Clear Cell
cg03614513	Clear Cell

cg16759954	Clear Cell
cg22845362	Clear Cell
cg17942096	Clear Cell
cg09964921	Clear Cell
cg09515805	Clear Cell
cg02279071	Clear Cell
cg01906055	Clear Cell
cg24778383	Clear Cell
cg22001782	Clear Cell
cg27501380	Clear Cell
cg10064162	Clear Cell
cg13857440	Clear Cell
cg17615007	Clear Cell
cg18312782	Clear Cell
cg12288365	Clear Cell
cg25515063	Clear Cell
cg14550518	Clear Cell
cg09879797	Clear Cell
cg04144788	Clear Cell
cg08615333	Clear Cell
cg05473871	Clear Cell
cg23320056	Both
cg13156411	Both
cg12939547	Both
cg14391855	Both
cg14681629	Both
cg25473396	Both
cg26322315	Both
cg24391122	Both
cg02706881	Both
cg13479669	Both
cg12820608	Both
cg14175438	Both
cg14456683	Both
cg05379350	Both
cg06349174	Both
cg22719623	Both
cg10073091	Both
cg00623593	Both
cg03016571	Both
cg26514492	Both
cg22468123	Both
cg15484375	Both
cg03077492	Both
cg04511534	Both
cg13699808	Both
cg06800962	Both
cg12907644	Both
cg06130787	Both
cg19853703	Both
cg25912717	Both
cg12782180	Both
cg19324627	Both

cg25181284	Both
cg18328190	Both
cg02148834	Both
cg07195577	Both
cg04598121	Both
cg13271963	Both
cg04291079	Both
cg18715299	Both
cg06806080	Both
cg19378039	Both
cg13745870	Both
cg04988978	Both
cg00911351	Both
cg00933411	Both
cg25514503	Both
cg17217677	Both
cg00319761	Both
cg05125838	Both
cg08468689	Both
cg02945646	Both
cg08749917	Both
cg16592658	Both
cg04057858	Both
cg12111714	Both
cg22628873	Both
cg17141902	Both
cg25985778	Both
cg07922606	Both
cg11052143	Both
cg08872550	Both
cg08491125	Both
cg23918047	Both
cg13434842	Both
cg23095584	Both
cg24046474	Both
cg20972553	Both
cg00240880	Both
cg27389185	Both
cg02641560	Both
cg10019507	Both
cg09421562	Both
cg25564800	Both
cg08062469	Both
cg03562120	Both
cg09643544	Both
cg07455975	Both
cg03890877	Both
cg07009002	Both
cg15571154	Both
cg02510853	Both
cg05140736	Both
cg22182975	Both
cg22335340	Both

cg20654468	Both
cg11207564	Both
cg07330329	Both
cg10500167	Both
cg17861230	Both
cg05554718	Both
cg03975694	Both
cg16853860	Both
cg21275690	Both
cg25856811	Both
cg14982472	Both
cg07675682	Both
cg09813840	Both
cg02712165	Both
cg20283107	Both
cg09462575	Both
cg25942450	Both
cg02334775	Both
cg09632136	Both
cg00891541	Both
cg09340639	Both
cg04268405	Both
cg01437411	Both
cg05275605	Both
cg10900550	Both
cg21301148	Both
cg15842276	Both
cg26775866	Both
cg16616769	Both
cg19395441	Both
cg05840553	Both
cg00948500	Both
cg22467534	Both
cg19464944	Both
cg05127924	Both
cg15129294	Both
cg17993949	Both
cg00577167	Both
cg02614818	Both
cg23408620	Both
cg17469978	Both
cg14652095	Both
cg11009736	Both
cg27248887	Both
cg21529807	Both
cg06303238	Both
cg10045881	Both
cg27285616	Both
cg15633390	Both
cg26954174	Both
cg22074858	Both
cg25420952	Both
cg19118077	Both

cg09873258	Both
cg02288165	Both
cg19890739	Both
cg21960882	Both
cg19211800	Both
cg12864235	Both
cg11921829	Both
cg08109815	Both
cg11098259	Both
cg06627364	Both
cg01726775	Both
cg13686042	Both
cg23665568	Both
cg02994956	Both
cg11832722	Both
cg27409364	Both
cg03945021	Both
cg04444771	Both
cg24130010	Both
cg08859675	Both
cg18573251	Both
cg14209518	Both
cg23841186	Both
cg25457027	Both
cg10667970	Both
cg19352038	Both
cg12448933	Both
cg03954858	Both
cg16937611	Both
cg24392479	Both
cg16692277	Both
cg13615963	Both
cg07533148	Both
cg12380764	Both
cg18750756	Both
cg16776717	Both
cg21096591	Both
cg06092815	Both
cg23651728	Both
cg23629496	Both
cg13519373	Both
cg19103704	Both
cg07665060	Both
cg07713361	Both
cg02121427	Both
cg20279283	Both
cg07426960	Both
cg10556064	Both
cg01261503	Both
cg21410991	Both
cg15748507	Both
cg19728382	Both
cg11300809	Both

cg20366906	Both
cg12406559	Both
cg21023770	Both
cg04915566	Both
cg05221167	Both
cg00489401	Both
cg24388263	Both
cg04151683	Both
cg16812893	Both
cg05419984	Both
cg03355526	Both
cg20822628	Both
cg25842356	Both
cg08090640	Both
cg03875678	Both
cg15005385	Both
cg18328933	Both
cg19008097	Both
cg07339138	Both
cg11393848	Both
cg21075829	Both
cg19853760	Both
cg04228042	Both
cg02250594	Both
cg11314684	Both
cg25902889	Both
cg07463059	Both
cg19280776	Both
cg27022827	Both
cg02493771	Both
cg20052718	Both
cg22150335	Both
cg02919422	Both
cg00343092	Both
cg09210315	Both
cg24352499	Both
cg09744051	Both
cg10528989	Both
cg01262913	Both
cg03963198	Both
cg06148264	Both
cg13288195	Both
cg10001720	Both
cg16890093	Both
cg22478614	Both
cg00548268	Both
cg01472101	Both
cg04452095	Both
cg22262140	Both
cg15679098	Both
cg08972170	Both
cg24471894	Both
cg07369274	Both

cg09053680	Both
cg16639595	Both
cg12014417	Both
cg02067021	Both
cg21870884	Both
cg16543027	Both
cg08886154	Both
cg03330678	Both
cg27418851	Both
cg11009596	Both
cg25949363	Both
cg13899108	Both
cg21832150	Both
cg20798152	Both
cg21615171	Both
cg19168338	Both
cg04809787	Both
cg00662556	Both
cg22752533	Both
cg25657700	Both
cg00363813	Both
cg22815214	Both
cg04000821	Both
cg25044651	Both
cg22012981	Both
cg17586860	Both
cg04384208	Both
cg03815350	Both
cg21870662	Both
cg23228178	Both
cg04138756	Both
cg18691434	Both
cg07271264	Both
cg01353448	Both
cg02489552	Both
cg15664905	Both
cg26574610	Both
cg24884084	Both
cg19787037	Both
cg02876062	Both
cg00658007	Both
cg25259754	Both
cg25734864	Both
cg00953256	Both
cg25720804	Both
cg05057057	Both
cg02008154	Both
cg25802093	Both
cg07832674	Both
cg06995715	Both
cg23704082	Both
cg18752854	Both
cg13697387	Both

cg04037228	Both
cg13351406	Both
cg10273210	Both
cg24926276	Both
cg02805028	Both
cg24919884	Both
cg26366091	Both
cg16232126	Both
cg09786257	Both
cg24989962	Both
cg04217218	Both
cg08572611	Both
cg16154416	Both
cg25764191	Both
cg26312150	Both
cg12368241	Both
cg27223047	Both
cg25249068	Both
cg13781408	Both
cg03811478	Both
cg20678353	Both
cg13853761	Both
cg22244122	Both
cg11368643	Both
cg20610181	Both
cg00565688	Both
cg24167841	Both
cg06566994	Both
cg14437986	Both
cg19014419	Both
cg07103493	Both
cg15439862	Both
cg12228229	Both
cg14166009	Both
cg11854007	Both
cg17258195	Both
cg23303408	Both
cg02947354	Both
cg00729275	Both
cg17983307	Both
cg23839680	Both
cg05859264	Both
cg04275881	Both
cg23863670	Both
cg20692569	Both
cg26282384	Both
cg10494770	Both
cg08402568	Both
cg11377136	Both
cg01820374	Both
cg13282594	Both
cg14370448	Both
cg06914598	Both

cg13283751	Both
cg05168404	Both
cg19030554	Both
cg09418321	Both
cg18236477	Both
cg21870668	Both
cg20312228	Both
cg06101324	Both
cg10331779	Both
cg18043280	Both
cg06872331	Both
cg20723355	Both
cg09432376	Both
cg04098585	Both
cg04312209	Both
cg21816539	Both
cg02260587	Both
cg10238818	Both
cg02611419	Both
cg16483916	Both
cg21944455	Both
cg12758687	Both
cg18441959	Both
cg02019333	Both
cg20050826	Both
cg10523494	Both
cg11108890	Both
cg05445326	Both
cg22836229	Both
cg08575537	Both
cg05790038	Both
cg04457979	Both
cg12978308	Both
cg23006204	Both
cg18815943	Both
cg23797100	Both
cg05521696	Both
cg22374142	Both
cg03589001	Both
cg20557104	Both
cg03506489	Both
cg24169915	Both
cg06436504	Both
cg16215361	Both
cg24315815	Both
cg02674804	Both
cg05050341	Both
cg15149645	Both
cg21200703	Both
cg26929536	Both
cg14162076	Both
cg03079681	Both
cg24240626	Both

cg09809672	Both
cg21030400	Both
cg08368934	Both
cg11262874	Both
cg09096031	Both
cg13316191	Both
cg10762615	Both
cg02085507	Both
cg00059225	Both
cg26984624	Both
cg14290291	Both
cg12966875	Both
cg09914444	Both
cg14894216	Both
cg09936839	Both
cg12954718	Both
cg17356733	Both
cg25447894	Both
cg20587968	Both
cg12491710	Both
cg16431978	Both
cg22123464	Both
cg25372195	Both
cg25182621	Both
cg09936561	Both
cg02613386	Both
cg07997737	Both
cg12506373	Both
cg22980079	Both
cg15207953	Both
cg03734783	Both
cg27285720	Both
cg10249734	Both
cg01530101	Both
cg14366598	Both
cg20542190	Both
cg07047653	Both
cg27011193	Both
cg07580475	Both
cg00644033	Both
cg06812844	Both
cg09067967	Both
cg02679745	Both
cg24829483	Both
cg17383958	Both
cg23101680	Both
cg02409351	Both
cg11864993	Both
cg06392096	Both
cg13234863	Both
cg21296230	Both
cg08668790	Both
cg17612991	Both

cg22179082	Both
cg17127769	Both
cg26186727	Both
cg24443250	Both
cg20018806	Both
cg06498267	Both
cg23032612	Both
cg15982419	Both
cg24816455	Both
cg06196379	Both
cg01288089	Both
cg08828036	Both
cg07409200	Both
cg03544320	Both
cg20057066	Both
cg00333226	Both
cg24199834	Both
cg18636641	Both
cg07752420	Both
cg27652350	Both
cg23290344	Both
cg09577651	Both
cg11846956	Both
cg17470143	Both
cg08946332	Both
cg08840010	Both
cg01775265	Both
cg18920397	Both
cg03960217	Both
cg10175795	Both
cg04901273	Both
cg21964481	Both
cg16757724	Both
cg00208967	Both
cg22038738	Both
cg27059238	Both
cg07186138	Both
cg08441806	Both
cg06226384	Both
cg10674793	Both
cg06641366	Both
cg00935364	Both
cg13302823	Both
cg09069593	Both
cg10691006	Both
cg19502744	Both
cg02367951	Both
cg23765993	Both
cg07947016	Both
cg17241310	Both
cg25384595	Both
cg16777510	Both
cg12699371	Both

cg02423618	Both
cg13929328	Both
cg20657383	Both
cg14056306	Both
cg13784855	Both
cg09588653	Both
cg03171924	Both
cg04528819	Both
cg24059075	Both
cg16652063	Both
cg21111471	Both
cg14696396	Both
cg20089715	Both
cg18674980	Both
cg02055963	Both
cg14914852	Both
cg23640701	Both
cg05885720	Both
cg06290096	Both
cg21907579	Both
cg13462129	Both
cg21626086	Both
cg00042156	Both
cg05590982	Both
cg05109049	Both
cg16158874	Both
cg13802966	Both
cg25840094	Both
cg23037403	Both
cg19580810	Both
cg27643859	Both
cg27417997	Both
cg20622019	Both
cg22187630	Both
cg24576425	Both
cg14822966	Both
cg05488632	Both
cg23433607	Both
cg03752885	Both
cg25084878	Both
cg01767116	Both
cg24713204	Both
cg08569678	Both
cg17267907	Both
cg24012925	Both
cg08254089	Both
cg14424530	Both
cg19510604	Both
cg18793806	Both
cg26504021	Both
cg04081402	Both
cg08786003	Both
cg13870494	Both

cg17199483	Both
cg18239753	Both
cg16547529	Both
cg21541083	Both
cg03799530	Both
cg18555440	Both
cg01072821	Both
cg26179948	Both
cg25524473	Both
cg08878744	Both
cg11340260	Both
cg10481740	Both
cg16175792	Both
cg24641737	Both
cg14882700	Both
cg01593385	Both
cg07405796	Both
cg26561254	Both
cg15191648	Both
cg26771272	Both
cg03160135	Both
cg27634151	Both
cg22418909	Both
cg25538571	Both
cg22471346	Both
cg26614073	Both
cg19686152	Both
cg02182354	Both
cg12680609	Both
cg23181133	Both
cg16745616	Both
cg06263495	Both
cg07533529	Both
cg09172980	Both
cg25947945	Both
cg24928687	Both
cg20387341	Both
cg17162024	Both
cg07935264	Both
cg16944093	Both
cg07471052	Both
cg05073035	Both
cg14826456	Both
cg04488758	Both
cg13763232	Both
cg20833786	Both
cg20839025	Both
cg13859324	Both
cg12792367	Both
cg02939139	Both
cg06392426	Both
cg05628549	Both
cg21053529	Both

cg15433631	Both
cg19491035	Both
cg03294557	Both
cg09112782	Both
cg16678925	Both
cg23713742	Both
cg07014174	Both
cg11270633	Both
cg15430659	Both
cg10904672	Both
cg12891678	Both
cg10525372	Both
cg17049328	Both
cg24840099	Both
cg16504670	Both
cg13297960	Both
cg22324153	Both
cg26093148	Both
cg08432727	Both
cg12334759	Both
cg06421800	Both
cg22189286	Both
cg02620013	Both
cg10705800	Both
cg05373457	Both
cg25336198	Both
cg12347740	Both
cg21880328	Both
cg24613957	Both
cg14859460	Both
cg19475870	Both
cg02757432	Both
cg13870866	Both
cg26191951	Both
cg11432797	Both
cg09030119	Both
cg11304234	Both
cg00616135	Both
cg11091262	Both
cg17173423	Both
cg25087423	Both
cg15555014	Both
cg03469054	Both
cg14494812	Both
cg16280667	Both
cg12207371	Both
cg22303211	Both
cg14178991	Both
cg10307548	Both
cg21488617	Both
cg13998293	Both
cg15701111	Both
cg20469837	Both

cg12374721	Both
cg00891278	Both
cg14859417	Both
cg27105123	Both
cg12315311	Both
cg26201213	Both
cg18267381	Both
cg10942056	Both
cg26823505	Both
cg10895543	Both
cg26540515	Both
cg11456838	Both
cg01410472	Both
cg26521448	Both
cg27032184	Both
cg06131859	Both
cg04365980	Both
cg00041575	Both
cg24481163	Both
cg11577097	Both
cg04569233	Both
cg24621042	Both
cg27403635	Both
cg16832407	Both
cg01138020	Both
cg02164046	Both
cg18110483	Both
cg02764897	Both
cg23953831	Both
cg26020513	Both
cg23412777	Both
cg21115977	Both
cg16404106	Both
cg12703269	Both
cg19332710	Both
cg09626984	Both
cg03167883	Both
cg19776453	Both
cg24133080	Both
cg04872689	Both
cg12879425	Both
cg19005210	Both
cg02626929	Both
cg08369065	Both
cg07376232	Both
cg00936626	Both
cg15910079	Both
cg24497819	Both
cg07525077	Both
cg24628744	Both
cg21750589	Both
cg20090467	Both
cg18860847	Both

cg00645579	Both
cg21578207	Both
cg15512851	Both
cg00447208	Both
cg17795240	Both
cg02037013	Both
cg25978208	Both
cg07703401	Both
cg06005396	Both
cg07363637	Both
cg25462303	Both
cg16254309	Both
cg08209133	Both
cg02498063	Both
cg15105987	Both
cg14958635	Both
cg14787704	Both
cg09425611	Both
cg26608667	Both
cg19539004	Both
cg06183267	Both
cg01447817	Both
cg13985639	Both
cg11521325	Both
cg26687173	Both
cg09076123	Both
cg05958582	Both
cg04563996	Both
cg04623955	Both
cg26829529	Both
cg27114120	Both
cg07845392	Both
cg22199118	Both
cg20017147	Both
cg01857260	Both
cg22552966	Both
cg10548978	Both
cg11208483	Both
cg16245261	Both
cg16119128	Both
cg19594666	Both
cg17738194	Both
cg12391783	Both
cg15357945	Both
cg05538432	Both
cg14221171	Both
cg11932564	Both
cg19721889	Both
cg12071073	Both
cg19882093	Both
cg14646244	Both
cg17074151	Both
cg27429194	Both

cg08583049	Both
cg04784315	Both
cg01796223	Both
cg19863740	Both
cg16967583	Both
cg26884581	Both
cg24164563	Both
cg09328024	Both
cg08654655	Both
cg23297477	Both
cg24019564	Both
cg04799664	Both
cg17439694	Both
cg08996413	Both
cg11590700	Both
cg16708981	Both
cg11716026	Both
cg24440147	Both
cg14294758	Both
cg24697329	Both
cg19222480	Both
cg02735486	Both
cg13997435	Both
cg15177359	Both
cg10735607	Both
cg07426848	Both
cg15746620	Both
cg12951282	Both
cg14386061	Both
cg12687463	Both
cg15446391	Both
cg20792062	Both
cg27347104	Both
cg08719081	Both
cg24505341	Both
cg07189381	Both
cg19064258	Both
cg12768605	Both
cg06277657	Both
cg19264571	Both
cg00995520	Both
cg20585500	Both
cg07104706	Both
cg18766755	Both
cg15127733	Both
cg26162582	Both
cg05508084	Both
cg04534765	Both
cg20287234	Both
cg04675937	Both
cg01830294	Both
cg05439368	Both
cg25691167	Both

cg08970694	Both
cg21530890	Both
cg12478185	Both
cg20891301	Both
cg19569684	Both
cg10777851	Both
cg05262335	Both
cg17657618	Both
cg27090216	Both
cg13287780	Both
cg08797106	Both
cg13678049	Both
cg01656216	Both
cg26045205	Both
cg18780284	Both
cg10052840	Both
cg02257405	Both
cg07753644	Both
cg25356886	Both
cg09551916	Both
cg02002676	Both
cg11147886	Both
cg25651505	Both
cg04541607	Both
cg25781123	Both
cg09542111	Both
cg12069042	Both
cg03547924	Both
cg20786074	Both
cg13882988	Both
cg03365437	Both
cg26825755	Both
cg05436658	Both
cg09825414	Both
cg22563697	Both
cg24835159	Both
cg00399483	Both
cg03733371	Both
cg17827767	Both
cg17627559	Both
cg21233722	Both
cg19428417	Both
cg12351433	Both
cg01254505	Both
cg00634577	Both
cg21665774	Both
cg09816912	Both
cg23352695	Both
cg20807545	Both
cg25391023	Both
cg13705284	Both
cg23413307	Both
cg16924616	Both

cg24798047	Both
cg09748975	Both
cg27120999	Both
cg09214551	Both
cg14519350	Both
cg16414852	Both
cg04387658	Both
cg05788638	Both
cg13262687	Both
cg16959606	Both
cg13044223	Both
cg06521852	Both
cg21303386	Both
cg04818845	Both
cg25055477	Both
cg10506318	Both
cg00035347	Both
cg18908499	Both
cg14532417	Both
cg08758850	Both
cg13672791	Both
cg18087477	Both
cg27071517	Both
cg17188046	Both
cg23130254	Both
cg15674997	Both
cg12971694	Both
cg11037148	Both
cg14008883	Both
cg06595693	Both
cg25659818	Both
cg11323198	Both
cg13246269	Both
cg07039362	Both
cg11119596	Both
cg02831294	Both
cg03943081	Both
cg12003230	Both
cg11679069	Both
cg15043801	Both
cg15645309	Both
cg11981599	Both
cg20498685	Both
cg02301815	Both
cg13439730	Both
cg07295678	Both
cg23732024	Both
cg10885338	Both
cg00573606	Both
cg00135393	Both
cg12530021	Both
cg12941931	Both
cg19636861	Both

cg03602500	Both
cg00708598	Both
cg04348872	Both
cg24355048	Both
cg16042149	Both
cg17771150	Both
cg14289461	Both
cg17357062	Both
cg20008332	Both
cg03887787	Both
cg11435943	Both
cg13634864	Both
cg16428251	Both
cg26245202	Both
cg26200585	Both
cg05209917	Both
cg24250393	Both
cg22954818	Both
cg25148589	Both
cg19616230	Both
cg02157083	Both
cg00974864	Both
cg07499072	Both
cg04340502	Both
cg12324629	Both
cg16020904	Both
cg21628553	Both
cg26065841	Both
cg18343292	Both
cg05345286	Both
cg04089739	Both
cg05352668	Both
cg13745346	Both
cg14189571	Both
cg02255004	Both
cg04062391	Both
cg12958813	Both
cg16069910	Both
cg22631938	Both
cg27553955	Both
cg27183007	Both
cg19216731	Both
cg19789505	Both
cg24645221	Both
cg12757143	Both
cg16301617	Both
cg24748945	Both
cg03741352	Both
cg20832020	Both
cg00823148	Both
cg14893129	Both
cg23316360	Both
cg25388528	Both

cg26128092	Both
cg24887211	Both
cg08214029	Both
cg24426405	Both
cg07038400	Both
cg10248727	Both
cg23166289	Both
cg09204187	Both
cg15052335	Both
cg02920216	Both
cg00594952	Both
cg07676849	Both
cg05959508	Both
cg01668383	Both
cg07905963	Both
cg25944100	Both
cg26928682	Both
cg11172423	Both
cg09230173	Both
cg17186163	Both
cg19456540	Both
cg27243140	Both
cg12022621	Both
cg17460386	Both
cg18119407	Both
cg16902509	Both
cg20055101	Both
cg00025991	Both
cg03625911	Both
cg09835085	Both
cg03460527	Both
cg03775123	Both
cg26181372	Both
cg24949488	Both
cg11935147	Both
cg18345635	Both
cg02737335	Both
cg12661964	Both
cg03391568	Both
cg07498879	Both
cg09548084	Both
cg09305224	Both
cg22398616	Both
cg25214366	Both
cg25176823	Both
cg08118311	Both
cg09424308	Both
cg21992250	Both
cg04490714	Both
cg07664856	Both
cg10751811	Both
cg23517605	Both
cg04567009	Both

cg14011639	Both
cg09158314	Both
cg03534410	Both
cg03221914	Both
cg22680204	Both
cg26705561	Both
cg02066681	Both
cg10282491	Both
cg11285003	Both
cg26135325	Both
cg17267493	Both
cg18589858	Both
cg00554173	Both
cg07156669	Both
cg21475402	Both
cg03670238	Both
cg25903122	Both
cg04987894	Both
cg16545105	Both
cg25514304	Both
cg26608332	Both
cg05380982	Both
cg23752985	Both
cg03693099	Both
cg12125117	Both
cg07138512	Both
cg21919219	Both
cg10338338	Both
cg12616487	Both
cg19408398	Both
cg08942800	Both
cg22670329	Both
cg14959707	Both
cg07705835	Both
cg24333473	Both
cg05546038	Both
cg17683775	Both
cg14940420	Both
cg22672790	Both
cg25151295	Both
cg17518965	Both
cg26170660	Both
cg11254522	Both
cg22805632	Both
cg24861272	Both
cg03782453	Both
cg27016494	Both
cg18338293	Both
cg24693053	Both
cg02245378	Both
cg25366404	Both
cg05232889	Both
cg18902090	Both

cg21790626	Both
cg25340403	Both
cg15954792	Both
cg16080552	Both
cg09721047	Both
cg04981492	Both
cg01295203	Both
cg25107791	Both
cg20985014	Both
cg10303842	Both
cg11213150	Both
cg18420965	Both
cg17349199	Both
cg26143719	Both
cg20616414	Both
cg06291867	Both
cg02404843	Both
cg01148741	Both
cg15736165	Both
cg09220361	Both
cg17452384	Both
cg18055394	Both
cg12952136	Both
cg22149792	Both
cg18988110	Both
cg09656934	Both
cg10080004	Both
cg22774472	Both
cg08832227	Both
cg21142398	Both
cg01351032	Both
cg13133883	Both
cg17998964	Both
cg07168556	Both
cg05767404	Both
cg23265096	Both
cg23615676	Both
cg00739120	Both
cg05016508	Both
cg23178308	Both
cg02780988	Both
cg27032352	Both
cg27351358	Both
cg12024906	Both
cg02885771	Both
cg13798289	Both
cg02305723	Both
cg03634982	Both
cg10201668	Both
cg01185080	Both
cg10210238	Both
cg19963522	Both
cg09571369	Both

cg03258472	Both
cg11934695	Both
cg19433435	Both
cg26482939	Both
cg21770617	Both
cg19372178	Both
cg03349953	Both
cg09260089	Both
cg21846488	Both
cg06015218	Both
cg22040627	Both
cg18149919	Both
cg20289911	Both
cg10970251	Both
cg04267184	Both
cg25598083	Both
cg17166338	Both
cg10081899	Both
cg22484793	Both
cg15531099	Both
cg05766474	Both
cg11558474	Both
cg18589624	Both
cg07237830	Both
cg17605084	Both
cg22637941	Both
cg24646414	Both
cg15606663	Both
cg17410236	Both
cg17347253	Both
cg17703212	Both
cg06165395	Both
cg06274159	Both
cg11946503	Both
cg03340878	Both
cg21414251	Both
cg00503840	Both
cg22134325	Both
cg18530324	Both
cg26991946	Both
cg12894126	Both
cg12457773	Both
cg17199658	Both
cg15780361	Both
cg15747595	Both
cg03533058	Both
cg10920765	Both
cg17638468	Both
cg02332525	Both
cg11797994	Both
cg02091100	Both
cg05436231	Both
cg00245878	Both

cg25990647	Both
cg06123346	Both
cg16776350	Both
cg24239808	Both
cg12880658	Both
cg12613383	Both
cg20648149	Both
cg26022401	Both
cg02794695	Both
cg03699566	Both
cg21789545	Both
cg22449114	Both
cg15309006	Both
cg07715201	Both
cg02417264	Both
cg14289985	Both
cg23092823	Both
cg23641264	Both
cg26285698	Both
cg14967066	Both
cg05331214	Both
cg26756862	Both
cg10257049	Both
cg24338843	Both
cg24541550	Both
cg03929796	Both
cg00661485	Both
cg13022129	Both
cg20191453	Both
cg10423860	Both
cg01636591	Both
cg06906435	Both
cg13053608	Both
cg26625319	Both
cg26750002	Both
cg09682183	Both
cg16744741	Both
cg05569220	Both
cg01889448	Both
cg14851685	Both
cg01980222	Both
cg06235429	Both
cg13081704	Both
cg15610233	Both
cg09937039	Both
cg10758292	Both
cg09988116	Both
cg11068096	Both
cg23458892	Both
cg09617773	Both
cg05751148	Both
cg17271365	Both
cg12277666	Both

cg22759185	Both
cg12694870	Both
cg00910067	Both
cg14535518	Both
cg13396068	Both
cg07981588	Both
cg15228639	Both
cg26252167	Both
cg20674577	Both
cg24670715	Both
cg10774440	Both
cg25484904	Both
cg00754253	Both
cg00895324	Both
cg19047670	Both
cg23627134	Both
cg20850981	Both
cg04123507	Both
cg17253459	Both
cg23909343	Both
cg04435420	Both
cg22842233	Both
cg06311778	Both
cg14184817	Both
cg12836863	Both
cg24235633	Both
cg02141570	Both
cg11418559	Both
cg06220235	Both
cg03928182	Both
cg26293512	Both
cg22801690	Both
cg19690214	Both
cg16092786	Both
cg14056644	Both
cg03840259	Both
cg24784109	Both
cg05067286	Both
cg09593286	Both
cg22821834	Both
cg24650229	Both
cg01405761	Both
cg07348311	Both
cg17982102	Both
cg16743289	Both
cg18401406	Both
cg12515371	Both
cg22733478	Both
cg09222115	Both
cg09307264	Both
cg08929103	Both
cg22601917	Both
cg26158194	Both

cg21591742	Both
cg11084611	Both
cg26770882	Both
cg14186992	Both
cg21296676	Both
cg07684796	Both
cg01450842	Both
cg20098478	Both
cg01546430	Both
cg16872071	Both
cg08775230	Both
cg02097420	Both
cg16196812	Both
cg12530080	Both
cg25133753	Both
cg16240480	Both
cg13749822	Both
cg20855565	Both
cg21604615	Both
cg12285118	Both
cg13668129	Both
cg17240987	Both
cg00105253	Both
cg21335942	Both
cg03755123	Both
cg19873701	Both
cg14323109	Both
cg02254461	Both
cg05413282	Both
cg13701109	Both
cg23563234	Both
cg03870261	Both
cg08256691	Both
cg20640433	Both
cg10853416	Both
cg09068492	Both
cg16019273	Both
cg22305782	Both
cg07509155	Both
cg18429742	Both
cg11928198	Both
cg10486998	Both
cg04398978	Both
cg01036012	Both
cg10837843	Both
cg19058765	Both
cg04369341	Both
cg09448880	Both
cg02415431	Both
cg14620221	Both
cg23152755	Both
cg16242770	Both
cg21747271	Both

cg02989940	Both
cg16708012	Both
cg12163132	Both
cg15551881	Both
cg14440664	Both
cg26928972	Both
cg14178895	Both
cg10919204	Both
cg06285340	Both
cg02099418	Both
cg00540769	Both
cg03923934	Both
cg17397420	Both
cg14324675	Both
cg10694914	Both
cg21180599	Both
cg24088438	Both
cg18788940	Both
cg13407883	Both
cg10520594	Both
cg08390209	Both
cg03301801	Both
cg19486673	Both
cg05500015	Both
cg22415472	Both
cg01367992	Both
cg00002426	Both
cg07442479	Both
cg13461622	Both
cg17405586	Both
cg03289872	Both
cg08529529	Both
cg01356829	Both
cg16638540	Both
cg12493906	Both
cg00986320	Both
cg04086012	Both
cg23679724	Both
cg04956382	Both
cg25216696	Both
cg26453588	Both
cg18431127	Both
cg00138126	Both
cg18884741	Both
cg15540820	Both
cg21969640	Both
cg13580728	Both
cg10189695	Both
cg13702536	Both
cg27324619	Both
cg22815110	Both
cg10516886	Both
cg17496921	Both

cg04790129	Both
cg18705776	Both
cg16068833	Both
cg10525488	Both
cg14384532	Both
cg03007010	Both
cg05163588	Both
cg10217445	Both
cg13553455	Both
cg22264436	Both
cg21518947	Both
cg16609872	Both
cg05600717	Both
cg22646937	Both
cg27444994	Both
cg12621514	Both
cg12620499	Both
cg21784940	Both
cg27665659	Both
cg19290962	Both
cg17890764	Both
cg24423088	Both
cg16957313	Both
cg10054857	Both
cg11600161	Both
cg03724463	Both
cg25066857	Both
cg19576304	Both
cg15839448	Both
cg26565838	Both
cg17475456	Both
cg08315770	Both
cg11177693	Both
cg08823182	Both
cg16465769	Both
cg19304352	Both
cg06722633	Both
cg07127957	Both
cg25841987	Both
cg03938043	Both
cg11241627	Both
cg13718960	Both
cg15746445	Both
cg02844545	Both
cg26590537	Both
cg19970051	Both
cg25943276	Both
cg24673765	Both
cg14319409	Both
cg23595927	Both
cg20387706	Both
cg08551633	Both
cg19797376	Both

cg22392708	Both
cg20427865	Both
cg10503232	Both
cg26799474	Both
cg26647453	Both
cg05947740	Both
cg20773127	Both
cg26946769	Both
cg27626299	Both
cg01765641	Both
cg21974239	Both
cg21509023	Both
cg05158615	Both
cg26453670	Both
cg01871963	Both
cg16749930	Both
cg01352108	Both
cg22809047	Both
cg02442161	Both
cg09299388	Both
cg07211259	Both
cg10129493	Both
cg07380416	Both
cg17446142	Both
cg12582959	Both
cg14121103	Both
cg01671881	Both
cg02266731	Both
cg09119665	Both
cg25799986	Both
cg24488435	Both
cg13759328	Both
cg20979799	Both
cg23140706	Both
cg02912041	Both
cg14252059	Both
cg02786378	Both
cg25187533	Both
cg24735489	Both
cg16529592	Both
cg04454951	Both
cg24821554	Both
cg11512771	Both
cg11412582	Both
cg09851465	Both
cg22534509	Both
cg26900154	Both
cg05697249	Both
cg17339202	Both
cg08578641	Both
cg02829654	Both
cg18145683	Both
cg08849574	Both

cg02838492	Both
cg11438428	Both
cg11319389	Both
cg24076884	Both
cg11653709	Both
cg13435792	Both
cg07084163	Both
cg06675478	Both
cg24993443	Both
cg16446783	Both
cg13641903	Both
cg12431401	Both
cg23196831	Both
cg21415080	Both
cg24402880	Both
cg03430998	Both
cg15264255	Both
cg27513764	Both
cg12288726	Both
cg09949775	Both
cg02727285	Both
cg21921474	Both
cg09303642	Both
cg21533958	Both
cg12878228	Both
cg07376535	Both
cg03453449	Both
cg01526089	Both
cg20613889	Both
cg08820801	Both
cg08047907	Both
cg27033479	Both
cg18939260	Both
cg15626350	Both
cg27087809	Both
cg18242139	Both
cg11714502	Both
cg23504246	Both
cg01550148	Both
cg08872742	Both
cg11939496	Both
cg23301687	Both
cg01919208	Both
cg23090824	Both
cg10235817	Both
cg16524139	Both
cg10364513	Both
cg18952647	Both
cg08977371	Both
cg21621248	Both
cg24940601	Both
cg13523557	Both
cg23733753	Both

cg21209356	Both
cg24529858	Both
cg23539753	Both
cg01204985	Both
cg05955301	Both
cg07903918	Both
cg04881903	Both
cg22937320	Both
cg08082692	Both
cg05253327	Both
cg04717045	Both
cg01131735	Both
cg08176694	Both
cg05106502	Both
cg27038439	Both
cg14614211	Both
cg26220985	Both
cg21663431	Both
cg01770232	Both
cg20484352	Both
cg21120063	Both
cg14719352	Both
cg16386080	Both
cg21057494	Both
cg14642338	Both
cg23756272	Both
cg17200465	Both
cg08256781	Both
cg04084157	Both
cg09837648	Both
cg08097882	Both
cg22009923	Both
cg18333690	Both
cg12200412	Both
cg12232463	Both
cg05755354	Both
cg09863066	Both
cg05512099	Both
cg09440340	Both
cg24655310	Both
cg06190053	Both
cg06806711	Both
cg26620157	Both
cg23300372	Both
cg00136477	Both
cg21742836	Both
cg00987015	Both
cg23815491	Both
cg19018097	Both
cg09914304	Both
cg14726637	Both
cg17676574	Both
cg25072962	Both

cg13481359	Both
cg15183083	Both
cg02932669	Both
cg01868128	Both
cg19056418	Both
cg00338702	Both
cg02793099	Both
cg15988232	Both
cg20103758	Both
cg25415932	Both
cg19391527	Both
cg27470554	Both
cg15777781	Both
cg08317263	Both
cg05472874	Both
cg21372914	Both
cg22628926	Both
cg19210770	Both
cg07072643	Both
cg18946226	Both
cg01667384	Both
cg02868123	Both
cg15352829	Both
cg14700707	Both
cg20649991	Both
cg26847866	Both
cg13282837	Both
cg08818984	Both
cg12078929	Both
cg26151675	Both
cg05670596	Both
cg05389335	Both
cg04720330	Both
cg06941093	Both
cg22730830	Both
cg05828624	Both
cg11024597	Both
cg19884262	Both
cg27365426	Both
cg05654163	Both
cg24457403	Both
cg26989103	Both
cg22165685	Both
cg23089840	Both
cg06295856	Both
cg21296602	Both
cg18665384	Both
cg09902130	Both
cg22492020	Both
cg21092324	Both
cg11695684	Both
cg17453778	Both
cg20831708	Both

cg23896545	Both
cg27070899	Both
cg05719902	Both
cg06714705	Both
cg18621299	Both
cg15046693	Both
cg19713196	Both
cg09243021	Both
cg19241744	Both
cg16927606	Both
cg19418515	Both
cg26372517	Both
cg00436603	Both
cg17233601	Both
cg08460026	Both
cg18515587	Both
cg25411725	Both
cg03375833	Both
cg11484872	Both
cg20890210	Both
cg15552238	Both
cg04078896	Both
cg08101264	Both
cg20339230	Both
cg03567830	Both
cg08124030	Both
cg14260643	Both
cg03991512	Both
cg14824983	Both
cg20674521	Both
cg15640375	Both
cg05358404	Both
cg27081230	Both
cg12682367	Both
cg14534464	Both
cg17317962	Both
cg24587268	Both
cg20792833	Both
cg23317501	Both
cg26896762	Both
cg07349094	Both
cg10246520	Both
cg20795913	Both
cg18482268	Both
cg14611112	Both
cg00627233	Both
cg01993576	Both
cg15742700	Both
cg05596756	Both
cg13311440	Both
cg07657236	Both
cg18437633	Both
cg16570917	Both

cg01623438	Both
cg18627308	Both
cg23663653	Both
cg20804821	Both
cg19403023	Both
cg11733245	Both
cg08614201	Both
cg18176712	Both
cg20339650	Both
cg04999691	Both
cg18105675	Both
cg03283569	Both
cg11265941	Both
cg04103514	Both
cg16608652	Both
cg27063986	Both
cg04783231	Both
cg15996947	Both
cg20903926	Both
cg10590292	Both
cg06545504	Both
cg25464840	Both
cg14792480	Both
cg15195412	Both
cg12965512	Both
cg14264994	Both
cg19297232	Both
cg15875314	Both
cg19461344	Both
cg03425110	Both
cg26575445	Both
cg06096336	Both
cg22518733	Both
cg11804789	Both
cg20764656	Both
cg04488521	Both
cg09134726	Both
cg04353769	Both
cg04726200	Both
cg09432154	Both
cg26256793	Both
cg10300684	Both
cg25803583	Both
cg06646021	Both
cg02905245	Both
cg10125195	Both
cg08458487	Both
cg03882305	Both
cg26404725	Both
cg11979312	Both
cg20967028	Both
cg27316956	Both
cg26207503	Both

cg20523861	Both
cg12914657	Both
cg03127334	Both
cg07637239	Both
cg03874199	Both
cg17412258	Both
cg16604516	Both
cg11206634	Both
cg21517055	Both
cg14511156	Both
cg15642326	Both
cg24800810	Both
cg08815403	Both
cg22396129	Both
cg18397523	Both
cg00516481	Both
cg09893305	Both
cg06268694	Both
cg10608341	Both
cg16139316	Both
cg24620905	Both
cg23326689	Both
cg24408313	Both
cg11403598	Both
cg26862286	Both
cg02748539	Both
cg11428724	Both
cg25608041	Both
cg03909500	Both
cg22022041	Both
cg14444710	Both
cg06398181	Both
cg02196805	Both
cg26239233	Both
cg01481976	Both
cg00213044	Both
cg11538128	Both
cg14329157	Both
cg14546153	Both
cg20437604	Both
cg01017147	Both
cg12413566	Both
cg12489960	Both
cg06908778	Both
cg13573276	Both
cg03099771	Both
cg23673107	Both
cg26525091	Both
cg15156836	Both
cg06244417	Both
cg21591452	Both
cg12781568	Both
cg25300386	Both

cg09099744	Both
cg12855851	Both
cg09106999	Both
cg19723473	Both
cg25427638	Both
cg15853125	Both
cg09426307	Both
cg07159490	Both
cg15703512	Both
cg15967525	Both
cg00775197	Both
cg26556134	Both
cg12265829	Both
cg08351331	Both
cg01770400	Both
cg26062856	Both
cg04498511	Both
cg21604042	Both
cg12847373	Both
cg25426743	Both
cg06244906	Both
cg06899976	Both
cg03918304	Both
cg21171615	Both
cg23093496	Both
cg10887021	Both
cg22527345	Both
cg20189937	Both
cg18223379	Both
cg18959422	Both
cg09492887	Both
cg25463779	Both
cg20444256	Both
cg21660130	Both
cg01510051	Both
cg23349242	Both
cg08987989	Both
cg02537838	Both
cg09011597	Both
cg00902195	Both
cg08260959	Both
cg16792160	Both
cg17607231	Both
cg25268718	Both
cg09614401	Both
cg00842351	Both
cg11154542	Both
cg04870470	Both
cg17826679	Both
cg00757952	Both
cg21688264	Both
cg09610963	Both
cg00619207	Both

cg04349727	Both
cg10126923	Both
cg00013618	Both
cg26984805	Both
cg01618660	Both
cg11308840	Both
cg10044101	Both
cg24512973	Both
cg18638581	Both
cg06454226	Both
cg07073964	Both
cg15447486	Both
cg21312148	Both
cg11465372	Both
cg04922810	Both
cg21164303	Both
cg27043873	Both
cg15489294	Both
cg24262376	Both
cg01589580	Both
cg14654385	Both
cg03621001	Both
cg09882647	Both
cg16192575	Both
cg14681055	Both
cg11075745	Both
cg21176048	Both
cg11469321	Both
cg05976074	Both
cg00449941	Both
cg04786857	Both
cg18384097	Both
cg16842214	Both
cg02337447	Both
cg04259560	Both
cg14188111	Both
cg21238818	Both
cg10636246	Both
cg07136421	Both
cg23843812	Both
cg21674595	Both
cg17589341	Both
cg24447890	Both
cg16983159	Both
cg25995212	Both
cg26599006	Both
cg16377880	Both
cg07379574	Both
cg11560645	Both
cg12508624	Both
cg07403255	Both
cg14015044	Both
cg01449591	Both

cg05824215	Both
cg23749046	Both
cg08611714	Both
cg00335286	Both
cg07485588	Both
cg22183706	Both
cg12998491	Both
cg17940013	Both
cg15485859	Both
cg07730329	Both
cg25983380	Both
cg03886110	Both
cg10832945	Both
cg12456510	Both
cg14324838	Both
cg01344452	Both
cg26365553	Both
cg00564163	Both
cg18669588	Both
cg06793062	Both
cg23244913	Both
cg00094319	Both
cg19868691	Both
cg19664945	Both
cg08290628	Both
cg15543551	Both
cg09440243	Both
cg15042080	Both
cg06572160	Both
cg20507276	Both
cg23355492	Both
cg04623837	Both
cg21696393	Both
cg05483509	Both
cg16731240	Both
cg01805282	Both
cg22289810	Both
cg09837977	Both
cg24092253	Both
cg02774439	Both
cg06759890	Both
cg08448751	Both
cg07728874	Both
cg23003832	Both
cg08946117	Both
cg19814116	Both
cg23694248	Both
cg01081263	Both
cg18552413	Both
cg23054883	Both
cg26780333	Both
cg25167447	Both
cg04721098	Both

cg22580512	Both
cg04891836	Both
cg07403350	Both
cg19067730	Both
cg17830308	Both
cg10763288	Both
cg01107031	Both
cg23983449	Both
cg08510456	Both
cg15046675	Both
cg13449778	Both
cg24433189	Both
cg18335068	Both
cg13795840	Both
cg19690404	Both
cg21004129	Both
cg20535781	Both
cg05130485	Both
cg06403553	Both
cg00767581	Both
cg15057726	Both
cg01870826	Both
cg18877506	Both
cg20584011	Both
cg16112945	Both
cg07326586	Both
cg13943564	Both
cg06541857	Both
cg12311132	Both
cg02311163	Both
cg24868271	Both
cg06041363	Both
cg12793610	Both
cg09563216	Both
cg25465406	Both
cg20485165	Both
cg10978355	Both
cg24092914	Both
cg18573383	Both
cg19528976	Both
cg10757144	Both
cg14737977	Both
cg08463485	Both
cg23612220	Both
cg20074593	Both
cg26099316	Both
cg17820828	Both
cg01040850	Both
cg05365670	Both
cg26920757	Both
cg19988449	Both
cg12271671	Both
cg15374234	Both

cg04375683	Both
cg07685034	Both
cg06711560	Both
cg22472290	Both
cg26111757	Both
cg15491567	Both
cg15014458	Both
cg02148642	Both
cg20977864	Both
cg17786776	Both
cg19306866	Both
cg18807515	Both
cg04878152	Both
cg16415058	Both
cg00931491	Both
cg14844130	Both
cg00918005	Both
cg04330449	Both
cg13991233	Both
cg16363586	Both
cg26570233	Both
cg26393983	Both
cg18602919	Both
cg07826255	Both
cg21226224	Both
cg19718882	Both
cg27532722	Both
cg18878432	Both
cg27342801	Both
cg01546563	Both
cg01580044	Both
cg18938204	Both
cg09416313	Both
cg07331806	Both
cg18790143	Both
cg17465304	Both
cg00466436	Both
cg09671611	Both
cg13348944	Both
cg10849854	Both
cg20023231	Both
cg01361777	Both
cg06496654	Both
cg16516400	Both
cg14717170	Both
cg08763351	Both
cg21022247	Both
cg02849695	Both
cg14375111	Both
cg01152019	Both
cg02671171	Both
cg21291985	Both
cg10647513	Both

cg18182399	Both
cg00096922	Both
cg10362591	Both
cg26644395	Both
cg00430287	Both
cg23613317	Both
cg26738880	Both
cg17142149	Both
cg10709021	Both
cg08587542	Both
cg24751129	Both
cg26164184	Both
cg22131691	Both
cg25027501	Both
cg01402255	Both
cg15337006	Both
cg05208878	Both
cg08727202	Both
cg27449489	Both
cg10098541	Both
cg27320127	Both
cg24674703	Both
cg02388150	Both
cg03686067	Both
cg20264732	Both
cg05485060	Both
cg00916635	Both
cg12941369	Both
cg11481351	Both
cg23642747	Both
cg16831889	Both
cg16272420	Both
cg26721264	Both
cg24507762	Both
cg10305797	Both
cg03266453	Both
cg00563932	Both
cg19248557	Both
cg26154999	Both
cg24210717	Both
cg06533629	Both
cg02537023	Both
cg00690280	Both
cg04300115	Both
cg07643942	Both
cg23185262	Both
cg11584690	Both
cg16462075	Both
cg26450866	Both
cg09971811	Both
cg15691199	Both
cg02545192	Both
cg25725843	Both

cg05659526	Both
cg16076328	Both
cg17699374	Both
cg25778479	Both
cg23850212	Both
cg15105703	Both
cg09350141	Both
cg18979223	Both
cg19831575	Both
cg03852570	Both
cg08285151	Both
cg25182523	Both
cg14387505	Both
cg06501790	Both
cg01558777	Both
cg20747455	Both
cg06692050	Both
cg18738906	Both
cg19462352	Both
cg07841014	Both
cg08831594	Both
cg25006823	Both
cg16361890	Both
cg17536532	Both
cg04523589	Both
cg08555657	Both
cg16303562	Both
cg14479329	Both
cg08460435	Both
cg20520888	Both
cg16521028	Both
cg04962134	Both
cg22858288	Both
cg23699324	Both
cg24237576	Both
cg18611245	Both
cg22984277	Both
cg16636110	Both
cg24272907	Both
cg03375002	Both
cg07601320	Both
cg25750259	Both
cg18598959	Both
cg16601385	Both
cg21960110	Both
cg22511262	Both
cg03022541	Both
cg07034561	Both
cg13398291	Both
cg16536918	Both
cg05037688	Both
cg02589695	Both
cg15589354	Both

cg20542800	Both
cg02787991	Both
cg11161417	Both
cg21039631	Both
cg02774160	Both
cg05671350	Both
cg18854666	Both
cg09188980	Both
cg14974772	Both
cg12089698	Both
cg13547644	Both
cg22268164	Both
cg01919488	Both
cg01124420	Both
cg07017374	Both
cg06943865	Both
cg25494064	Both
cg17204557	Both
cg15343119	Both
cg04663194	Both
cg15484384	Both
cg03600318	Both
cg14312526	Both
cg07260592	Both
cg00187686	Both
cg12936220	Both
cg23040064	Both
cg27501458	Both
cg11267879	Both
cg12045829	Both
cg18174542	Both
cg04176254	Both
cg22815534	Both
cg16463460	Both
cg24768561	Both
cg20209009	Both
cg18668753	Both
cg14399060	Both
cg23964386	Both
cg24176563	Both
cg03569637	Both
cg14047008	Both
cg14544583	Both
cg23988567	Both
cg04405541	Both
cg01813965	Both
cg09427311	Both
cg04111761	Both
cg14116122	Both
cg27352992	Both
cg14237152	Both
cg18241647	Both
cg13344740	Both

cg12833011	Both
cg13614181	Both
cg15660498	Both
cg24964368	Both
cg17191715	Both
cg04739485	Both
cg01369413	Both
cg02282237	Both
cg18137704	Both
cg15789095	Both
cg15743985	Both
cg14297029	Both
cg05663262	Both
cg13297865	Both
cg24396745	Both
cg15175266	Both
cg17560332	Both
cg09945801	Both
cg12491659	Both
cg13354523	Both
cg10929387	Both
cg18839416	Both
cg15258980	Both
cg19435264	Both
cg11868900	Both
cg13560436	Both
cg08752459	Both
cg18705067	Both
cg20972214	Both
cg12312863	Both
cg10883621	Both
cg20070090	Both
cg01009664	Both
cg18135555	Both
cg24860534	Both
cg08390021	Both
cg00858899	Both
cg07947930	Both
cg07007400	Both
cg08319238	Both
cg17961200	Both
cg00183916	Both
cg09674215	Both
cg27180443	Both
cg07218880	Both
cg25302419	Both
cg23904249	Both
cg05261299	Both
cg09619786	Both
cg07434278	Both
cg16504798	Both
cg12100791	Both
cg10861599	Both

cg08535373	Both
cg05512756	Both
cg12433691	Both
cg05715649	Both
cg08861115	Both
cg24612198	Both
cg06324671	Both
cg04511195	Both
cg12000587	Both
cg09039163	Both
cg13246592	Both
cg20981615	Both
cg05726109	Both
cg15156367	Both
cg08876932	Both
cg20179697	Both
cg18555117	Both
cg15149098	Both
cg23973000	Both
cg08292050	Both
cg09243900	Both
cg23323671	Both
cg06958537	Both
cg23668631	Both
cg26220350	Both
cg09837943	Both
cg10146929	Both
cg16158807	Both
cg14458615	Both
cg05899618	Both
cg23771929	Both
cg02927346	Both
cg21152671	Both
cg00407150	Both
cg07506795	Both
cg12832565	Both
cg04797496	Both
cg14973995	Both
cg02990612	Both
cg02431687	Both
cg23440155	Both
cg06293195	Both
cg21547708	Both
cg20588069	Both
cg04458548	Both
cg09918674	Both
cg22377963	Both
cg12799895	Both
cg10316764	Both
cg26674132	Both
cg13084335	Both
cg22165175	Both
cg24167037	Both

cg25509184	Both
cg27420123	Both
cg09025324	Both
cg14380517	Both
cg07644368	Both
cg22836470	Both
cg04884908	Both
cg11021744	Both
cg16530429	Both
cg19764436	Both
cg26202340	Both
cg13059782	Both
cg04126866	Both
cg22241124	Both
cg17791651	Both
cg11251877	Both
cg01584473	Both
cg23207990	Both
cg06585893	Both
cg02656594	Both
cg02503850	Both
cg26465611	Both
cg02284188	Both
cg14419187	Both
cg05248470	Both
cg25938646	Both
cg01580681	Both
cg05556020	Both
cg23182299	Both
cg15060813	Both
cg21491308	Both
cg19135761	Both
cg21359747	Both
cg04014889	Both
cg10624445	Both
cg24765079	Both
cg24723331	Both
cg15786837	Both
cg13727946	Both
cg18722841	Both
cg15765212	Both
cg20256494	Both
cg02105856	Both
cg01443452	Both
cg13468685	Both
cg07327468	Both
cg12973651	Both
cg12629325	Both
cg10098888	Both
cg19630689	Both
cg08399444	Both
cg00243313	Both
cg25671438	Both

cg11016563	Both
cg08700306	Both
cg20247048	Both
cg17183546	Both
cg06186808	Both
cg03885639	Both
cg14145194	Both
cg06725035	Both
cg05810550	Both
cg20789824	Both
cg16708623	Both
cg03818682	Both
cg06817264	Both
cg06917325	Both
cg14494596	Both
cg05501357	Both
cg24489015	Both
cg19965810	Both
cg00292662	Both
cg06650786	Both
cg21578541	Both
cg00925717	Both
cg20395892	Both
cg14182690	Both
cg19895197	Both
cg24421410	Both
cg12150401	Both
cg17408686	Both
cg04586023	Both
cg12869058	Both
cg01646665	Both
cg03064067	Both
cg05517711	Both
cg03616357	Both
cg24838010	Both
cg14236389	Both
cg03389133	Both
cg01454134	Both
cg03977657	Both
cg16466334	Both
cg19042947	Both
cg25004981	Both
cg21269934	Both
cg23471482	Both
cg07374637	Both
cg22941086	Both
cg10466917	Both
cg20025656	Both
cg05246522	Both
cg07719512	Both
cg21505886	Both
cg07717632	Both
cg26536164	Both

cg23663476	Both
cg10298815	Both
cg13042487	Both
cg25993718	Both
cg13265789	Both
cg03860400	Both
cg18804206	Both
cg13168820	Both
cg15539420	Both
cg18919097	Both
cg21601405	Both
cg10533434	Both
cg24939733	Both
cg12300353	Both
cg07028533	Both
cg03473294	Both
cg01936555	Both
cg14385738	Both
cg19883905	Both
cg24252809	Both
cg19125999	Both
cg01917648	Both
cg09952204	Both
cg19779211	Both
cg00443307	Both
cg04336379	Both
cg05697976	Both
cg13739417	Both
cg14333454	Both
cg24290574	Both
cg26516759	Both
cg19923326	Both
cg05187322	Both
cg09997082	Both
cg20881054	Both
cg00112517	Both
cg11554605	Both
cg00919857	Both
cg19759064	Both
cg05154390	Both
cg15627025	Both
cg19046959	Both
cg14430151	Both
cg11959435	Both
cg15791248	Both
cg23894003	Both
cg09314766	Both
cg19258882	Both
cg27508071	Both
cg18931815	Both
cg15475323	Both
cg22222251	Both
cg22436411	Both

cg24879335	Both
cg06319346	Both
cg27345534	Both
cg23855093	Both
cg22461835	Both
cg12343082	Both
cg02298612	Both
cg23950724	Both
cg16008138	Both
cg16689634	Both
cg22951794	Both
cg03891191	Both
cg10322876	Both
cg11500797	Both
cg21243096	Both
cg16014085	Both
cg18220560	Both
cg15966757	Both
cg05250458	Both
cg14614901	Both
cg00804392	Both
cg23033845	Both
cg13652336	Both
cg02275294	Both
cg21458907	Both
cg24110050	Both
cg22141781	Both
cg08818385	Both
cg15320474	Both
cg08766149	Both
cg18151487	Both
cg17301902	Both
cg14415034	Both
cg27299588	Both
cg24678320	Both
cg07634191	Both
cg21991396	Both
cg14898892	Both
cg21576698	Both
cg17484237	Both
cg07447922	Both
cg04719766	Both
cg15344028	Both
cg01774645	Both
cg11762346	Both
cg19885761	Both
cg10059959	Both
cg21130374	Both
cg14654731	Both
cg16512727	Both
cg09511421	Both
cg03032025	Both
cg26043257	Both

cg25061755	Both
cg00290506	Both
cg02947253	Both
cg23579062	Both
cg13801381	Both
cg15422147	Both
cg07125166	Both
cg18180155	Both
cg03017653	Both
cg01320507	Both
cg21115990	Both
cg22253945	Both
cg05064181	Both
cg09878269	Both
cg25177139	Both
cg00729875	Both
cg23910243	Both
cg08935003	Both
cg23799313	Both
cg09458237	Both
cg04797323	Both
cg09475757	Both
cg04636557	Both
cg15536230	Both
cg01512854	Both
cg01214209	Both
cg22723026	Both
cg23418591	Both
cg14754581	Both
cg03160508	Both
cg22161476	Both
cg07447773	Both
cg25098401	Both
cg11896923	Both
cg22039846	Both
cg19836199	Both
cg14975238	Both
cg01172899	Both
cg10328573	Both
cg15840985	Both
cg22598028	Both
cg23239396	Both
cg27285599	Both
cg18354594	Both
cg04587829	Both
cg23113963	Both
cg10562586	Both
cg01013324	Both
cg02222362	Both
cg27476329	Both
cg21706946	Both
cg12499211	Both
cg07935568	Both

cg18349835	Both
cg21096966	Both
cg12953249	Both
cg25341032	Both
cg00211661	Both
cg01664666	Both
cg06377278	Both
cg00347904	Both
cg02681442	Both
cg24532476	Both
cg22443330	Both
cg27159719	Both
cg15916061	Both
cg22918700	Both
cg00666746	Both
cg19438514	Both
cg15113803	Both
cg02903525	Both
cg03504078	Both
cg21461100	Both
cg24298280	Both
cg14191360	Both
cg23506842	Both
cg03158400	Both
cg26021627	Both
cg18838701	Both
cg13062935	Both
cg01313514	Both
cg21686987	Both
cg19111262	Both
cg15310162	Both
cg24662718	Both
cg18344745	Both
cg17936488	Both
cg16150435	Both
cg10539507	Both
cg11283860	Both
cg01781266	Both
cg25057743	Both
cg02275359	Both
cg02149446	Both
cg22780475	Both
cg22029275	Both
cg03112869	Both
cg10161121	Both
cg03381111	Both
cg11285843	Both
cg24322623	Both
cg09801065	Both
cg13897627	Both
cg18533225	Both
cg00401678	Both
cg13608094	Both

cg00548060	Both
cg16509569	Both
cg02968557	Both
cg10878307	Both
cg25780543	Both
cg25112191	Both
cg26312920	Both
cg21142272	Both
cg23654219	Both
cg14165663	Both
cg11521965	Both
cg17714799	Both
cg01144251	Both
cg17729941	Both
cg13797282	Both
cg16421285	Both
cg19447966	Both
cg18901980	Both
cg13279585	Both
cg20001829	Both
cg01939681	Both
cg06710648	Both
cg21604856	Both
cg04156850	Both
cg13424229	Both
cg26222045	Both
cg06055013	Both
cg13083810	Both
cg11759378	Both
cg09624565	Both
cg07942995	Both
cg07851192	Both
cg14662756	Both
cg24010336	Both
cg19620294	Both
cg02813121	Both
cg24956866	Both
cg27286999	Both
cg22407458	Both
cg20334738	Both
cg09874776	Both
cg03732056	Both
cg19110684	Both
cg18854045	Both
cg06468695	Both
cg14918082	Both
cg06639544	Both
cg27625732	Both
cg08747377	Both
cg16848873	Both
cg21033855	Both
cg25119415	Both
cg10281002	Both

cg07102705	Both
cg11397854	Both
cg26374101	Both
cg25050026	Both
cg07755653	Both
cg27329371	Both
cg15843823	Both
cg14601284	Both
cg06501070	Both
cg23580000	Both
cg16112129	Both
cg17177699	Both
cg04072323	Both
cg09044743	Both
cg20239740	Both
cg12564962	Both
cg18146152	Both
cg27496506	Both
cg04754011	Both
cg01668126	Both
cg21959619	Both
cg13722123	Both
cg06327515	Both
cg03561565	Both
cg25256723	Both
cg13587552	Both
cg25894551	Both
cg13391235	Both
cg03238797	Both
cg03734874	Both
cg22220722	Both
cg06270401	Both
cg16483466	Both
cg20512303	Both
cg05949660	Both
cg07773116	Both
cg24454143	Both
cg19884658	Both
cg14223444	Both
cg08145177	Both
cg11532513	Both
cg01573562	Both
cg25784308	Both
cg13097816	Both
cg25919221	Both
cg13323752	Both
cg13255190	Both
cg04564030	Both
cg02724472	Both
cg04481779	Both
cg07181881	Both
cg13079099	Both
cg19404582	Both

cg24446548	Both
cg21122774	Both
cg06194186	Both
cg01471713	Both
cg18267374	Both
cg25494227	Both
cg00888479	Both
cg19591881	Both
cg00463202	Both
cg17651821	Both
cg25500444	Both
cg03311899	Both
cg10315334	Both
cg25645462	Both
cg19317715	Both
cg17264618	Both
cg12281657	Both
cg22334665	Both
cg25027167	Both
cg15774153	Both
cg16864658	Both
cg03889226	Both
cg15783800	Both
cg17425616	Both
cg16742703	Both
cg12266049	Both
cg13577076	Both
cg03634234	Both
cg26850754	Both
cg23486067	Both
cg01366419	Both
cg15868302	Both
cg17251713	Both
cg15754084	Both
cg06911084	Both
cg03843951	Both
cg19740375	Both
cg03742003	Both
cg04278702	Both
cg18236734	Both
cg15528736	Both
cg21044104	Both
cg26368842	Both
cg01049530	Both
cg09573795	Both
cg13271951	Both
cg06224510	Both
cg23307338	Both
cg19378133	Both
cg10140638	Both
cg10889070	Both
cg26838900	Both
cg15457899	Both

cg08251399	Both
cg10037068	Both
cg24194539	Both
cg02293044	Both
cg24715735	Both
cg05444024	Both
cg25211252	Both
cg03852144	Both
cg03743584	Both
cg25623459	Both
cg15554401	Both
cg13507429	Both
cg13921352	Both
cg03046445	Both
cg18542098	Both
cg10412312	Both
cg02082571	Both
cg15837252	Both
cg25712380	Both
cg13993218	Both
cg25895922	Both
cg04759756	Both
cg03630088	Both
cg10203483	Both
cg13960192	Both
cg19258973	Both
cg16418329	Both
cg17628717	Both
cg00498305	Both
cg16899442	Both
cg03775422	Both
cg04021421	Both
cg13958426	Both
cg20848130	Both
cg11058932	Both
cg15210427	Both
cg04887278	Both
cg04983977	Both
cg07013734	Both
cg15202954	Both
cg26728422	Both
cg27631256	Both
cg19238840	Both
cg26317111	Both
cg26024843	Both
cg21902544	Both
cg02254407	Both
cg03894103	Both
cg22411207	Both
cg25994725	Both
cg04351979	Both
cg04645843	Both
cg03898365	Both

cg00152644	Both
cg02701137	Both
cg24765446	Both
cg12866859	Both
cg27096144	Both
cg10321723	Both
cg00929606	Both
cg19593572	Both
cg13984181	Both
cg03914397	Both
cg21500966	Both
cg03379131	Both
cg11459714	Both
cg04879235	Both
cg20053158	Both
cg06432655	Both
cg05362516	Both
cg10872209	Both
cg10409560	Both
cg09001777	Both
cg13315690	Both
cg25882366	Both
cg03165378	Both
cg07536847	Both
cg25327013	Both
cg12841020	Both
cg22670733	Both
cg12420104	Both
cg27319898	Both
cg01669948	Both
cg10995380	Both
cg24475171	Both
cg06405206	Both
cg08965235	Both
cg22660578	Both
cg13125510	Both
cg02229946	Both
cg13697378	Both
cg19504245	Both
cg20345978	Both
cg09396217	Both
cg08623787	Both
cg02046017	Both
cg16933922	Both
cg10318258	Both
cg27158867	Both
cg15760840	Both
cg17237813	Both
cg24680602	Both
cg01963696	Both
cg18432384	Both
cg16474684	Both
cg09381003	Both

cg01934790	Both
cg02921257	Both
cg27456885	Both
cg01145396	Both
cg14396117	Both
cg22572159	Both
cg08519905	Both
cg11719297	Both
cg10742801	Both
cg25813714	Both
cg25971347	Both
cg04008913	Both
cg24109894	Both
cg00226923	Both
cg13126790	Both
cg14813083	Both
cg22778981	Both
cg00995152	Both
cg06037693	Both
cg17178888	Both
cg20851097	Both
cg08418111	Both
cg10261191	Both
cg26571739	Both
cg02981853	Both
cg21184174	Both
cg18085517	Both
cg01693350	Both
cg08918749	Both
cg13954572	Both
cg26940261	Both
cg04616963	Both
cg00325491	Both
cg04263186	Both
cg20909686	Both
cg03240509	Both
cg16263182	Both
cg04086834	Both
cg03640944	Both
cg02654291	Both
cg11099899	Both
cg18511007	Both
cg02936740	Both
cg25226014	Both
cg23128056	Both
cg08007665	Both
cg25370441	Both
cg03005261	Both
cg24063382	Both
cg10084993	Both
cg20879768	Both
cg20804101	Both
cg02847216	Both

cg19910382	Both
cg16612562	Both
cg03814826	Both
cg17029168	Both
cg02224164	Both
cg24715245	Both
cg14911395	Both
cg05396987	Both
cg05615150	Both
cg17709873	Both
cg24730207	Both
cg26918728	Both
cg15814508	Both
cg11990309	Both
cg10683939	Both
cg02540157	Both
cg05135288	Both
cg04460372	Both
cg14761227	Both
cg00949442	Both
cg10057065	Both
cg13526007	Both
cg10212621	Both
cg22705929	Both
cg02431964	Both
cg12554857	Both
cg17509612	Both
cg11880010	Both
cg08260891	Both
cg17788013	Both
cg04655481	Both
cg01543654	Both
cg02602411	Both
cg27401095	Both
cg14176836	Both
cg17483510	Both
cg11516377	Both
cg22621695	Both
cg09970593	Both
cg24929834	Both
cg12818699	Both
cg05190718	Both
cg15584813	Both
cg24194132	Both
cg09516965	Both
cg00554250	Both
cg22954265	Both
cg07732037	Both
cg26632776	Both
cg12741420	Both
cg21280510	Both
cg10693071	Both
cg04713521	Both

cg24851490	Both
cg12916723	Both
cg13401339	Both
cg20126106	Both
cg05241571	Both
cg09649610	Both
cg07906724	Both
cg06882877	Both
cg10235741	Both
cg02374486	Both
cg18374517	Both
cg15873301	Both
cg23614979	Both
cg02043070	Both
cg24841244	Both
cg20743744	Both
cg25077752	Both
cg21672276	Both
cg19427610	Both
cg16772514	Both
cg25875213	Both
cg15979932	Both
cg12271587	Both
cg23239828	Both
cg01617750	Both
cg01171858	Both
cg26189983	Both
cg17342283	Both
cg15648905	Both
cg05666713	Both
cg15895197	Both
cg22172494	Both
cg26866325	Both
cg21453309	Both
cg01853981	Both
cg14153740	Both
cg26349773	Both
cg06993413	Both
cg01322134	Both
cg15361590	Both
cg15835825	Both
cg04545516	Both
cg10642330	Both
cg20080624	Both
cg19526600	Both
cg04968473	Both
cg04304130	Both
cg18008766	Both
cg20950011	Both
cg00915206	Both
cg18438777	Both
cg13603171	Both
cg00263760	Both

cg12614105	Both
cg11191210	Both
cg12164282	Both
cg05348870	Both
cg03414321	Both
cg00638514	Both
cg01278291	Both
cg15147516	Both
cg20404387	Both
cg24650501	Both
cg18294257	Both
cg03776060	Both
cg04631202	Both
cg17387870	Both
cg26626089	Both
cg11822932	Both
cg11825652	Both
cg10236239	Both
cg01739167	Both
cg15559700	Both
cg22143352	Both
cg25193494	Both
cg07749074	Both
cg09601629	Both
cg10207745	Both
cg14037665	Both
cg05040360	Both
cg05740244	Both
cg26624914	Both
cg19712821	Both
cg17945001	Both
cg21754343	Both
cg13158571	Both
cg08899626	Both
cg13273136	Both
cg04810997	Both
cg10051054	Both
cg09155001	Both
cg07770222	Both
cg14366490	Both
cg21993406	Both
cg25918245	Both
cg01424107	Both
cg15983538	Both
cg17929627	Both
cg10997248	Both
cg16077929	Both
cg00476577	Both
cg18917378	Both
cg22227965	Both
cg20300246	Both
cg01532103	Both
cg18286698	Both

cg01348086	Both
cg22346765	Both
cg11051139	Both
cg14605021	Both
cg15379858	Both
cg05661838	Both
cg16094954	Both
cg09217923	Both
cg03440267	Both
cg16426459	Both
cg00903242	Both
cg01025842	Both
cg23391785	Both
cg17071957	Both
cg18660898	Both
cg13689073	Both
cg17296078	Both
cg19697981	Both
cg26394380	Both
cg06206626	Both
cg08074477	Both
cg11286122	Both
cg02217814	Both
cg12728032	Both
cg18089852	Both
cg17356252	Both
cg05624932	Both
cg24818434	Both
cg16140179	Both
cg01566404	Both
cg25433648	Both
cg09450238	Both
cg18201198	Both
cg02017155	Both
cg16894211	Both
cg08398233	Both
cg21435336	Both
cg08284151	Both
cg22678136	Both
cg24194775	Both
cg23114594	Both
cg11429658	Both
cg24858591	Both
cg23580945	Both
cg12864853	Both
cg10861751	Both
cg18219226	Both
cg21614638	Both
cg10501629	Both
cg26963271	Both
cg10848367	Both
cg26066361	Both
cg10691387	Both

cg09584711	Both
cg26385222	Both
cg09457245	Both
cg13163729	Both
cg04454050	Both
cg19342782	Both
cg24274272	Both
cg15607672	Both
cg09998591	Both
cg21331821	Both
cg20442697	Both
cg11750883	Both
cg00504595	Both
cg22495124	Both
cg12699145	Both
cg03128832	Both
cg18272264	Both
cg00654816	Both
cg04527363	Both
cg09829319	Both
cg17854440	Both
cg03287877	Both
cg05473677	Both
cg27182761	Both
cg12032049	Both
cg13755070	Both
cg22325703	Both
cg06747888	Both
cg24747122	Both
cg13645811	Both
cg14850026	Both
cg23877385	Both
cg03285457	Both
cg04784672	Both
cg07310661	Both
cg23455517	Both
cg00091693	Both
cg24256211	Both
cg00943245	Both
cg04958703	Both
cg14012294	Both
cg08586737	Both
cg19252956	Both
cg18540325	Both
cg08722122	Both
cg26705553	Both
cg13928306	Both
cg12645220	Both
cg12968903	Both
cg12167564	Both
cg20925955	Both
cg20178764	Both
cg11822964	Both

cg19731268	Both
cg03087937	Both
cg14600885	Both
cg08880153	Both
cg10604333	Both
cg07651242	Both
cg07558455	Both
cg10520887	Both
cg09009111	Both
cg04398282	Both
cg04245402	Both
cg13019092	Both
cg22975913	Both
cg01761409	Both
cg03886898	Both
cg23780947	Both
cg13797031	Both
cg05184394	Both
cg22881914	Both
cg23978322	Both
cg26466858	Both
cg11126134	Both
cg00511475	Both
cg16799087	Both
cg27622610	Both
cg12213910	Both
cg05516537	Both
cg15250797	Both
cg24545967	Both
cg13084525	Both
cg17067993	Both
cg16990174	Both
cg08303146	Both
cg12041387	Both
cg00806490	Both
cg00689340	Both
cg21627181	Both
cg00334507	Both
cg08614481	Both
cg21245372	Both
cg12876594	Both
cg06637774	Both
cg02854090	Both
cg01193293	Both
cg13445249	Both
cg11195002	Both
cg15207619	Both
cg20904010	Both
cg14944362	Both
cg22215192	Both
cg00629217	Both
cg27257408	Both
cg13906813	Both

cg17399166	Both
cg26014197	Both
cg14722162	Both
cg01757745	Both
cg04424621	Both
cg17918501	Both
cg12813792	Both
cg12340144	Both
cg13176979	Both
cg16787352	Both
cg04007936	Both
cg13565215	Both
cg18661868	Both
cg05000446	Both
cg17740645	Both
cg10801369	Both
cg18552939	Both
cg15787039	Both
cg11634198	Both
cg21494776	Both
cg08603768	Both
cg21929875	Both
cg15739437	Both
cg04001668	Both
cg08684473	Both
cg02347487	Both
cg17371081	Both
cg12688670	Both
cg22354595	Both
cg12380854	Both
cg06126510	Both
cg08040471	Both
cg18328334	Both
cg11673803	Both
cg21063899	Both
cg17026542	Both
cg27257987	Both
cg02497758	Both
cg03801286	Both
cg14732540	Both
cg02506908	Both
cg07441143	Both
cg09706243	Both
cg04001802	Both
cg23042138	Both
cg00625653	Both
cg19160878	Both
cg09254939	Both
cg21926138	Both
cg04391111	Both
cg25229305	Both
cg21831174	Both
cg16184943	Both

cg04747619	Both
cg00830029	Both
cg21958798	Both
cg07185695	Both
cg05559445	Both
cg11631518	Both
cg12791465	Both
cg13926569	Both
cg17518825	Both
cg03724882	Both
cg00613255	Both
cg13206017	Both
cg01260219	Both
cg22392666	Both
cg13307384	Both
cg13549845	Both
cg05836145	Both
cg24122922	Both
cg23211240	Both
cg08422599	Both
cg04993257	Both
cg20537629	Both
cg20329958	Both
cg18053607	Both
cg22461018	Both
cg04612566	Both
cg23326301	Both
cg27560864	Both
cg00168942	Both
cg16191009	Both
cg16335762	Both
cg24745738	Both
cg10834677	Both
cg20503329	Both
cg20305726	Both
cg14009688	Both
cg26789453	Both
cg24453664	Both
cg00297584	Both
cg13139843	Both
cg03103192	Both
cg22805308	Both
cg22960952	Both
cg05193832	Both
cg17791936	Both
cg20588045	Both
cg10545682	Both
cg12385643	Both
cg10599444	Both
cg11200929	Both
cg21965997	Both
cg13150596	Both
cg10037005	Both

cg02104644	Both
cg11269533	Both
cg26219051	Both
cg16204289	Both
cg17189494	Both
cg23248452	Both
cg02867079	Both
cg22373112	Both
cg07285167	Both
cg10421192	Both
cg13982505	Both
cg24215443	Both
cg20870559	Both
cg19948393	Both
cg04317399	Both
cg23771661	Both
cg25766774	Both
cg05221264	Both
cg05970437	Both
cg26918645	Both
cg26729026	Both
cg03504701	Both
cg07693270	Both
cg02046340	Both
cg17978274	Both
cg19850406	Both
cg19946699	Both
cg22988566	Both
cg01708964	Both
cg20083730	Both
cg13446622	Both
cg06918467	Both
cg07126559	Both
cg11235426	Both
cg18636558	Both
cg19573166	Both
cg07711097	Both
cg23303074	Both
cg24304714	Both
cg24474182	Both
cg26832142	Both
cg05564266	Both
cg07017706	Both
cg26309134	Both
cg04988423	Both
cg20357806	Both
cg06275635	Both
cg13434203	Both
cg00654814	Both
cg22571530	Both
cg03538436	Both
cg13406768	Both
cg08020808	Both

cg06940792	Both
cg02387679	Both
cg02932689	Both
cg25426302	Both
cg22252999	Both
cg01000094	Both
cg09208010	Both
cg25612145	Both
cg01720520	Both
cg21094669	Both
cg05948654	Both
cg23621115	Both
cg14173523	Both
cg02150910	Both
cg08210616	Both
cg02613803	Both
cg00292971	Both
cg10057295	Both
cg03037331	Both
cg11492040	Both
cg03320844	Both
cg14679202	Both
cg21376883	Both
cg02676865	Both
cg11051158	Both
cg16745604	Both
cg22289837	Both
cg04803153	Both
cg17833578	Both
cg09137696	Both
cg12585943	Both
cg23671708	Both
cg14832904	Both
cg13847070	Both
cg22176895	Both
cg20993403	Both
cg01015871	Both
cg16302441	Both
cg05779068	Both
cg22767466	Both
cg06910100	Both
cg16264526	Both
cg22477971	Both
cg21736592	Both
cg24924779	Both
cg21608192	Both
cg04459030	Both
cg16989646	Both
cg24711914	Both
cg04749104	Both
cg23777956	Both
cg11380624	Both
cg23661676	Both

cg14338062	Both
cg05404787	Both
cg02564061	Both
cg24938727	Both
cg24546463	Both
cg08109646	Both
cg13676215	Both
cg04446579	Both
cg16205058	Both
cg09214254	Both
cg04790874	Both
cg17461214	Both
cg04114315	Both
cg00012199	Both
cg20924286	Both
cg22228134	Both
cg01155039	Both
cg01420388	Both
cg24178740	Both
cg03716937	Both
cg16101800	Both
cg05467458	Both
cg15883716	Both
cg02162069	Both
cg02833725	Both
cg25608949	Both
cg18884137	Both
cg06650260	Both
cg07456645	Both
cg02490034	Both
cg18592174	Both
cg12810837	Both
cg05650171	Both
cg04272086	Both
cg09079275	Both
cg10723020	Both
cg12128017	Both
cg17823175	Both
cg14256699	Both
cg05646865	Both
cg14449575	Both
cg26427308	Both
cg15383120	Both
cg13885201	Both
cg23126915	Both
cg11063110	Both
cg22876908	Both
cg26986236	Both
cg19850348	Both
cg23357981	Both
cg15052901	Both
cg07572341	Both
cg26792080	Both

cg00412772	Both
cg19461644	Both
cg08694544	Both
cg03218909	Both
cg27637521	Both
cg22585988	Both
cg20675440	Both
cg01598642	Both
cg27394566	Both
cg27588902	Both
cg11378840	Both
cg23219570	Both
cg01567482	Both
cg03143849	Both
cg18565355	Both
cg22820108	Both
cg23043245	Both
cg03794550	Both
cg26556719	Both
cg14415300	Both
cg17631429	Both
cg09374949	Both
cg01987509	Both
cg11639651	Both
cg02473123	Both
cg15658426	Both
cg27269917	Both
cg12278770	Both
cg17803089	Both
cg20624391	Both
cg21093060	Both
cg04144768	Both
cg06201642	Both
cg01656750	Both
cg20959866	Both
cg08578734	Both
cg25766046	Both
cg12910797	Both
cg17571291	Both
cg15740508	Both
cg08996521	Both
cg02780849	Both
cg02512226	Both
cg18250832	Both
cg00509670	Both
cg12391048	Both
cg23743472	Both
cg13530946	Both
cg18221897	Both
cg05633152	Both
cg09411212	Both
cg26218269	Both
cg08785215	Both

cg15417244	Both
cg12351042	Both
cg24051818	Both
cg08508325	Both
cg00445824	Both
cg13202751	Both
cg08451957	Both
cg26215727	Both
cg23129478	Both
cg21577049	Both
cg16168311	Both
cg27104271	Both
cg01999333	Both
cg04569804	Both
cg05203877	Both
cg10989517	Both
cg01720228	Both
cg11933267	Both
cg17194182	Both
cg19856444	Both
cg14260458	Both
cg15802323	Both
cg12348970	Both
cg24505375	Both
cg05070690	Both
cg15341340	Both
cg11080718	Both
cg03368758	Both
cg06563300	Both
cg18408326	Both
cg06868758	Both
cg17021265	Both
cg03401114	Both
cg26149678	Both
cg20544605	Both
cg15599064	Both
cg18794577	Both
cg20066677	Both
cg13230197	Both
cg08459368	Both
cg07621046	Both
cg01305421	Both
cg19776201	Both
cg09306675	Both
cg13289321	Both
cg05592398	Both
cg21073927	Both
cg06937608	Both
cg26309498	Both
cg04048259	Both
cg03544379	Both
cg02304930	Both
cg02909790	Both

cg15836660	Both
cg18016365	Both
cg16177163	Both
cg24248317	Both
cg11378484	Both
cg24030449	Both
cg04603031	Both
cg06653796	Both
cg27560922	Both
cg03086857	Both
cg03843978	Both
cg00031162	Both
cg19524009	Both
cg14506552	Both
cg15495837	Both
cg03317245	Both
cg24926780	Both
cg16381688	Both
cg07918509	Both
cg10670077	Both
cg10862848	Both
cg25025866	Both
cg19764555	Both
cg14528319	Both
cg02595219	Both
cg19646028	Both
cg24812103	Both
cg04265576	Both
cg13500819	Both
cg15059932	Both
cg15756091	Both
cg18735146	Both
cg08539991	Both
cg01335367	Both
cg09009380	Both
cg16993794	Both
cg24735937	Both
cg25028542	Both
cg04337944	Both
cg17250929	Both
cg09646392	Both
cg15749748	Both
cg09971646	Both
cg04600618	Both
cg18534730	Both
cg05822532	Both
cg25406518	Both
cg06811800	Both
cg14043602	Both
cg02630694	Both
cg17987660	Both
cg25771195	Both
cg15425280	Both

cg20092036	Both
cg04394102	Both
cg20718816	Both
cg06645778	Both
cg24512400	Both
cg27425675	Both
cg14346035	Both
cg03100752	Both
cg24251035	Both
cg02763671	Both
cg16413535	Both
cg11812202	Both
cg03292149	Both
cg04520391	Both
cg03584220	Both
cg13836770	Both
cg05961827	Both
cg08887581	Both
cg06516124	Both
cg19461621	Both
cg18454685	Both
cg26866014	Both
cg11939071	Both
cg26112639	Both
cg24968336	Both
cg10300154	Both
cg06362985	Both
cg06882926	Both
cg22630748	Both
cg14757296	Both
cg17404915	Both
cg21880903	Both
cg14062083	Both
cg26616347	Both
cg17067005	Both
cg03336167	Both
cg22421766	Both
cg09196942	Both
cg00576250	Both
cg20845050	Both
cg22055427	Both
cg21434954	Both
cg21512370	Both
cg15784332	Both
cg27493997	Both
cg20158248	Both
cg09059635	Both
cg20651453	Both
cg14120879	Both
cg04653021	Both
cg15952487	Both
cg04172348	Both
cg20988616	Both

cg00160914	Both
cg11656547	Both
cg08687901	Both
cg06589885	Both
cg22764925	Both
cg02417408	Both
cg26728886	Both
cg19560971	Both
cg10634551	Both
cg14404298	Both
cg07328579	Both
cg13823136	Both
cg13447818	Both
cg11828089	Both
cg11800672	Both
cg26750319	Both
cg03958979	Both
cg16470760	Both
cg09554443	Both
cg25384157	Both
cg12091331	Both
cg19635695	Both
cg18201077	Both
cg09261262	Both
cg01443630	Both
cg27592112	Both
cg06638433	Both
cg25355803	Both
cg08349806	Both
cg07465864	Both
cg25964007	Both
cg17288121	Both
cg11943820	Both
cg26417554	Both
cg14925024	Both
cg09045681	Both
cg06319579	Both
cg11976790	Both
cg18365380	Both
cg25584626	Both
cg26609631	Both
cg19356324	Both
cg21081971	Both
cg12584718	Both
cg21639401	Both
cg14262937	Both
cg15387123	Both
cg02329886	Both
cg02765820	Both
cg22789545	Both
cg17619823	Both
cg26244225	Both
cg21589115	Both

cg04705866	Both
cg04764624	Both
cg09582042	Both
cg01471384	Both
cg12128839	Both
cg26751195	Both
cg09276363	Both
cg17974185	Both
cg10676060	Both
cg16708281	Both
cg21250978	Both
cg15905124	Both
cg16855929	Both
cg01493517	Both
cg23641145	Both
cg12331389	Both
cg24834740	Both
cg17173856	Both
cg22218909	Both
cg00071250	Both
cg14296374	Both
cg19974223	Both
cg23887102	Both
cg04341806	Both
cg12952132	Both
cg11038843	Both
cg10137010	Both
cg02836529	Both
cg08450982	Both
cg17291001	Both
cg08816023	Both
cg07550267	Both
cg25167643	Both
cg20277670	Both
cg16953612	Both
cg17790333	Both
cg23815306	Both
cg23111544	Both
cg02202484	Both
cg05159188	Both
cg13152535	Both
cg14552982	Both
cg08080029	Both
cg22821324	Both
cg08136806	Both
cg06204948	Both
cg24603941	Both
cg13948987	Both
cg19839588	Both
cg17575811	Both
cg10889792	Both
cg11378686	Both
cg19968631	Both

cg10989326	Both
cg07915343	Both
cg09595479	Both
cg04329382	Both
cg26656135	Both
cg05279864	Both
cg15616400	Both
cg00658626	Both
cg13397379	Both
cg07421682	Both
cg24113449	Both
cg25905812	Both
cg14204735	Both
cg00705255	Both
cg24777950	Both
cg13594711	Both
cg12109455	Both
cg04802221	Both
cg17964955	Both
cg17391877	Both
cg12218747	Both
cg09705062	Both
cg10186456	Both
cg16935609	Both
cg22940152	Both
cg21838334	Both
cg01236137	Both
cg13765961	Both
cg12343638	Both
cg04060275	Both
cg03883348	Both
cg22750254	Both
cg07239938	Both
cg23621689	Both
cg13496736	Both
cg25535027	Both
cg15737319	Both
cg12374577	Both
cg23917399	Both
cg10167296	Both
cg27009703	Both
cg16984944	Both
cg14089692	Both
cg12262564	Both
cg16893574	Both
cg26623286	Both
cg21250296	Both
cg16303453	Both
cg10313673	Both
cg10671066	Both
cg24626923	Both
cg05245515	Both
cg16749578	Both

cg05997860	Both
cg07658590	Both
cg04807655	Both
cg21922731	Both
cg13929970	Both
cg25425074	Both
cg24910675	Both
cg26401870	Both
cg11688990	Both
cg17092637	Both
cg05656180	Both
cg13350783	Both
cg08958913	Both
cg15811427	Both
cg16478145	Both
cg03190825	Both
cg18841952	Both
cg08872493	Both
cg18972811	Both
cg00585790	Both
cg20789620	Both
cg12555907	Both
cg18484189	Both
cg17692403	Both
cg11752275	Both
cg04366367	Both
cg21211748	Both
cg10356463	Both
cg17942553	Both
cg02144298	Both
cg02989257	Both
cg05376954	Both
cg15976539	Both
cg15154229	Both
cg25266232	Both
cg25310567	Both
cg07443748	Both
cg21550442	Both
cg12052765	Both
cg13707560	Both
cg27246571	Both
cg15428653	Both
cg20720686	Both
cg02184413	Both
cg14784653	Both
cg06143901	Both
cg15775914	Both
cg23750142	Both
cg27420236	Both
cg20082641	Both
cg02953306	Both
cg25186143	Both
cg18718102	Both

cg21820677	Both
cg20276750	Both
cg21169285	Both
cg27436184	Both
cg18074297	Both
cg25203856	Both
cg06151165	Both
cg15660077	Both
cg23159337	Both
cg16954341	Both
cg02240622	Both
cg10430690	Both
cg07639720	Both
cg19286604	Both
cg02100848	Both
cg15337897	Both
cg04958389	Both
cg12685753	Both
cg27377450	Both
cg18347238	Both
cg01129459	Both
cg08221207	Both
cg13179915	Both
cg21553596	Both
cg17580045	Both
cg19798224	Both
cg04541293	Both
cg19091641	Both
cg25191725	Both
cg19363546	Both
cg24759821	Both
cg07894717	Both
cg19250891	Both
cg23149053	Both
cg03599338	Both
cg27336379	Both
cg12242338	Both
cg26760212	Both
cg01599709	Both
cg06577005	Both
cg20035459	Both
cg00480115	Both
cg10484958	Both
cg14948436	Both
cg21665000	Both
cg07044282	Both
cg21602520	Both
cg22994720	Both
cg00816620	Both
cg18956481	Both
cg04637372	Both
cg01541443	Both
cg25808906	Both

cg04747322	Both
cg11612345	Both
cg24505122	Both
cg20145360	Both
cg25558099	Both
cg10910775	Both
cg14913610	Both
cg05131835	Both
cg23566503	Both
cg10149836	Both
cg23428445	Both
cg26119740	Both
cg03221619	Both
cg08983760	Both
cg00240432	Both
cg22373097	Both
cg27226949	Both
cg15901783	Both
cg05016953	Both
cg18109798	Both
cg03014957	Both
cg13585240	Both
cg21643361	Both
cg24435562	Both
cg00234616	Both
cg06849477	Both
cg09238677	Both
cg25524350	Both
cg04200192	Both
cg19188612	Both
cg08300860	Both
cg05910970	Both
cg17532978	Both
cg02815516	Both
cg17303299	Both
cg22882178	Both
cg07897701	Both
cg23398241	Both
cg03679734	Both
cg18450227	Both
cg01880569	Both
cg22533573	Both
cg08947964	Both
cg05228408	Both
cg12240237	Both
cg06110728	Both
cg05859842	Both
cg24024214	Both
cg10604646	Both
cg14578030	Both
cg26727372	Both
cg23984130	Both
cg07605143	Both

cg26822175	Both
cg24520070	Both
cg17100200	Both
cg04230060	Both
cg23762517	Both
cg19751300	Both
cg16582174	Both
cg01197831	Both
cg24010952	Both
cg25957124	Both
cg19530885	Both
cg15870225	Both
cg17091851	Both
cg21495715	Both
cg12008118	Both
cg23538064	Both
cg18463686	Both
cg17217654	Both
cg08186362	Both
cg21607649	Both
cg08997253	Both
cg19282250	Both
cg03789934	Both
cg24926042	Both
cg11462533	Both
cg09405083	Both
cg17468997	Both
cg11469778	Both
cg19426277	Both
cg13959523	Both
cg04743872	Both
cg08477744	Both
cg11847808	Both
cg07884019	Both
cg14228238	Both
cg19632760	Both
cg06656924	Both
cg22442730	Both
cg06352750	Both
cg14679587	Both
cg19063397	Both
cg25293251	Both
cg01962826	Both
cg02972551	Both
cg23163573	Both
cg25215340	Both
cg26628847	Both
cg00846036	Both
cg20342105	Both
cg17190608	Both
cg22524514	Both
cg02064106	Both
cg19219366	Both

cg12144803	Both
cg12928668	Both
cg18081104	Both
cg17255302	Both
cg17505463	Both
cg21274570	Both
cg10798171	Both
cg00135056	Both
cg12588299	Both
cg10634424	Both
cg14277764	Both
cg08003742	Both
cg18671950	Both
cg01726767	Both
cg05798712	Both
cg01459453	Both
cg09358725	Both
cg07950803	Both
cg10414946	Both
cg24950749	Both
cg01172972	Both
cg00344372	Both
cg20349377	Both
cg12732155	Both
cg06848073	Both
cg05382123	Both
cg26987645	Both
cg12622986	Both
cg03574571	Both
cg27641018	Both
cg18750960	Both
cg27579745	Both
cg26647600	Both
cg15869022	Both
cg14086647	Both
cg08445039	Both
cg12603560	Both
cg20781967	Both
cg02161900	Both
cg02237186	Both
cg21631754	Both
cg25361844	Both
cg18056600	Both
cg26845300	Both
cg14738823	Both
cg11877382	Both
cg08723608	Both
cg17389519	Both
cg19703610	Both
cg03224418	Both
cg04325632	Both
cg22609784	Both
cg22487322	Both

cg19132372	Both
cg02738086	Both
cg05421688	Both
cg16158220	Both
cg12354377	Both
cg11710969	Both
cg16761581	Both
cg06946880	Both
cg27609819	Both
cg05368341	Both
cg08812936	Both
cg09872233	Both
cg14159672	Both
cg19172575	Both
cg20583073	Both
cg13335112	Both
cg21824213	Both
cg02126753	Both
cg15550350	Both
cg12153542	Both
cg25711779	Both
cg15616083	Both
cg27547703	Both
cg24892510	Both
cg06022562	Both
cg01637734	Both
cg13412615	Both
cg15662251	Both
cg26365854	Both
cg05225996	Both
cg09986574	Both
cg05724065	Both
cg00273068	Both
cg19283196	Both
cg02600394	Both
cg16387606	Both
cg11004890	Both
cg03415518	Both
cg25623640	Both
cg12485992	Both
cg02721374	Both
cg00195561	Both
cg02806777	Both
cg22794078	Both
cg20158826	Both
cg12514620	Both
cg22843446	Both
cg25713185	Both
cg13210534	Both
cg11237817	Both
cg08321346	Both
cg04991214	Both
cg00107488	Both

cg25829729	Both
cg06346081	Both
cg11115427	Both
cg16685388	Both
cg20557202	Both
cg09868882	Both
cg14070162	Both
cg14029663	Both
cg05894797	Both
cg14070647	Both
cg19326876	Both
cg19836283	Both
cg03109701	Both
cg18871276	Both
cg08995424	Both
cg10694152	Both
cg23166362	Both
cg06151964	Both
cg13492340	Both
cg20113732	Both
cg07613153	Both
cg19136075	Both
cg01643624	Both
cg14349467	Both
cg08623947	Both
cg26227465	Both
cg13552869	Both
cg12600197	Both
cg21478437	Both
cg17040807	Both
cg03156547	Both
cg11520395	Both
cg11527279	Both
cg16519321	Both
cg18345369	Both
cg20468883	Both
cg24825722	Both
cg08383315	Both
cg10923408	Both
cg10159529	Both
cg05307923	Both
cg14137939	Both
cg18468844	Both
cg15564267	Both
cg01566170	Both
cg17179881	Both
cg26301777	Both
cg13626881	Both
cg15085805	Both
cg18042806	Both
cg20695587	Both
cg13002506	Both
cg11997899	Both

cg27555365	Both
cg18876487	Both
cg09580336	Both
cg18979491	Both
cg18530748	Both
cg09754413	Both
cg00653387	Both
cg00848461	Both
cg27488807	Both
cg05941634	Both
cg17181653	Both
cg10862587	Both
cg13156121	Both
cg18885346	Both
cg02436686	Both
cg14724613	Both
cg03586879	Both
cg18342279	Both
cg20383064	Both
cg03934354	Both
cg00666446	Both
cg25040783	Both
cg01678091	Both
cg07714374	Both
cg13346411	Both
cg18843688	Both
cg01839464	Both
cg06940107	Both
cg06256735	Both
cg17747005	Both
cg05275752	Both
cg19226099	Both
cg00848728	Both
cg16098726	Both
cg27600794	Both
cg20535085	Both
cg00550617	Both
cg15765694	Both
cg22445920	Both
cg01291404	Both
cg25152942	Both
cg20587168	Both
cg22778947	Both
cg24358465	Both
cg11917694	Both
cg23698969	Both
cg06907544	Both
cg23263923	Both
cg26873164	Both
cg00534856	Both
cg11320084	Both
cg06497752	Both
cg09304624	Both

cg17108819	Both
cg18277754	Both
cg20770175	Both
cg17749443	Both
cg17918700	Both
cg17738133	Both
cg15522957	Both
cg09429185	Both
cg09997760	Both
cg26651233	Both
cg06640279	Both
cg05350879	Both
cg18353563	Both
cg09637363	Both
cg01474260	Both
cg12417466	Both
cg25866075	Both
cg00852964	Both
cg02994974	Both
cg06791867	Both
cg18951427	Both
cg11902458	Both
cg23865698	Both
cg12556325	Both
cg03116238	Both
cg07475743	Both
cg13666729	Both
cg15319457	Both
cg20664247	Both
cg23097006	Both
cg16155702	Both
cg23165541	Both
cg20914508	Both
cg07883333	Both
cg14156381	Both
cg26399035	Both
cg07384961	Both
cg26976437	Both
cg04700814	Both
cg20492933	Both
cg27416067	Both
cg12064929	Both
cg26531804	Both
cg14356550	Both
cg26141626	Both
cg19384697	Both
cg01401376	Both
cg16026550	Both
cg17894008	Both
cg01137065	Both
cg07675169	Both
cg19250907	Both
cg25082710	Both

cg14567401	Both
cg21697134	Both
cg09276451	Both
cg03958426	Both
cg24607398	Both
cg14541950	Both
cg15403517	Both
cg23496260	Both
cg05484458	Both
cg09099177	Both
cg27504299	Both
cg01274660	Both
cg13619990	Both
cg22407504	Both
cg23350580	Both
cg23349790	Both
cg16017492	Both
cg01795161	Both
cg21652958	Both
cg01869233	Both
cg23776012	Both
cg20182358	Both
cg00509616	Both
cg26493932	Both
cg00405677	Both
cg01464985	Both
cg20171451	Both
cg23090046	Both
cg08504583	Both
cg20986134	Both
cg18692273	Both
cg10214058	Both
cg14759043	Both
cg22215728	Both
cg13343486	Both
cg07337598	Both
cg10071275	Both
cg20028470	Both
cg08935301	Both
cg23773532	Both
cg01982455	Both
cg27067618	Both
cg00132141	Both
cg27495845	Both
cg02064402	Both
cg11378052	Both
cg07973967	Both
cg23260026	Both
cg03044435	Both
cg21480743	Both
cg06521280	Both
cg14736210	Both
cg17641252	Both

cg24901042	Both
cg17897879	Both
cg25511429	Both
cg21271753	Both
cg09014354	Both
cg19205041	Both
cg19211827	Both
cg22800631	Both
cg27655855	Both
cg20916523	Both
cg00426498	Both
cg20357628	Both
cg20312687	Both
cg19553721	Both
cg15669228	Both
cg04431054	Both
cg05898524	Both
cg23514672	Both
cg26759925	Both
cg17839611	Both
cg22825487	Both
cg17127823	Both
cg14916288	Both
cg12439773	Both
cg01281797	Both
cg26159905	Both
cg16786458	Both
cg16899036	Both
cg12819826	Both
cg25461934	Both
cg07830847	Both
cg06330621	Both
cg05078019	Both
cg21065959	Both
cg09619146	Both
cg24392274	Both
cg07744166	Both
cg03588357	Both
cg26808784	Both
cg16248277	Both
cg21287330	Both
cg12629244	Both
cg02784874	Both
cg21530453	Both
cg16257091	Both
cg10692870	Both
cg16087263	Both
cg01557989	Both
cg06254453	Both
cg16352283	Both
cg00987379	Both
cg03721967	Both
cg12914014	Both

cg10106284	Both
cg24826867	Both
cg09227563	Both
cg03312532	Both
cg00546897	Both
cg01404615	Both
cg00601486	Both
cg27363486	Both
cg06537230	Both
cg26361928	Both
cg08191915	Both
cg09027725	Both
cg25229964	Both
cg19636871	Both
cg26757673	Both
cg02225847	Both
cg23363832	Both
cg00000292	Both
cg11845202	Both
cg03945800	Both
cg12441928	Both
cg08375941	Both
cg23092086	Both
cg14045072	Both
cg25047001	Both
cg00090147	Both
cg07022477	Both
cg18138500	Both
cg07059360	Both
cg22436229	Both
cg02164442	Both
cg13337865	Both
cg17272843	Both
cg07824742	Both
cg08411435	Both
cg20587394	Both
cg02756698	Both
cg15751406	Both
cg24642523	Both
cg14036856	Both
cg20541456	Both
cg01469547	Both
cg05414338	Both
cg19470701	Both
cg27281093	Both
cg09404633	Both
cg14423778	Both
cg02096656	Both
cg19789466	Both
cg02605634	Both
cg14078518	Both
cg23272214	Both
cg02870945	Both

cg10331038	Both
cg05575043	Both
cg17939444	Both
cg12622273	Both
cg03382304	Both
cg18623836	Both
cg00512279	Both
cg22635155	Both
cg00453258	Both
cg21898046	Both
cg12001148	Both
cg01785568	Both
cg05922591	Both
cg19546781	Both
cg05384917	Both
cg01161611	Both
cg05010058	Both
cg05342835	Both
cg08831348	Both
cg21815667	Both
cg10227191	Both
cg20449692	Both
cg05429895	Both
cg11710560	Both
cg18676053	Both
cg19208681	Both
cg11494699	Both
cg00687686	Both
cg05564251	Both
cg01985396	Both
cg15034135	Both
cg05098732	Both
cg07241568	Both
cg20775254	Both
cg02273392	Both
cg01305625	Both
cg23521281	Both
cg02992632	Both
cg15379633	Both
cg00819362	Both
cg22463915	Both
cg16660041	Both
cg18107072	Both
cg06339657	Both
cg09527362	Both
cg18138484	Both
cg13181284	Both
cg02628202	Both
cg25533774	Both
cg07629017	Both
cg03521347	Both
cg05830416	Both
cg20839149	Both

cg21474838	Both
cg19774122	Both
cg18963171	Both
cg25948180	Both
cg11346450	Both
cg21342728	Both
cg25162921	Both
cg21852292	Both
cg21049762	Both
cg05590257	Both
cg08516400	Both
cg06264060	Both
cg13270853	Both
cg07054641	Both
cg07197059	Both
cg15043057	Both
cg05252264	Both
cg22643217	Both
cg15419274	Both
cg19547629	Both
cg26365668	Both
cg11935638	Both
cg04684516	Both
cg18676162	Both
cg13185177	Both
cg14861570	Both
cg15083233	Both
cg23748737	Both
cg04329069	Both
cg25059899	Both
cg14120436	Both
cg07685786	Both
cg07671208	Both
cg15044041	Both
cg02309273	Both
cg15439196	Both
cg24357161	Both
cg15210999	Both
cg22799850	Both
cg20727114	Both
cg18752880	Both
cg20352371	Both
cg04676561	Both
cg09226684	Both
cg25417405	Both
cg14764661	Both
cg19955521	Both
cg15648315	Both
cg11757293	Both
cg23637791	Both
cg14500718	Both
cg18022344	Both
cg24992780	Both

cg25946389	Both
cg19042062	Both
cg00214794	Both
cg23240895	Both
cg01603095	Both
cg21970438	Both
cg22244418	Both
cg07686479	Both
cg07112154	Both
cg08205865	Both
cg07816439	Both
cg17256157	Both
cg06353345	Both
cg23889010	Both
cg27132814	Both
cg05056120	Both
cg17084151	Both
cg10173075	Both
cg14900471	Both
cg23984434	Both
cg20207753	Both
cg05637351	Both
cg12707353	Both
cg14400118	Both
cg20425130	Both
cg10872212	Both
cg16051027	Both
cg14912575	Both
cg08223235	Both
cg15248035	Both
cg04335339	Both
cg26697117	Both
cg07036530	Both
cg21428681	Both
cg09841009	Both
cg14472601	Both
cg22113807	Both
cg15357639	Both
cg03547797	Both
cg21048669	Both
cg01324261	Both
cg19832721	Both
cg22153873	Both
cg22775000	Both
cg15459773	Both
cg13628514	Both
cg19481686	Both
cg25195673	Both
cg02982734	Both
cg12774845	Both
cg27069753	Both
cg10187559	Both
cg26416466	Both

cg10896774	Both
cg03872376	Both
cg18084554	Both
cg08999895	Both
cg04582295	Both
cg20855344	Both
cg16422907	Both
cg08784110	Both
cg23957915	Both
cg25226247	Both
cg19889584	Both
cg10078415	Both
cg12838902	Both
cg27044702	Both
cg14238120	Both
cg27566805	Both
cg25462291	Both
cg16787600	Both
cg24816298	Both
cg21335556	Both
cg14088811	Both
cg16098981	Both
cg03476195	Both
cg14386548	Both
cg10990993	Both
cg03270167	Both
cg00940891	Both
cg20311730	Both
cg06069731	Both
cg06407137	Both
cg23696949	Both
cg10810921	Both
cg26530341	Both
cg13342558	Both
cg07888234	Both
cg02712878	Both
cg10938286	Both
cg11847949	Both
cg07042144	Both
cg22919728	Both
cg02248486	Both
cg25141490	Both
cg10822172	Both
cg02432101	Both
cg02833180	Both
cg26353877	Both
cg24776407	Both
cg10635061	Both
cg13181019	Both
cg16571399	Both
cg15298323	Both
cg09120035	Both
cg08896053	Both

cg16791508	Both
cg03112433	Both
cg22506059	Both
cg27337148	Both
cg06197492	Both
cg10332700	Both
cg25833031	Both
cg20162076	Both
cg12864581	Both
cg08012287	Both
cg00930873	Both
cg16517394	Both
cg21468416	Both
cg17277939	Both
cg11812218	Both
cg14847688	Both
cg15461516	Both
cg18628483	Both
cg02882813	Both
cg22679120	Both
cg08446111	Both
cg07899016	Both
cg08965143	Both
cg24107665	Both
cg08274552	Both
cg21950518	Both
cg13164537	Both
cg25596297	Both
cg06039392	Both
cg02990033	Both
cg16890267	Both
cg14808739	Both
cg14662379	Both
cg09892203	Both
cg26924825	Both
cg16213655	Both
cg26682500	Both
cg12145907	Both
cg08013810	Both
cg25431974	Both
cg09660171	Both
cg06948294	Both
cg08532057	Both
cg11102782	Both
cg06384463	Both
cg20114394	Both
cg02196655	Both
cg08858521	Both
cg20491707	Both
cg20150565	Both
cg12127282	Both
cg04893119	Both
cg00468146	Both

cg07412254	Both
cg09038914	Both
cg23909633	Both
cg10681065	Both
cg12717203	Both
cg13435381	Both
cg01521624	Both
cg03617456	Both
cg23322523	Both
cg01680823	Both
cg21542793	Both
cg22284302	Both
cg05607401	Both
cg25999867	Both
cg00465284	Both
cg14917512	Both
cg15903395	Both
cg02008416	Both
cg17550582	Both
cg10141715	Both
cg05846716	Both
cg10177528	Both
cg15083227	Both
cg03213216	Both
cg15361750	Both
cg21256656	Both
cg25014318	Both
cg23693510	Both
cg23503501	Both
cg04985144	Both
cg13843613	Both
cg03257423	Both
cg09768051	Both
cg20322977	Both
cg04389838	Both
cg20330472	Both
cg17169998	Both
cg25141995	Both
cg11389172	Both
cg21151355	Both
cg25552492	Both
cg21472642	Both
cg16986846	Both
cg13904968	Both
cg12268344	Both
cg21237418	Both
cg02332537	Both
cg16826718	Both
cg19803984	Both
cg10503234	Both
cg11617144	Both
cg10371914	Both
cg18133957	Both

cg12850636	Both
cg27076139	Both
cg25483003	Both
cg22995106	Both
cg10379687	Both
cg07713493	Both
cg00269932	Both
cg26514942	Both
cg14014225	Both
cg24905739	Both
cg23582919	Both
cg17959722	Both
cg08700651	Both
cg13960126	Both
cg18408487	Both
cg15261665	Both
cg22182945	Both
cg22858308	Both
cg03556497	Both
cg19654437	Both
cg15661409	Both
cg20286200	Both
cg21450627	Both
cg24166628	Both
cg15679095	Both
cg20377673	Both
cg07597976	Both
cg14134005	Both
cg06661994	Both
cg12610070	Both
cg16391792	Both
cg01804429	Both
cg17946995	Both
cg02017109	Both
cg07970007	Both
cg01281904	Both
cg19246110	Both
cg14498423	Both
cg23125689	Both
cg24311282	Both
cg11835197	Both
cg26557658	Both
cg23271318	Both
cg20856834	Both
cg17761453	Both
cg01143454	Both
cg25569462	Both
cg07260017	Both
cg01109219	Both
cg06797533	Both
cg23243617	Both
cg00386408	Both
cg19553463	Both

cg21832243	Both
cg26199493	Both
cg03956628	Both
cg12087643	Both
cg10398682	Both
cg13548361	Both
cg05386606	Both
cg24287460	Both
cg00896220	Both
cg18888403	Both
cg16106497	Both
cg04314463	Both
cg00131557	Both
cg14201617	Both
cg06007645	Both
cg05492113	Both
cg17774418	Both
cg26298099	Both
cg15518883	Both
cg10934032	Both
cg21096399	Both
cg03522910	Both
cg14577211	Both
cg00875272	Both
cg26114571	Both
cg18913951	Both
cg27060381	Both
cg22777952	Both
cg19709058	Both
cg21432513	Both
cg17191178	Both
cg12294121	Both
cg10784341	Both
cg19794490	Both
cg09462576	Both
cg17687282	Both
cg11173246	Both
cg15264303	Both
cg15642035	Both
cg19006008	Both
cg01970325	Both
cg14473924	Both
cg02330106	Both
cg12412075	Both
cg15720535	Both
cg25687894	Both
cg16290737	Both
cg15544721	Both
cg06425868	Both
cg05596294	Both
cg10523019	Both
cg22517351	Both
cg17836145	Both

cg15772361	Both
cg01580888	Both
cg20047055	Both
cg02992647	Both
cg16682903	Both
cg21663122	Both
cg06896207	Both
cg26815229	Both
cg02500392	Both
cg10280342	Both
cg07965823	Both
cg06363129	Both
cg10335112	Both
cg12382902	Both
cg18034859	Both
cg16606638	Both
cg18881269	Both
cg06173079	Both
cg17611475	Both
cg19136717	Both
cg04109382	Both
cg06784466	Both
cg04925864	Both
cg04052038	Both
cg21942082	Both
cg06815666	Both
cg11619390	Both
cg17471928	Both
cg13853198	Both
cg25928444	Both
cg16581199	Both
cg20000468	Both
cg14679230	Both
cg23871933	Both
cg01307730	Both
cg24127874	Both
cg18730023	Both
cg23472215	Both
cg22082462	Both
cg01683883	Both
cg17922226	Both
cg04184278	Both
cg08818784	Both
cg01564343	Both
cg09799714	Both
cg14314889	Both
cg05191071	Both
cg12114524	Both
cg19717326	Both
cg06810647	Both
cg16192029	Both
cg12729048	Both
cg18601426	Both

cg22972055	Both
cg25033144	Both
cg21039679	Both
cg24719984	Both
cg08054038	Both
cg22930187	Both
cg25228746	Both
cg19795898	Both
cg14034870	Both
cg14261309	Both
cg16097772	Both
cg18740800	Both
cg25117362	Both
cg23074401	Both
cg27360003	Both
cg08675585	Both
cg27138584	Both
cg15092802	Both
cg09702010	Both
cg11673092	Both
cg26907768	Both
cg24222324	Both
cg07484827	Both
cg15792688	Both
cg01355739	Both
cg16773028	Both
cg25221625	Both
cg22776578	Both
cg01517728	Both
cg17385448	Both
cg20516209	Both
cg18630040	Both
cg27196745	Both
cg14477619	Both
cg14984920	Both
cg21407055	Both
cg09837803	Both
cg23127998	Both
cg26384034	Both
cg08418332	Both
cg00098239	Both
cg09207718	Both
cg02010852	Both
cg20947775	Both
cg22498251	Both
cg26845278	Both
cg05606799	Both
cg10688991	Both
cg20907471	Both
cg15095327	Both
cg00117172	Both
cg24310246	Both
cg20308679	Both

cg16722536	Both
cg11657808	Both
cg07955887	Both
cg11042320	Both
cg04322134	Both
cg23282674	Both
cg25040282	Both
cg20267005	Both
cg22386774	Both
cg02604290	Both
cg13640200	Both
cg12259537	Both
cg03764585	Both
cg11159299	Both
cg04420907	Both
cg19125606	Both
cg08908355	Both
cg26264314	Both
cg26216632	Both
cg11525285	Both
cg13974531	Both
cg24734575	Both
cg22646710	Both
cg21948783	Both
cg25965576	Both
cg04457051	Both
cg21209091	Both
cg00431114	Both
cg21179457	Both
cg23004625	Both
cg17078393	Both
cg11884699	Both
cg11657615	Both
cg22037648	Both
cg20483374	Both
cg01237132	Both
cg22064451	Both
cg05897048	Both
cg11474811	Both
cg12108912	Both
cg20073553	Both
cg24476569	Both
cg26620356	Both
cg13619915	Both
cg02586730	Both
cg21745164	Both
cg00168785	Both
cg19764418	Both
cg24459563	Both
cg03386373	Both
cg05657090	Both
cg05973262	Both
cg03400060	Both

cg18123948	Both
cg06118312	Both
cg18204685	Both
cg13975369	Both
cg02937479	Both
cg11500727	Both
cg02599464	Both
cg09503974	Both
cg03873930	Both
cg09852871	Both
cg23402444	Both
cg19889780	Both
cg05896682	Both
cg19162106	Both
cg04558553	Both
cg13384498	Both
cg09076012	Both
cg04897683	Both
cg04527918	Both
cg05052633	Both
cg17054360	Both
cg15087147	Both
cg10135708	Both
cg01704534	Both
cg04254916	Both
cg21988041	Both
cg12619509	Both
cg20687462	Both
cg05851042	Both
cg24346637	Both
cg03311036	Both
cg20676475	Both
cg03941108	Both
cg09390932	Both
cg18153060	Both
cg19589427	Both
cg21023001	Both
cg22416721	Both
cg17281600	Both
cg13877895	Both
cg11585425	Both
cg21321735	Both
cg11811840	Both
cg27530424	Both
cg25934198	Both
cg20790540	Both
cg14948822	Both
cg14686321	Both
cg07642638	Both
cg16899306	Both
cg09088576	Both
cg25341726	Both
cg11767711	Both

cg26680127	Both
cg02765328	Both
cg08491964	Both
cg25999015	Both
cg06154597	Both
cg27040030	Both
cg10143146	Both
cg16907024	Both
cg14348532	Both
cg13547237	Both
cg24694549	Both
cg18248112	Both
cg15376097	Both
cg14566624	Both
cg05449414	Both
cg01321151	Both
cg03399459	Both
cg21459921	Both
cg18414381	Both
cg02361557	Both
cg10620457	Both
cg16449464	Both
cg14709481	Both
cg09350274	Both
cg23499956	Both
cg07903860	Both
cg04960652	Both
cg19782075	Both
cg00714377	Both
cg23065097	Both
cg21643045	Both
cg17031727	Both
cg22036988	Both
cg22284975	Both
cg17031773	Both
cg17286640	Both
cg25889160	Both
cg24686358	Both
cg24889744	Both
cg13877915	Both
cg18557145	Both
cg00746981	Both
cg13214422	Both
cg02401149	Both
cg05072951	Both
cg01519742	Both
cg00660989	Both
cg17327492	Both
cg12943082	Both
cg24574111	Both
cg15557833	Both
cg01414934	Both
cg01407244	Both

cg19848683	Both
cg27239157	Both
cg16632715	Both
cg02283643	Both
cg06539804	Both
cg17398595	Both
cg15127324	Both
cg23444894	Both
cg08536841	Both
cg14449051	Both
cg24362016	Both
cg13109289	Both
cg03860890	Both
cg22956483	Both
cg23534142	Both
cg03388193	Both
cg15792367	Both
cg23878206	Both
cg00514407	Both
cg22325572	Both
cg03826976	Both
cg12069309	Both
cg10106388	Both
cg25955816	Both
cg12711814	Both
cg10560038	Both
cg22633722	Both
cg18107827	Both
cg11879188	Both
cg01289103	Both
cg19343464	Both
cg21303011	Both
cg07297178	Both
cg18669381	Both
cg26240939	Both
cg21330703	Both
cg25095814	Both
cg10281478	Both
cg05778847	Both
cg11207379	Both
cg17055734	Both
cg05839235	Both
cg22619018	Both
cg01413516	Both
cg00436282	Both
cg20899321	Both
cg04609640	Both
cg15915418	Both
cg20657421	Both
cg09628601	Both
cg00350478	Both
cg01031251	Both
cg13878456	Both

cg15842430	Both
cg00333528	Both
cg20134215	Both
cg08424423	Both
cg27412093	Both
cg21448423	Both
cg00174500	Both
cg23843505	Both
cg21279601	Both
cg14345676	Both
cg23674788	Both
cg24027342	Both
cg18108623	Both
cg22341310	Both
cg11846968	Both
cg09089053	Both
cg15604467	Both
cg08397758	Both
cg15177917	Both
cg26675077	Both
cg17663463	Both
cg06825166	Both
cg00350702	Both
cg17923358	Both
cg13765785	Both
cg06736444	Both
cg21567332	Both
cg12840719	Both
cg06494782	Both
cg06971096	Both
cg04505023	Both
cg25141674	Both
cg03468463	Both
cg21033494	Both
cg22566906	Both
cg05087948	Both
cg21230133	Both
cg10981541	Both
cg15727249	Both
cg22243662	Both
cg16324018	Both
cg02536286	Both
cg03780486	Both
cg06458239	Both
cg19109050	Both
cg03922337	Both
cg08361238	Both
cg25136045	Both
cg13792279	Both
cg25650811	Both
cg05800321	Both
cg15089387	Both
cg11905488	Both

cg20862119	Both
cg00459975	Both
cg20625138	Both
cg03879730	Both
cg05338167	Both
cg20556988	Both
cg16584573	Both
cg03770147	Both
cg13540795	Both
cg15518950	Both
cg07054095	Both
cg10500909	Both
cg15001381	Both
cg22359606	Both
cg26507477	Both
cg23739862	Both
cg00718513	Both
cg00973677	Both
cg24353217	Both
cg09143663	Both
cg25664034	Both
cg17757055	Both
cg00230502	Both
cg20050113	Both
cg15852891	Both
cg12958778	Both
cg17977409	Both
cg16670497	Both
cg18983672	Both
cg22100821	Both
cg11919694	Both
cg16539629	Both
cg10056627	Both
cg27035169	Both
cg08108641	Both
cg26555310	Both
cg15647515	Both
cg13521229	Both
cg25033993	Both
cg01808508	Both
cg03013422	Both
cg16307860	Both
cg20775959	Both
cg02214188	Both
cg26511321	Both
cg24919972	Both
cg04561804	Both
cg14100184	Both
cg13914438	Both
cg11487705	Both
cg04856858	Both
cg03900284	Both
cg23047271	Both

cg11873854	Both
cg16016036	Both
cg16907488	Both
cg08023751	Both
cg23029193	Both
cg03475172	Both
cg17767931	Both
cg05895453	Both
cg22959932	Both
cg13446199	Both
cg03242880	Both
cg23857226	Both
cg26813458	Both
cg09871043	Both
cg06186861	Both
cg22337624	Both
cg05989054	Both
cg07112210	Both
cg06666486	Both
cg11081833	Both
cg18536148	Both
cg08433095	Both
cg09419900	Both
cg14076161	Both
cg13284426	Both
cg09190408	Both
cg05795313	Both
cg08003150	Both
cg10399228	Both
cg08016363	Both
cg23481748	Both
cg08670691	Both
cg21309147	Both
cg27360098	Both
cg14802951	Both
cg17694279	Both
cg25021247	Both
cg01369981	Both
cg08126211	Both
cg11471401	Both
cg06874144	Both
cg22025233	Both
cg02810134	Both
cg23283495	Both
cg11530960	Both
cg00412805	Both
cg06659073	Both
cg15134649	Both
cg19868730	Both
cg24438217	Both
cg05055150	Both
cg06465194	Both
cg26898336	Both

cg08343834	Both
cg15923513	Both
cg22496652	Both
cg20673075	Both
cg18841634	Both
cg18905252	Both
cg17770886	Both
cg08744726	Both
cg11719157	Both
cg16354207	Both
cg16256504	Both
cg17665193	Both
cg22807700	Both
cg02887841	Both
cg05112299	Both
cg02932167	Both
cg02883161	Both
cg27154163	Both
cg14694011	Both
cg01192952	Both
cg20576002	Both
cg24331079	Both
cg06866657	Both
cg24599942	Both
cg01485797	Both
cg08555772	Both
cg23587449	Both
cg03478689	Both
cg25149155	Both
cg11354906	Both
cg22757447	Both
cg22792432	Both
cg16773899	Both
cg08274234	Both
cg10293925	Both
cg15236866	Both
cg02430692	Both
cg22023770	Both
cg08789630	Both
cg25681177	Both
cg06392589	Both
cg25344672	Both
cg26777475	Both
cg08704509	Both
cg22088368	Both
cg01885635	Both
cg18958531	Both
cg06637893	Both
cg04926244	Both
cg00143998	Both
cg05982504	Both
cg24621354	Both
cg01697865	Both

cg16713727	Both
cg20006825	Both
cg05163057	Both
cg16703647	Both
cg07549194	Both
cg23106864	Both
cg03593419	Both
cg00079563	Both
cg11260422	Both
cg26847490	Both
cg08307963	Both
cg11887234	Both
cg04748704	Both
cg21361470	Both
cg02564523	Both
cg18634211	Both
cg12547930	Both
cg23509869	Both
cg16854606	Both
cg18462653	Both
cg22594309	Both
cg23114866	Both
cg06768707	Both
cg25076881	Both
cg13997068	Both
cg20287640	Both
cg07061913	Both
cg11885098	Both
cg21109025	Both
cg21182407	Both
cg03139377	Both
cg01259619	Both
cg24833176	Both
cg11822659	Both
cg08453096	Both
cg20161179	Both
cg14607642	Both
cg03977782	Both
cg13812587	Both
cg00228799	Both
cg00394658	Both
cg09747578	Both
cg22473973	Both
cg08364102	Both
cg17880199	Both
cg18275051	Both
cg01214847	Both
cg16267266	Both
cg12806521	Both
cg03021690	Both
cg00534274	Both
cg07664183	Both
cg16332224	Both

cg03565081	Both
cg17215680	Both
cg02584520	Both
cg10920957	Both
cg17915429	Both
cg26145228	Both
cg00626119	Both
cg19423311	Both
cg21047206	Both
cg11993754	Both
cg01078276	Both
cg12800028	Both
cg26657648	Both
cg05360265	Both
cg25736482	Both
cg17307280	Both
cg25239996	Both
cg03454353	Both
cg19109431	Both
cg22136753	Both
cg04060163	Both
cg19881895	Both
cg18913171	Both
cg01775414	Both
cg06821993	Both
cg23337754	Both
cg06738602	Both
cg15750705	Both
cg19345602	Both
cg26233914	Both
cg18750833	Both
cg14458731	Both
cg24133115	Both
cg14481339	Both
cg08920071	Both
cg11466837	Both
cg17133183	Both
cg00073650	Both
cg10612997	Both
cg07705908	Both
cg25214346	Both
cg06142324	Both
cg09155025	Both
cg25374854	Both
cg09480837	Both
cg15271616	Both
cg27236973	Both
cg17214107	Both
cg10241097	Both
cg09222791	Both
cg04592706	Both
cg04456238	Both
cg01211097	Both

cg26804057	Both
cg11507178	Both
cg17995823	Both
cg25021182	Both
cg22171829	Both
cg11630392	Both
cg18829411	Both
cg09691574	Both
cg25163476	Both
cg18565510	Both
cg22122449	Both
cg24183484	Both
cg16133244	Both
cg12796229	Both
cg22377998	Both
cg20200335	Both
cg04473302	Both
cg14472778	Both
cg00892393	Both
cg09229912	Both
cg25162301	Both
cg06954481	Both
cg00689010	Both
cg22430790	Both
cg17878972	Both
cg18910313	Both
cg20821095	Both
cg17421623	Both
cg13888886	Both
cg04189838	Both
cg24120841	Both
cg26420196	Both
cg03900104	Both
cg20028291	Both
cg22892110	Both
cg17470697	Both
cg19884600	Both
cg07251857	Both
cg08970446	Both
cg02296128	Both
cg17329164	Both
cg23881725	Both
cg02115041	Both
cg03682712	Both
cg08009711	Both
cg20884362	Both
cg03636183	Both
cg05861661	Both
cg04587910	Both
cg12435792	Both
cg14886269	Both
cg16507522	Both
cg00488300	Both

cg18320336	Both
cg15949277	Both
cg06517798	Both
cg07313155	Both
cg14436426	Both
cg11897314	Both
cg05044994	Both
cg10334385	Both
cg01731341	Both
cg16378421	Both
cg05292376	Both
cg07031996	Both
cg10501128	Both
cg10031651	Both
cg19427472	Both
cg26390526	Both
cg26134665	Both
cg19201019	Both
cg04882759	Both
cg13858139	Both
cg24355091	Both
cg13807496	Both
cg08404225	Both
cg01112778	Both
cg12643449	Both
cg15147435	Both
cg26767761	Both
cg04640913	Both
cg00648883	Both
cg04270799	Both
cg20994801	Both
cg16052901	Both
cg10918202	Both
cg06417962	Both
cg00615377	Both
cg19514469	Both
cg16558203	Both
cg16677885	Both
cg12195135	Both
cg02533173	Both
cg23154064	Both
cg27590397	Both
cg06851207	Both
cg24142106	Both
cg08411049	Both
cg17018527	Both
cg01958189	Both
cg00815605	Both
cg08331960	Both
cg17468440	Both
cg20987610	Both
cg17523380	Both
cg24244000	Both

cg15802898	Both
cg25550573	Both
cg17872757	Both
cg26174752	Both
cg09001953	Both
cg14269477	Both
cg20256783	Both
cg21825027	Both
cg12593411	Both
cg22319147	Both
cg20925811	Both
cg22441882	Both
cg25771201	Both
cg26912636	Both
cg15585987	Both
cg02089348	Both
cg25587233	Both
cg08108953	Both
cg17696091	Both
cg21274025	Both
cg13300756	Both
cg11237738	Both
cg10729531	Both
cg19777470	Both
cg17020834	Both
cg06148175	Both
cg12640109	Both
cg06607866	Both
cg20436912	Both
cg04482110	Both
cg01990304	Both
cg04713352	Both
cg16393207	Both
cg26809210	Both
cg01500140	Both
cg19875656	Both
cg22294577	Both
cg06229674	Both
cg15994159	Both
cg06933965	Both
cg17079378	Both
cg26937500	Both
cg18230771	Both
cg20229788	Both
cg07092725	Both
cg18489434	Both
cg25890048	Both
cg19521927	Both
cg07175883	Both
cg00239685	Both
cg18197795	Both
cg08833432	Both
cg22045288	Both

cg05517572	Both
cg00563926	Both
cg25674286	Both
cg02097636	Both
cg11647681	Both
cg21885995	Both
cg26578617	Both
cg23582408	Both
cg26196700	Both
cg24594997	Both
cg00053292	Both
cg08044694	Both
cg21787206	Both
cg25093045	Both
cg24461814	Both
cg11953824	Both
cg08901867	Both
cg21051086	Both
cg17832674	Both
cg01027739	Both
cg15526708	Both
cg07057831	Both
cg00229387	Both
cg19096034	Both
cg01722994	Both
cg21633208	Both
cg13494498	Both
cg23769143	Both
cg13828047	Both
cg02475653	Both
cg02194211	Both
cg22830895	Both
cg11879577	Both
cg21859434	Both
cg09414535	Both
cg17498321	Both
cg00828602	Both
cg03760483	Both
cg27190239	Both
cg03548857	Both
cg21210789	Both
cg09191327	Both
cg01607495	Both
cg10108208	Both
cg11688469	Both
cg00684178	Both
cg16509658	Both
cg15819171	Both
cg24441350	Both
cg01226811	Both
cg18279742	Both
cg12998614	Both
cg05498681	Both

cg15486645	Both
cg10954182	Both
cg04711324	Both
cg27169020	Both
cg16790239	Both
cg13631259	Both
cg25820971	Both
cg19430430	Both
cg04518808	Both
cg15774283	Both
cg15827031	Both
cg27478659	Both
cg26521404	Both
cg22898761	Both
cg25867173	Both
cg05088512	Both
cg00134539	Both
cg02713563	Both
cg12120741	Both
cg04845063	Both
cg26763524	Both
cg04005707	Both
cg23276695	Both
cg21432954	Both
cg05881135	Both
cg06975499	Both
cg08996748	Both
cg01184449	Both
cg25228126	Both
cg26205432	Both
cg13543096	Both
cg04409427	Both
cg00365193	Both
cg24443367	Both
cg20227165	Both
cg00970325	Both
cg15799267	Both
cg05163071	Both
cg06810461	Both
cg10213812	Both
cg06459066	Both
cg00466249	Both
cg16893614	Both
cg17414431	Both
cg22747092	Both
cg08634024	Both
cg11896271	Both
cg18470891	Both
cg21094154	Both
cg19485804	Both
cg14611174	Both
cg14818279	Both
cg22901840	Both

cg27601582	Both
cg02624705	Both
cg17001430	Both
cg23656380	Both
cg07066326	Both
cg19421752	Both
cg10187932	Both
cg05890019	Both
cg03513363	Both
cg05063104	Both
cg24855780	Both
cg02298570	Both
cg08655844	Both
cg22762180	Both
cg10063179	Both
cg02630207	Both
cg24477636	Both
cg10741760	Both
cg01441777	Both
cg18085435	Both
cg03172991	Both
cg17398003	Both
cg10335931	Both
cg05823029	Both
cg05657520	Both
cg27552599	Both
cg16627949	Both
cg08594695	Both
cg04356968	Both
cg16557944	Both
cg17644208	Both
cg11698653	Both
cg03264414	Both
cg21842478	Both
cg21731286	Both
cg21116314	Both
cg18546622	Both
cg09607282	Both
cg04057053	Both
cg25437385	Both
cg12571423	Both
cg01971122	Both
cg00169548	Both
cg23915111	Both
cg15918284	Both
cg27419217	Both
cg04698187	Both
cg02762689	Both
cg07974303	Both
cg14360917	Both
cg21581873	Both
cg25221254	Both
cg06746101	Both

cg16574065	Both
cg19482581	Both
cg00832994	Both
cg07785936	Both
cg19560758	Both
cg09622447	Both
cg25884854	Both
cg26352374	Both
cg18503260	Both
cg04039397	Both
cg22429822	Both
cg15836722	Both
cg24580001	Both
cg01939428	Both
cg15234096	Both
cg07103819	Both
cg04375036	Both
cg14753432	Both
cg21509097	Both
cg21922574	Both
cg21922841	Both
cg11344614	Both
cg16929104	Both
cg14896003	Both
cg07690018	Both
cg14391622	Both
cg12902039	Both
cg15910208	Both
cg23665603	Both
cg13896269	Both
cg26536259	Both
cg04066242	Both
cg20185083	Both
cg00080012	Both
cg13928961	Both
cg18652941	Both
cg15686608	Both
cg13315147	Both
cg00083720	Both
cg20016416	Both
cg21418076	Both
cg21570220	Both
cg10503138	Both
cg09107315	Both
cg06110365	Both
cg26538442	Both
cg01465325	Both
cg07548313	Both
cg04590978	Both
cg10099900	Both
cg17711541	Both
cg19399532	Both
cg19674669	Both

cg22063989	Both
cg24674220	Both
cg27217148	Both
cg23003881	Both
cg14912034	Both
cg04765277	Both
cg07758904	Both
cg10523671	Both
cg24898863	Both
cg22396755	Both
cg08069899	Both
cg19771589	Both
cg03003256	Both
cg21948655	Both
cg09640202	Both
cg24619694	Both
cg27486427	Both
cg18982568	Both
cg10748867	Both
cg17119387	Both
cg05649009	Both
cg21349901	Both
cg14541311	Both
cg17660655	Both
cg04113075	Both
cg23519969	Both
cg04201347	Both
cg02590345	Both
cg10775273	Both
cg11695266	Both
cg22512531	Both
cg21256649	Both
cg19778698	Both
cg21084260	Both
cg22724153	Both
cg05953243	Both
cg15364618	Both
cg10313633	Both
cg19320612	Both
cg26251865	Both
cg04368877	Both
cg23051598	Both
cg01130192	Both
cg15747933	Both
cg02449608	Both
cg13320683	Both
cg19969873	Both
cg06856528	Both
cg08528984	Both
cg04828792	Both
cg05508558	Both
cg01962086	Both
cg06367117	Both

cg21438018	Both
cg03222066	Both
cg20938359	Both
cg07423149	Both
cg16862361	Both
cg19784470	Both
cg07015629	Both
cg25979644	Both
cg14667273	Both
cg27494383	Both
cg01968178	Both
cg19658284	Both
cg10301967	Both
cg01169726	Both
cg12445208	Both
cg10730174	Both
cg06883149	Both
cg16998872	Both
cg01525376	Both
cg17543123	Both
cg12234947	Both
cg08771731	Both
cg14087150	Both
cg05485062	Both
cg27378216	Both
cg09911342	Both
cg27050793	Both
cg22409383	Both
cg02717570	Both
cg14920334	Both
cg15227610	Both
cg20520725	Both
cg11406340	Both
cg04514469	Both
cg23967169	Both
cg12894984	Both
cg07376029	Both
cg01805540	Both
cg20888386	Both
cg00019495	Both
cg08530414	Both
cg07314549	Both
cg26615126	Both
cg08355340	Both
cg05159732	Both
cg16869108	Both
cg27635271	Both
cg15684563	Both
cg18219418	Both
cg03133269	Both
cg01386493	Both
cg08539093	Both
cg17197538	Both

cg19177941	Both
cg07850604	Both
cg04820387	Both
cg05189291	Both
cg24006361	Both
cg12884406	Both
cg15984661	Both
cg09088834	Both
cg09251995	Both
cg02194878	Both
cg24068708	Both
cg26360732	Both
cg11981631	Both
cg15301694	Both
cg24623271	Both
cg05877109	Both
cg12687990	Both
cg09196959	Both
cg03803009	Both
cg17928268	Both
cg06207804	Both
cg12497564	Both
cg09163021	Both
cg07962315	Both
cg03668539	Both
cg16005224	Both
cg11037787	Both
cg07971188	Both
cg12467090	Both
cg11536940	Both
cg19570317	Both
cg14436761	Both
cg10238171	Both
cg08742106	Both
cg04584523	Both
cg02212836	Both
cg04296434	Both
cg00772000	Both
cg05500074	Both
cg26014796	Both
cg10490064	Both
cg00141162	Both
cg13560548	Both
cg10946435	Both
cg07080358	Both
cg09628707	Both
cg17602451	Both
cg24968786	Both
cg00850538	Both
cg25404088	Both
cg03920233	Both
cg17496788	Both
cg25361106	Both

cg21504918	Both
cg25383093	Both
cg23530850	Both
cg02376163	Both
cg02579133	Both
cg11777321	Both
cg13425637	Both
cg09386615	Both
cg05226008	Both
cg16158681	Both
cg14586939	Both
cg15109207	Both
cg09155905	Both
cg15817236	Both
cg19358493	Both
cg19554294	Both
cg22886089	Both
cg26331247	Both
cg27076046	Both
cg18055007	Both
cg25047280	Both
cg00495491	Both
cg24691453	Both
cg01568736	Both
cg10011232	Both
cg03673470	Both
cg10726357	Both
cg25434223	Both
cg11220060	Both
cg07478122	Both
cg26956535	Both
cg02087637	Both
cg21633698	Both
cg05360220	Both
cg25599211	Both
cg04979933	Both
cg06222800	Both
cg07099407	Both
cg27650434	Both
cg07236190	Both
cg08045570	Both
cg11462865	Both
cg17714828	Both
cg06790862	Both
cg08647727	Both
cg06238491	Both
cg10377274	Both
cg26985289	Both
cg27442349	Both
cg19447496	Both
cg00614413	Both
cg01920232	Both
cg17561452	Both

cg17884373	Both
cg27214365	Both
cg06720660	Both
cg01663469	Both
cg19466563	Both
cg21460582	Both
cg19463518	Both
cg22543128	Both
cg15798455	Both
cg09896445	Both
cg08557686	Both
cg26499286	Both
cg15303841	Both
cg04037952	Both
cg05294455	Both
cg09231514	Both
cg20736065	Both
cg12073779	Both
cg04219321	Both
cg04677163	Both
cg02790471	Both
cg01942127	Both
cg11115702	Both
cg01346718	Both
cg08132711	Both
cg26212229	Both
cg18411898	Both
cg27117399	Both
cg18396533	Both
cg23752923	Both
cg04502814	Both
cg08837884	Both
cg27626102	Both
cg11394785	Both
cg13474734	Both
cg11479877	Both
cg13669740	Both
cg14858551	Both
cg03139057	Both
cg26776077	Both
cg06742978	Both
cg23507131	Both
cg22412741	Both
cg07163603	Both
cg14592099	Both
cg09390792	Both
cg15698196	Both
cg20586531	Both
cg05358291	Both
cg16479674	Both
cg24411312	Both
cg25536676	Both
cg15702701	Both

cg11248413	Both
cg20406878	Both
cg12238343	Both
cg17727529	Both
cg27480700	Both
cg17133388	Both
cg14743462	Both
cg02512860	Both
cg25416372	Both
cg15453943	Both
cg10938446	Both
cg14930674	Both
cg14913925	Both
cg15062535	Both
cg17465219	Both
cg06230736	Both
cg23941599	Both
cg18236079	Both
cg08380205	Both
cg13493001	Both
cg00754617	Both
cg01086895	Both
cg25168545	Both
cg05781767	Both
cg04862249	Both
cg16994506	Both
cg18771300	Both
cg15809230	Both
cg27398547	Both
cg25450806	Both
cg10585962	Both
cg12024292	Both
cg09736162	Both
cg12317456	Both
cg13470920	Both
cg25501446	Both
cg26164310	Both
cg13864937	Both
cg18806980	Both
cg01422337	Both
cg06942110	Both
cg12274567	Both
cg19626078	Both
cg08743392	Both
cg19921389	Both
cg08936952	Both
cg23547429	Both
cg27138018	Both
cg24321030	Both
cg11739626	Both
cg21187265	Both
cg16796951	Both
cg24912023	Both

cg22375610	Both
cg03513163	Both
cg01910481	Both
cg01661993	Both
cg20955688	Both
cg06500079	Both
cg22286764	Both
cg17838626	Both
cg19685066	Both
cg21621204	Both
cg09532664	Both
cg24116028	Both
cg14059963	Both
cg23412850	Both
cg12532169	Both
cg22647018	Both
cg24363955	Both
cg17804302	Both
cg07039113	Both
cg17671157	Both
cg17803430	Both
cg00745543	Both
cg24030630	Both
cg09701102	Both
cg08696192	Both
cg21387281	Both
cg11059341	Both
cg22467071	Both
cg11122968	Both
cg02641676	Both
cg10219037	Both
cg18883209	Both
cg06785822	Both
cg14023451	Both
cg11277230	Both
cg20395072	Both
cg16003238	Both
cg04599297	Both
cg02351381	Both
cg00711916	Both
cg16542081	Both
cg12846938	Both
cg00750606	Both
cg19098268	Both
cg22051636	Both
cg16739580	Both
cg24300924	Both
cg24654350	Both
cg11151665	Both
cg14435807	Both
cg22065439	Both
cg00935388	Both
cg00406844	Both

cg09479015	Both
cg21801378	Both
cg12893143	Both
cg03626672	Both
cg01657207	Both
cg02902770	Both
cg26624118	Both
cg24975842	Both
cg01154193	Both
cg16620731	Both
cg26764244	Both
cg12118437	Both
cg09847584	Both
cg12820481	Both
cg04557383	Both
cg12856392	Both
cg11553177	Both
cg00067471	Both
cg03931808	Both
cg21611708	Both
cg18239253	Both
cg23097681	Both
cg03101664	Both
cg20029652	Both
cg09773756	Both
cg02100629	Both
cg13840968	Both
cg25870263	Both
cg12181621	Both
cg23293787	Both
cg19623751	Both
cg26817382	Both
cg20002248	Both
cg08514736	Both
cg10605520	Both
cg27462160	Both
cg25002911	Both
cg05020203	Both
cg03096975	Both
cg11297236	Both
cg20259398	Both
cg21264055	Both
cg25218351	Both
cg16005443	Both
cg20225681	Both
cg03583857	Both
cg02873524	Both
cg01737507	Both
cg19850370	Both
cg11608424	Both
cg04032226	Both
cg12408911	Both
cg08555612	Both

cg16029760	Both
cg04463638	Both
cg03014680	Both
cg24401441	Both
cg14384940	Both
cg04180953	Both
cg03998348	Both
cg07056057	Both
cg09354331	Both
cg12067287	Both
cg12113132	Both
cg24100167	Both
cg00877887	Both
cg11784785	Both
cg03591238	Both
cg22642718	Both
cg01796228	Both
cg15582891	Both
cg00261552	Both
cg14213992	Both
cg24477567	Both
cg07196761	Both
cg25989745	Both
cg10189763	Both
cg19761273	Both
cg16270890	Both
cg15589427	Both
cg24273512	Both
cg22593785	Both
cg03819692	Both
cg06638966	Both
cg06641503	Both
cg14696348	Both
cg04995095	Both
cg22459146	Both
cg07715894	Both
cg10163825	Both
cg00512031	Both
cg04739570	Both
cg22037121	Both
cg14451276	Both
cg22689690	Both
cg16388829	Both
cg00679556	Both
cg03969997	Both
cg26354398	Both
cg10002103	Both
cg14448145	Both
cg26055770	Both
cg02501779	Both
cg20732367	Both
cg02548238	Both
cg01656955	Both

cg01536400	Both
cg02658214	Both
cg07623294	Both
cg17430393	Both
cg07592353	Both
cg16001913	Both
cg20224517	Both
cg15170903	Both
cg05556202	Both
cg03851112	Both
cg00234961	Both
cg04806409	Both
cg21123573	Both
cg12811135	Both
cg18096388	Both
cg26713220	Both
cg04388983	Both
cg05535113	Both
cg25832796	Both
cg04478795	Both
cg10878998	Both
cg13986130	Both
cg11504739	Both
cg00340102	Both
cg20143092	Both
cg27226618	Both
cg02992596	Both
cg25760229	Both
cg04536922	Both
cg10893437	Both
cg02516189	Both
cg27210447	Both
cg26502489	Both
cg26491213	Both
cg05501682	Both
cg26815414	Both
cg13460409	Both
cg06433658	Both
cg09542745	Both
cg17682828	Both
cg05341878	Both
cg11599505	Both
cg07660236	Both
cg23767977	Both
cg07747299	Both
cg24781100	Both
cg12962778	Both
cg16652259	Both
cg21604803	Both
cg23287547	Both
cg27404050	Both
cg09547224	Both
cg18142353	Both

cg24530795	Both
cg09113530	Both
cg12539975	Both
cg03020951	Both
cg10541755	Both
cg20379125	Both
cg18338311	Both
cg21514871	Both
cg05705583	Both
cg21930712	Both
cg10722799	Both
cg18638931	Both
cg01614759	Both
cg16607065	Both
cg25697314	Both
cg19728223	Both
cg21663722	Both
cg20699736	Both
cg18640030	Both
cg02677802	Both
cg06827976	Both
cg24824840	Both
cg12473775	Both
cg24319545	Both
cg00725635	Both
cg03848675	Both
cg24500683	Both
cg00016968	Both
cg11747771	Both
cg06385087	Both
cg16879115	Both
cg19629292	Both
cg23183296	Both
cg08385610	Both
cg06994793	Both
cg13912117	Both
cg01136458	Both
cg08917718	Both
cg19224278	Both
cg15508020	Both
cg27535305	Both
cg07514381	Both
cg08365982	Both
cg14547335	Both
cg19497444	Both
cg02497428	Both
cg05681757	Both
cg23744433	Both
cg25739003	Both
cg15127806	Both
cg10839997	Both
cg17338403	Both
cg22125370	Both

cg20595215	Both
cg25900806	Both
cg05932408	Both
cg06141025	Both
cg16411152	Both
cg05932936	Both
cg25103286	Both
cg15448245	Both
cg13589108	Both
cg19324313	Both
cg22772878	Both
cg12624523	Both
cg22938407	Both
cg08191854	Both
cg13531460	Both
cg02189785	Both
cg18888520	Both
cg18453621	Both
cg24653181	Both
cg00826384	Both
cg25042226	Both
cg04902405	Both
cg20579480	Both
cg25595446	Both
cg20795863	Both
cg13726191	Both
cg01920829	Both
cg26917999	Both
cg08687825	Both
cg03597525	Both
cg26607785	Both
cg18873386	Both
cg07427065	Both
cg24101578	Both
cg02798801	Both
cg12815916	Both
cg14920846	Both
cg04525773	Both
cg24231716	Both
cg20632573	Both
cg16003913	Both
cg15812873	Both
cg07233761	Both
cg24516061	Both
cg27554782	Both
cg06459327	Both
cg04377282	Both
cg14587868	Both
cg25870420	Both
cg24075743	Both
cg14849423	Both
cg23639989	Both
cg26755793	Both

cg23970523	Both
cg27495908	Both
cg03138668	Both
cg26045220	Both
cg01125463	Both
cg13571802	Both
cg10539808	Both
cg01173186	Both
cg10356060	Both
cg20740029	Both
cg16567044	Both
cg02941816	Both
cg13301003	Both
cg24886748	Both
cg24997944	Both
cg05365525	Both
cg25107903	Both
cg19896198	Both
cg01539186	Both
cg10530281	Both
cg14870461	Both
cg07393322	Both
cg06602857	Both
cg15269875	Both
cg13789236	Both
cg16772207	Both
cg17705056	Both
cg03714916	Both
cg09748960	Both
cg08162780	Both
cg27147004	Both
cg04270835	Both
cg13633560	Both
cg18881723	Both
cg01390445	Both
cg25201363	Both
cg00293409	Both
cg23058901	Both
cg05647526	Both
cg17185543	Both
cg21709140	Both
cg11732619	Both
cg06781209	Both
cg07123548	Both
cg04484789	Both
cg08458170	Both
cg04283938	Both
cg14776962	Both
cg00679738	Both
cg13080465	Both
cg02318535	Both
cg14580982	Both
cg12089439	Both

cg17122311	Both
cg20530314	Both
cg00615241	Both
cg24625128	Both
cg07074571	Both
cg16911220	Both
cg12880200	Both
cg13599477	Both
cg03243506	Both
cg10316635	Both
cg05621401	Both
cg22496859	Both
cg18618334	Both
cg23091824	Both
cg09553448	Both
cg02131995	Both
cg14826683	Both
cg14896516	Both
cg08475088	Both
cg10784030	Both
cg06630737	Both
cg08009622	Both
cg15003158	Both
cg13770446	Both
cg14893163	Both
cg09360654	Both
cg02136132	Both
cg13030582	Both
cg00657095	Both
cg07884474	Both
cg02569613	Both
cg19033555	Both
cg14321743	Both
cg14792155	Both
cg11721194	Both
cg17522249	Both
cg18787783	Both
cg16762386	Both
cg10871523	Both
cg10039928	Both
cg07285276	Both
cg02014107	Both
cg15068733	Both
cg00239071	Both
cg06277481	Both
cg22932819	Both
cg02658251	Both
cg03620376	Both
cg26379475	Both
cg00321478	Both
cg08025786	Both
cg24719601	Both
cg15125424	Both

cg24562819	Both
cg19692710	Both
cg21353232	Both
cg14141399	Both
cg25665528	Both
cg15035273	Both
cg13650156	Both
cg14701962	Both
cg08896945	Both
cg07773642	Both
cg15712267	Both
cg26215428	Both
cg02998425	Both
cg06397510	Both
cg24276445	Both
cg21457147	Both
cg02058918	Both
cg16245844	Both
cg13535217	Both
cg11070941	Both
cg04532952	Both
cg24555600	Both
cg20692181	Both
cg23239039	Both
cg20422318	Both
cg09874752	Both
cg03270204	Both
cg13688966	Both
cg05782292	Both
cg22680812	Both
cg20176989	Both
cg08897388	Both
cg26105232	Both
cg23144899	Both
cg13055278	Both
cg11747499	Both
cg21529533	Both
cg06799664	Both
cg13263483	Both
cg16004226	Both
cg24283842	Both
cg19615614	Both
cg02633817	Both
cg08088989	Both
cg09565688	Both
cg16176379	Both
cg06063714	Both
cg10743104	Both
cg06297145	Both
cg20346726	Both
cg12970008	Both
cg26588422	Both
cg00565075	Both

cg19044630	Both
cg11511443	Both
cg11395610	Both
cg04315771	Both
cg24407308	Both
cg02034222	Both
cg02812142	Both
cg18988395	Both
cg22854223	Both
cg00083937	Both
cg26035366	Both
cg27256309	Both
cg02130905	Both
cg25935911	Both
cg26259363	Both
cg25013053	Both
cg10481417	Both
cg08035942	Both
cg05434957	Both
cg14086122	Both
cg00747849	Both
cg08089301	Both
cg18508525	Both
cg02806658	Both
cg02163937	Both
cg13565157	Both
cg26646370	Both
cg04457794	Both
cg19224837	Both
cg16713808	Both
cg23741330	Both
cg18344063	Both
cg09936762	Both
cg01656470	Both
cg02955988	Both
cg13099330	Both
cg26752657	Both
cg16256643	Both
cg02092466	Both
cg16797831	Both
cg03029616	Both
cg04677227	Both
cg03684977	Both
cg17243643	Both
cg07485777	Both
cg18403361	Both
cg18466173	Both
cg25174591	Both
cg11119131	Both
cg09906488	Both
cg21045388	Both
cg27413508	Both
cg00587613	Both

cg26922202	Both
cg05348272	Both
cg19812619	Both
cg18943383	Both
cg06243556	Both
cg00392257	Both
cg18117847	Both
cg23713520	Both
cg04775502	Both
cg11504740	Both
cg27182551	Both
cg17906786	Both
cg14758413	Both
cg19394737	Both
cg14003512	Both
cg01432087	Both
cg09799873	Both
cg12770741	Both
cg09538287	Both
cg17693957	Both
cg17226343	Both
cg07862358	Both
cg05825950	Both
cg09636671	Both
cg17407908	Both
cg16793061	Both
cg16148403	Both
cg26473272	Both
cg06391468	Both
cg13605579	Both
cg19147390	Both
cg15536490	Both
cg16998353	Both
cg00930194	Both
cg03277112	Both
cg06378617	Both
cg16886259	Both
cg02661879	Both
cg10971269	Both
cg26738010	Both
cg19839691	Both
cg22737001	Both
cg03854071	Both
cg24354652	Both
cg22175764	Both
cg14726525	Both
cg03026462	Both
cg05105913	Both
cg15310873	Both
cg16359129	Both
cg20768743	Both
cg13258700	Both
cg23599843	Both

cg17866455	Both
cg21166999	Both
cg04034767	Both
cg08123074	Both
cg21144270	Both
cg17252960	Both
cg27557796	Both
cg06688396	Both
cg03462055	Both
cg24889366	Both
cg20361540	Both
cg01655355	Both
cg17558126	Both
cg16193203	Both
cg26350691	Both
cg12205230	Both
cg07300408	Both
cg11314271	Both
cg11181795	Both
cg12188538	Both
cg09881855	Both
cg06614002	Both
cg02647265	Both
cg03135127	Both
cg12432709	Both
cg17639046	Both
cg01772980	Both
cg14171523	Both
cg27341860	Both
cg26822241	Both
cg10575841	Both
cg23749760	Both
cg24659201	Both
cg13060997	Both
cg16028753	Both
cg13842648	Both
cg08768421	Both
cg00077877	Both
cg13255629	Both
cg00233307	Both
cg09422355	Both
cg26723847	Both
cg26238727	Both
cg04697562	Both
cg00650762	Both
cg21279955	Both
cg14598387	Both
cg18848394	Both
cg04011872	Both
cg11003133	Both
cg06444781	Both
cg10557828	Both
cg12513481	Both

cg14409958	Both
cg11510839	Both
cg07679836	Both
cg09033997	Both
cg09991975	Both
cg06206628	Both
cg15133917	Both
cg09547777	Both
cg22879515	Both
cg09522147	Both
cg14869028	Both
cg13042288	Both
cg06812033	Both
cg08258650	Both
cg26783856	Both
cg09846458	Both
cg21306775	Both
cg09963123	Both
cg20273774	Both
cg21375825	Both
cg10500716	Both
cg22194129	Both
cg17536848	Both
cg13823701	Both
cg25270252	Both
cg17925542	Both
cg01172735	Both
cg15028436	Both
cg08212685	Both
cg04464446	Both
cg08132931	Both
cg16148454	Both
cg19947621	Both
cg17186803	Both
cg16220183	Both
cg13302154	Both
cg08961832	Both
cg15652212	Both
cg06594008	Both
cg18896687	Both
cg01280080	Both
cg19229991	Both
cg24560809	Both
cg21918500	Both
cg14401837	Both
cg26081162	Both
cg06726207	Both
cg13817266	Both
cg05961212	Both
cg04386405	Both
cg06282059	Both
cg11827101	Both
cg23616741	Both

cg08268266	Both
cg07567107	Both
cg00010193	Both
cg14417329	Both
cg19298821	Both
cg04443324	Both
cg15160445	Both
cg24605304	Both
cg12439899	Both
cg23239444	Both
cg12365667	Both
cg02007844	Both
cg17525406	Both
cg08859916	Both
cg27382389	Both
cg06281795	Both
cg24936956	Both
cg14221831	Both
cg09467501	Both
cg26637901	Both
cg18862260	Both
cg22193702	Both
cg24384676	Both
cg00546491	Both
cg04125208	Both
cg12124478	Both
cg08782122	Both
cg15977816	Both
cg13035743	Both
cg24134767	Both
cg14440934	Both
cg15914863	Both
cg04041960	Both
cg02488871	Both
cg14386312	Both
cg10840135	Both
cg20673481	Both
cg25306927	Both
cg06069187	Both
cg27037648	Both
cg17482740	Both
cg13043509	Both
cg19728577	Both
cg01618851	Both
cg16222568	Both
cg10957151	Both
cg25982743	Both
cg24076830	Both
cg04940435	Both
cg09983885	Both
cg05589246	Both
cg20761322	Both
cg18581445	Both

cg27546237	Both
cg06200697	Both
cg02248540	Both
cg01560871	Both
cg12594641	Both
cg00971396	Both
cg01293143	Both
cg11953334	Both
cg10669058	Both
cg25374813	Both
cg01982833	Both
cg01798589	Both
cg10719920	Both
cg22016649	Both
cg20011352	Both
cg21771250	Both
cg08211091	Both
cg15452573	Both
cg20269537	Both
cg13247990	Both
cg00033516	Both
cg26702254	Both
cg02630122	Both
cg11523020	Both
cg13603551	Both
cg13656062	Both
cg15188688	Both
cg07331725	Both
cg17960516	Both
cg04975920	Both
cg25574024	Both
cg24777454	Both
cg14662172	Both
cg16418163	Both
cg24341129	Both
cg03940103	Both
cg00116838	Both
cg15586352	Both
cg02859934	Both
cg01837719	Both
cg19157069	Both
cg27278802	Both
cg15563382	Both
cg22197830	Both
cg08981777	Both
cg13663218	Both
cg00689014	Both
cg09037813	Both
cg21146268	Both
cg08049198	Both
cg04597449	Both
cg17118262	Both
cg01777397	Both

cg00514895	Both
cg21624359	Both
cg21218093	Both
cg06746171	Both
cg12339029	Both
cg26354238	Both
cg12962021	Both
cg25753817	Both
cg18959478	Both
cg01899253	Both
cg06784339	Both
cg16444968	Both
cg04559909	Both
cg10846922	Both
cg05992340	Both
cg04527989	Both
cg26614346	Both
cg03264209	Both
cg25397076	Both
cg22108265	Both
cg24490338	Both
cg16564824	Both
cg17229388	Both
cg14924278	Both
cg17685628	Both
cg19026260	Both
cg12388309	Both
cg14892768	Both
cg19786920	Both
cg01791232	Both
cg02914379	Both
cg26831415	Both
cg27238470	Both
cg10977115	Both
cg14343711	Both
cg12172720	Both
cg09454892	Both
cg09737668	Both
cg00657582	Both
cg27472295	Both
cg07576541	Both
cg22294908	Both
cg20415809	Both
cg16617137	Both
cg22035229	Both
cg14021073	Both
cg08538752	Both
cg15721424	Both
cg04645174	Both
cg13744194	Both
cg00901766	Both
cg11177404	Both
cg17518962	Both

cg09712527	Both
cg22510412	Both
cg13966710	Both
cg07181349	Both
cg04726446	Both
cg21596858	Both
cg20345446	Both
cg24365013	Both
cg23061578	Both
cg22915732	Both
cg17298704	Both
cg13755023	Both
cg14345882	Both
cg07570142	Both
cg11905589	Both
cg24691255	Both
cg19180541	Both
cg00221494	Both
cg06744574	Both
cg14893161	Both
cg24266238	Both
cg08631151	Both
cg04103317	Both
cg25763788	Both
cg13765303	Both
cg07441272	Both
cg23322112	Both
cg23959705	Both
cg25345738	Both
cg05104081	Both
cg01581111	Both
cg14144305	Both
cg10615091	Both
cg19817399	Both
cg26104206	Both
cg03641225	Both
cg21652012	Both
cg20893022	Both
cg15423862	Both
cg01911082	Both
cg18108237	Both
cg12718562	Both
cg03773789	Both
cg16718891	Both
cg18931750	Both
cg15477600	Both
cg12215675	Both
cg11715999	Both
cg14672994	Both
cg21066636	Both
cg06836849	Both
cg00342530	Both
cg11834681	Both

cg26927807	Both
cg14473145	Both
cg26001336	Both
cg23213217	Both
cg10994430	Both
cg27277403	Both
cg11722531	Both
cg04471507	Both
cg25477904	Both
cg10074409	Both
cg22920873	Both
cg00295325	Both
cg11977634	Both
cg16358826	Both
cg06317209	Both
cg15212440	Both
cg04123409	Both
cg02901679	Both
cg07958192	Both
cg11158440	Both
cg24200059	Both
cg13118849	Both
cg08319991	Both
cg18292711	Both
cg18693704	Both
cg02729303	Both
cg20022541	Both
cg22315542	Both
cg24824266	Both
cg13216057	Both
cg09886641	Both
cg07607462	Both
cg13406950	Both
cg03944089	Both
cg10234985	Both
cg08341874	Both
cg14696820	Both
cg01021485	Both
cg03149130	Both
cg24448259	Both
cg09954385	Both
cg15983005	Both
cg12559031	Both
cg27181079	Both
cg17321617	Both
cg02477931	Both
cg00756887	Both
cg22722822	Both
cg16870320	Both
cg19042950	Both
cg15945754	Both
cg07892051	Both
cg10549973	Both

cg14654926	Both
cg17001035	Both
cg26187237	Both
cg10087399	Both
cg13555884	Both
cg15414833	Both
cg24116886	Both
cg12006284	Both
cg06495347	Both
cg26195812	Both
cg18938150	Both
cg15407570	Both
cg00819310	Both
cg05898102	Both
cg14322224	Both
cg03517000	Both
cg03017475	Both
cg09065199	Both
cg07952391	Both
cg06423920	Both
cg19987219	Both
cg04413148	Both
cg01200060	Both
cg02978737	Both
cg18011401	Both
cg07258507	Both
cg04030848	Both
cg21195414	Both
cg20857253	Both
cg25545210	Both
cg05323436	Both
cg19841506	Both
cg07141002	Both
cg07149772	Both
cg09495977	Both
cg11096993	Both
cg19863003	Both
cg03558807	Both
cg17769158	Both
cg12949975	Both
cg00622677	Both
cg17563769	Both
cg08328671	Both
cg22268231	Both
cg09553358	Both
cg00247489	Both
cg00554702	Both
cg18604842	Both
cg10347418	Both
cg11304734	Both
cg25072179	Both
cg09644722	Both
cg13313036	Both

cg26061555	Both
cg15317267	Both
cg18655915	Both
cg10414058	Both
cg24580782	Both
cg14799446	Both
cg24202119	Both
cg19953406	Both
cg02207610	Both
cg05001145	Both
cg09375488	Both
cg22692158	Both
cg02799466	Both
cg09915099	Both
cg14845091	Both
cg23504707	Both
cg21101222	Both
cg06952236	Both
cg24801210	Both
cg03430067	Both
cg21098323	Both
cg15120925	Both
cg13228642	Both
cg13644052	Both
cg23254045	Both
cg02327719	Both
cg09911755	Both
cg26753512	Both
cg24523000	Both
cg11155265	Both
cg18133966	Both
cg09494188	Both
cg01443067	Both
cg19016517	Both
cg25577842	Both
cg03118854	Both
cg02166532	Both
cg15767406	Both
cg09923671	Both
cg09453312	Both
cg08296824	Both
cg13968390	Both
cg23234999	Both
cg24678429	Both
cg14378480	Both
cg13144783	Both
cg09395732	Both
cg01428361	Both
cg20880234	Both
cg09786221	Both
cg15379237	Both
cg20751395	Both
cg02720618	Both

cg23143093	Both
cg02324920	Both
cg01429391	Both
cg03717979	Both
cg07880715	Both
cg13742532	Both
cg01684901	Both
cg20022122	Both
cg00969446	Both
cg16792632	Both
cg08169325	Both
cg14236602	Both
cg09173897	Both
cg23207527	Both
cg00622552	Both
cg01346152	Both
cg14060836	Both
cg12738197	Both
cg05835416	Both
cg01459162	Both
cg24389347	Both
cg05443740	Both
cg00354258	Both
cg10334928	Both
cg05372736	Both
cg15426734	Both
cg24127989	Both
cg13796218	Both
cg12970724	Both
cg15055101	Both
cg10876928	Both
cg23996306	Both
cg19986872	Both
cg24456340	Both
cg26258845	Both
cg06668073	Both
cg23660082	Both
cg09730349	Both
cg03633120	Both
cg12104707	Both
cg08797194	Both
cg20225275	Both
cg03237153	Both
cg14374842	Both
cg26548883	Both
cg23256150	Both
cg10643489	Both
cg02807948	Both
cg14823162	Both
cg25682080	Both
cg24192663	Both
cg14550066	Both
cg07599644	Both

cg04731384	Both
cg10917619	Both
cg03334529	Both
cg08843492	Both
cg23123262	Both
cg00134787	Both
cg15880738	Both
cg20642765	Both
cg20867633	Both
cg15768203	Both
cg27276456	Both
cg06958829	Both
cg19369556	Both
cg27133864	Both
cg18292394	Both
cg03382797	Both
cg25908985	Both
cg10025865	Both
cg10573763	Both
cg25124030	Both
cg12556134	Both
cg22879289	Both
cg12532477	Both
cg12529228	Both
cg06392241	Both
cg24977027	Both
cg09736922	Both
cg12019109	Both
cg08933517	Both
cg10995925	Both
cg00912942	Both
cg08190291	Both
cg09712066	Both
cg13944141	Both
cg09152089	Both
cg21572897	Both
cg04451770	Both
cg11158430	Both
cg11310496	Both
cg16778809	Both
cg15239579	Both
cg23372001	Both
cg01265637	Both
cg25776555	Both
cg13316424	Both
cg24309555	Both
cg00176210	Both
cg01377911	Both
cg06813842	Both
cg16443939	Both
cg24427660	Both
cg07749808	Both
cg00318573	Both

cg01405107	Both
cg24794531	Both
cg26813908	Both
cg17669700	Both
cg03390211	Both
cg13471990	Both
cg07115820	Both
cg19985911	Both
cg17204100	Both
cg24891133	Both
cg06121469	Both
cg01531431	Both
cg04825431	Both
cg19662708	Both
cg07737781	Both
cg13299148	Both
cg23174481	Both
cg26524899	Both
cg09437522	Both
cg24916177	Both
cg26259865	Both
cg12343777	Both
cg19000186	Both
cg08124399	Both
cg21003606	Both
cg15565533	Both
cg18190433	Both
cg08495878	Both
cg19876838	Both
cg27079104	Both
cg02580606	Both
cg05916196	Both
cg10910525	Both
cg18834029	Both
cg02693857	Both
cg15156078	Both
cg07460665	Both
cg18858343	Both
cg05388361	Both
cg24922045	Both
cg04790936	Both
cg07364841	Both
cg00040873	Both
cg16268051	Both
cg14019317	Both
cg25764464	Both
cg21481775	Both
cg17921310	Both
cg12229172	Both
cg19355190	Both
cg00769520	Both
cg05215575	Both
cg07399355	Both

cg09658438	Both
cg06189133	Both
cg18915143	Both
cg17813891	Both
cg26424956	Both
cg22030890	Both
cg09111917	Both
cg22139563	Both
cg04070847	Both
cg06162003	Both
cg00237010	Both
cg08265265	Both
cg02629761	Both
cg03996822	Both
cg20163033	Both
cg01283289	Both
cg19534945	Both
cg05469695	Both
cg26162695	Both
cg23911465	Both
cg20691580	Both
cg08185661	Both
cg24815853	Both
cg06957329	Both
cg19511844	Both
cg08450168	Both
cg18219951	Both
cg20140452	Both
cg16640096	Both
cg14217157	Both
cg06818777	Both
cg12459502	Both
cg20945531	Both
cg20430816	Both
cg05510041	Both
cg13144004	Both
cg26333641	Both
cg15726245	Both
cg14174099	Both
cg12072001	Both
cg24546942	Both
cg15415507	Both
cg01965939	Both
cg26026726	Both
cg09530763	Both
cg08590939	Both
cg02233559	Both
cg03884783	Both
cg03608577	Both
cg17892556	Both
cg06150803	Both
cg05134796	Both
cg09793866	Both

cg21194776	Both
cg15835232	Both
cg17800442	Both
cg26962295	Both
cg19666391	Both
cg01838317	Both
cg22233974	Both
cg17145652	Both
cg20017995	Both
cg11513856	Both
cg04732193	Both
cg14723032	Both
cg00043004	Both
cg03731898	Both
cg23370883	Both
cg11957040	Both
cg08008403	Both
cg20420433	Both
cg22502502	Both
cg19781053	Both
cg16725130	Both
cg12302621	Both
cg01657380	Both
cg19289461	Both
cg09630404	Both
cg12201164	Both
cg11398680	Both
cg07512517	Both
cg01565918	Both
cg27247832	Both
cg10951619	Both
cg22855405	Both
cg03498559	Both
cg24929737	Both
cg19954000	Both
cg14143055	Both
cg00728602	Both
cg01354473	Both
cg22024657	Both
cg17914753	Both
cg24607535	Both
cg15214137	Both
cg13351583	Both
cg19135706	Both
cg26535072	Both
cg17233935	Both
cg00332153	Both
cg07889201	Both
cg07059052	Both
cg14409941	Both
cg23033024	Both
cg27016307	Both
cg18368125	Both

cg22234962	Both
cg19853494	Both
cg17676129	Both
cg25219333	Both
cg15473904	Both
cg24736099	Both
cg27524460	Both
cg05412531	Both
cg10215884	Both
cg22197787	Both
cg12118465	Both
cg15513137	Both
cg03019000	Both
cg26904406	Both
cg14550007	Both
cg17582777	Both
cg10198932	Both
cg14789259	Both
cg12379775	Both
cg02961200	Both
cg11308639	Both
cg23191950	Both
cg18705301	Both
cg03380773	Both
cg26970800	Both
cg02250787	Both
cg24235037	Both
cg24624841	Both
cg03552103	Both
cg05126264	Both
cg08717396	Both
cg10003443	Both
cg01532771	Both
cg09864990	Both
cg05882691	Both
cg04273431	Both
cg14223017	Both
cg03386869	Both
cg19257200	Both
cg18821742	Both
cg06921282	Both
cg24087944	Both
cg02237119	Both
cg10014293	Both
cg05206661	Both
cg21137417	Both
cg12044210	Both
cg06885524	Both
cg21365602	Both
cg01722450	Both
cg15560337	Both
cg10479672	Both
cg14258236	Both

cg16878652	Both
cg04121771	Both
cg20320468	Both
cg26200580	Both
cg00446235	Both
cg23873703	Both
cg03649060	Both
cg13059335	Both
cg11846236	Both
cg04454391	Both
cg05411032	Both
cg05322222	Both
cg20772904	Both
cg02151301	Both
cg03503295	Both
cg14159818	Both
cg13033054	Both
cg20142224	Both
cg03179866	Both
cg24813212	Both
cg03364504	Both
cg03604278	Both
cg20526800	Both
cg03578041	Both
cg22708914	Both
cg24229963	Both
cg11591325	Both
cg02433671	Both
cg23851011	Both
cg15703357	Both
cg16377872	Both
cg13854874	Both
cg18177682	Both
cg09906458	Both
cg05446471	Both
cg02504465	Both
cg01311051	Both
cg20451680	Both
cg17741572	Both
cg11326613	Both
cg17754680	Both
cg13269407	Both
cg08268099	Both
cg02593766	Both
cg22681784	Both
cg20385229	Both
cg18088775	Both
cg21038703	Both
cg08008233	Both
cg23737768	Both
cg13384396	Both
cg00420929	Both
cg21269897	Both

cg12286890	Both
cg03192551	Both
cg09941381	Both
cg16270990	Both
cg11130441	Both
cg02228185	Both
cg19256236	Both
cg20286074	Both
cg10269439	Both
cg04678793	Both
cg12681402	Both
cg09419116	Both
cg08270477	Both
cg17124700	Both
cg20098659	Both
cg25839766	Both
cg25809905	Both
cg06351503	Both
cg02552572	Both
cg07634706	Both
cg26824408	Both
cg07404485	Both
cg12865837	Both
cg17606194	Both
cg13821008	Both
cg16196602	Both
cg14712964	Both
cg16382322	Both
cg23886551	Both
cg04833845	Both
cg25527547	Both
cg21942438	Both
cg20495040	Both
cg12293634	Both
cg22013966	Both
cg00033773	Both
cg07123069	Both
cg24890043	Both
cg10134939	Both
cg11201532	Both
cg05615487	Both
cg11795262	Both
cg04856043	Both
cg16175725	Both
cg14971781	Both
cg18270343	Both
cg09022808	Both
cg03109066	Both
cg03811411	Both
cg14470209	Both
cg22521310	Both
cg09022993	Both
cg15820955	Both

cg00707317	Both
cg20484002	Both
cg18060199	Both
cg21712678	Both
cg03000846	Both
cg16639185	Both
cg22638542	Both
cg07778029	Both
cg06112415	Both
cg26124016	Both
cg27223827	Both
cg09697795	Both
cg17753124	Both
cg20359349	Both
cg11799561	Both
cg04945541	Both
cg25499099	Both
cg20161089	Both
cg23617760	Both
cg14446712	Both
cg17272094	Both
cg00674922	Both
cg05060662	Both
cg09676390	Both
cg02525756	Both
cg00779924	Both
cg11454415	Both
cg08674093	Both
cg25026013	Both
cg08521225	Both
cg16174071	Both
cg09500672	Both
cg25454890	Both
cg17412351	Both
cg21182322	Both
cg12454725	Both
cg05222924	Both
cg20647888	Both
cg01966105	Both
cg17398613	Both
cg10277195	Both
cg11675413	Both
cg27011042	Both
cg12095491	Both
cg05626013	Both
cg00242839	Both
cg21949781	Both
cg05885123	Both
cg02171545	Both
cg25267732	Both
cg08331840	Both
cg06131936	Both
cg23591869	Both

cg06025017	Both
cg21926612	Both
cg03973663	Both
cg02966851	Both
cg09300114	Both
cg02364642	Both
cg08527127	Both
cg06161930	Both
cg22131172	Both
cg17778120	Both
cg22377428	Both
cg18392482	Both
cg13296371	Both
cg22341104	Both
cg16131766	Both
cg17704839	Both
cg10976673	Both
cg02938601	Both
cg27567335	Both
cg04049033	Both
cg09277575	Both
cg11786750	Both
cg19717150	Both
cg22419732	Both
cg27100123	Both
cg24430616	Both
cg23867494	Both
cg15659828	Both
cg09947274	Both
cg00888007	Both
cg13504059	Both
cg06276653	Both
cg07265310	Both
cg19203504	Both
cg21010050	Both
cg26646411	Both
cg15307268	Both
cg16464322	Both
cg20716119	Both
cg11476254	Both
cg01732037	Both
cg06822995	Both
cg07747336	Both
cg12869240	Both
cg02626977	Both
cg20496643	Both
cg03112782	Both
cg22166290	Both
cg26879282	Both
cg08513100	Both
cg06529761	Both
cg19123107	Both
cg02717046	Both

cg15501381	Both
cg06245154	Both
cg17812431	Both
cg24733384	Both
cg14163776	Both
cg08788717	Both
cg18105315	Both
cg22367264	Both
cg25225073	Both
cg12266024	Both
cg19099213	Both
cg22349489	Both
cg13164309	Both
cg16468910	Both
cg09082287	Both
cg12428416	Both
cg02517489	Both
cg25824330	Both
cg20358834	Both
cg16465939	Both
cg03163246	Both
cg05024638	Both
cg21299958	Both
cg06508934	Both
cg26986815	Both
cg00845900	Both
cg25843439	Both
cg21208104	Both
cg21489873	Both
cg03916421	Both
cg02451670	Both
cg19982860	Both
cg26304237	Both
cg11808874	Both
cg04167054	Both
cg19995014	Both
cg07043494	Both
cg24986868	Both
cg08487038	Both
cg14225485	Both
cg10987882	Both
cg20377762	Both
cg15572745	Both
cg22580372	Both
cg02505409	Both
cg25181693	Both
cg24211388	Both
cg23382741	Both
cg05780616	Both
cg08427977	Both
cg15227982	Both
cg00186701	Both
cg27127056	Both

cg06506864	Both
cg09034896	Both
cg09179743	Both
cg01703884	Both
cg17503456	Both
cg26018901	Both
cg09191232	Both
cg16802646	Both
cg08374799	Both
cg21522797	Both
cg24417499	Both
cg10507231	Both
cg12532500	Both
cg11225410	Both
cg12552392	Both
cg00451635	Both
cg04448487	Both
cg27613076	Both
cg10637955	Both
cg27210136	Both
cg10818781	Both
cg19292712	Both
cg13230584	Both
cg01359534	Both
cg03533811	Both
cg06302803	Both
cg17366294	Both
cg25104030	Both
cg22021786	Both
cg13018903	Both
cg19840532	Both
cg12792011	Both
cg26656452	Both
cg02494117	Both
cg05674444	Both
cg09224952	Both
cg02643667	Both
cg22115808	Both
cg27291231	Both
cg17733331	Both
cg25573386	Both
cg22156632	Both
cg14633704	Both
cg02706575	Both
cg18907029	Both
cg20870362	Both
cg00098162	Both
cg07758574	Both
cg19141563	Both
cg24901474	Both
cg26673195	Both
cg15503752	Both
cg14871138	Both

cg10408410	Both
cg15649193	Both
cg05008406	Both
cg10055471	Both
cg10375110	Both
cg04905829	Both
cg08304608	Both
cg15209169	Both
cg04629204	Both
cg14533138	Both
cg18986964	Both
cg26033710	Both
cg11399100	Both
cg24068372	Both
cg12647587	Both
cg13618372	Both
cg23180489	Both
cg23548479	Both
cg19138960	Both
cg20380069	Both
cg08045757	Both
cg07510052	Both
cg05440289	Both
cg05942574	Both
cg15169162	Both
cg27034836	Both
cg18741908	Both
cg00563845	Both
cg06575572	Both
cg27563778	Both
cg05022306	Both
cg05564657	Both
cg15590780	Both
cg26796190	Both
cg21006686	Both
cg17541922	Both
cg25717844	Both
cg03842617	Both
cg15435730	Both
cg24937706	Both
cg08261841	Both
cg17342759	Both
cg05547500	Both
cg08395899	Both
cg05590294	Both
cg12437481	Both
cg02855426	Both
cg16425577	Both
cg14223995	Both
cg17910564	Both
cg17165266	Both
cg20919799	Both
cg05064352	Both

cg09015232	Both
cg09239756	Both
cg07207789	Both
cg23626798	Both
cg24042452	Both
cg16585682	Both
cg14267151	Both
cg24690731	Both
cg19914607	Both
cg13830624	Both
cg01605783	Both
cg22751696	Both
cg26145103	Both
cg22190114	Both
cg04603184	Both
cg23602533	Both
cg24467291	Both
cg20663831	Both
cg22991148	Both
cg16861508	Both
cg22957381	Both
cg16473288	Both
cg10006582	Both
cg09547190	Both
cg19706682	Both
cg23137807	Both
cg05038209	Both
cg14588638	Both
cg21043754	Both
cg04080057	Both
cg11458974	Both
cg01169778	Both
cg03780733	Both
cg11540997	Both
cg24335895	Both
cg18180783	Both
cg21982518	Both
cg18885299	Both
cg18555555	Both
cg01263716	Both
cg22213042	Both
cg00592315	Both
cg21717724	Both
cg06564900	Both
cg11956146	Both
cg13765621	Both
cg16778148	Both
cg24677780	Both
cg23645091	Both
cg27600136	Both
cg10266490	Both
cg25924032	Both
cg03552688	Both

cg21259253	Both
cg23455614	Both
cg03143333	Both
cg19403346	Both
cg04868764	Both
cg26803305	Both
cg22490255	Both
cg05801648	Both
cg13015534	Both
cg15975283	Both
cg08878323	Both
cg01457622	Both
cg25955969	Both
cg06716436	Both
cg16673198	Both
cg19409156	Both
cg19278780	Both
cg13237829	Both
cg07640473	Both
cg07589355	Both
cg04719491	Both
cg22620680	Both
cg25928579	Both
cg09429111	Both
cg21415604	Both
cg08762247	Both
cg16332577	Both
cg19184963	Both
cg11126313	Both
cg19118533	Both
cg14030359	Both
cg23651356	Both
cg14580737	Both
cg27210390	Both
cg17192247	Both
cg26459500	Both
cg18641937	Both
cg11819637	Both
cg21979032	Both
cg26489108	Both
cg21574752	Both
cg26511075	Both
cg02828104	Both
cg25412453	Both
cg07256847	Both
cg19008649	Both
cg20994561	Both
cg05755779	Both
cg27543230	Both
cg09998229	Both
cg11879514	Both
cg25834275	Both
cg24506604	Both

cg17088290	Both
cg06210526	Both
cg21134096	Both
cg06452647	Both
cg20797699	Both
cg24549507	Both
cg07880854	Both
cg17795586	Both
cg14904464	Both
cg01428678	Both
cg13086467	Both
cg20277416	Both
cg06288632	Both
cg26299767	Both
cg05942970	Both
cg24727203	Both
cg11027330	Both
cg15639045	Both
cg25438415	Both
cg03161839	Both
cg23623692	Both
cg25425005	Both
cg09298623	Both
cg14741922	Both
cg16399745	Both
cg07774025	Both
cg23621817	Both
cg07008386	Both
cg04121983	Both
cg04330084	Both
cg05293216	Both
cg15309066	Both
cg21281799	Both
cg07711515	Both
cg09541248	Both
cg03063797	Both
cg08434234	Both
cg12514506	Both
cg15239123	Both
cg21416022	Both
cg03732545	Both
cg24754277	Both
cg14563260	Both
cg01200177	Both
cg25229172	Both
cg26090660	Both
cg08548888	Both
cg10660136	Both
cg00659129	Both
cg04553559	Both
cg17963840	Both
cg08335125	Both
cg08888956	Both

cg10498097	Both
cg17229197	Both
cg13968061	Both
cg27214774	Both
cg26672426	Both
cg08367838	Both
cg21183846	Both
cg27662379	Both
cg19769182	Both
cg04765422	Both
cg19930802	Both
cg18059933	Both
cg09037858	Both
cg26090652	Both
cg26333317	Both
cg19560287	Both
cg10358212	Both
cg16641915	Both
cg03700308	Both
cg16794682	Both
cg25620220	Both
cg07371290	Both
cg13438834	Both
cg18335243	Both
cg18433086	Both
cg18380975	Both
cg26533949	Both
cg11856093	Both
cg20732137	Both
cg19579782	Both
cg16051685	Both
cg03097995	Both
cg09082921	Both
cg10421561	Both

Term	Description	General RCC, More or Less Methylated in Tumors	ccRCC, More or Less Methylated in Tumors
GO:0004871	signal transducer activity	Both	Both
GO:0004872	receptor activity	Both	Both
GO:0004888	transmembrane signaling receptor activity	Both	Both
GO:0004930	G-protein coupled receptor activity	Both	Both
GO:0005576	extracellular region	Both	Both
GO:0005615	extracellular space	Both	Both
GO:0005886	plasma membrane	Both	Both
GO:0005887	integral to plasma membrane	Both	Both
GO:0016021	integral to membrane	Both	Both
GO:0031224	intrinsic to membrane	Both	Both
GO:0031226	intrinsic to plasma membrane	Both	Both
GO:0038023	signaling receptor activity	Both	Both
GO:0044421	extracellular region part	Both	Both
GO:0044425	membrane part	Both	Both
GO:0044459	plasma membrane part	Both	Both
GO:0060089	molecular transducer activity	Both	Both
KEGG_MAPK_SIGNALING_PATHWAY	MAPK signaling pathway	Both	Both
KEGG_NEUROACTIVE_LIGAND_RECEPT	Neuroactive ligand-receptor interaction	Both	Both
KEGG_PATHWAYS_IN_CANCER	Pathways in cancer	Both	Both
REACTOME_CLASS_A1_RHODOPSIN_LI	Genes involved in Class A/1 (Rhodopsin-lik	Both	Both
REACTOME_G_ALPHA_I_SIGNALLING_E	Genes involved in G alpha (i) signalling eve	Both	Both
REACTOME_GASTRIN_CREB_SIGNALLI	Genes involved in Gastrin-CREB signalling	Both	Both
REACTOME_GPCR_DOWNSTREAM_SIG	Genes involved in GPCR downstream signa	Both	Both
REACTOME_GPCR_LIGAND_BINDING	Genes involved in GPCR ligand binding	Both	Both
REACTOME_HEMOSTASIS	Genes involved in Hemostasis	Both	Both
REACTOME_NEURONAL_SYSTEM	Genes involved in Neuronal System	Both	Both
REACTOME_PEPTIDE_LIGAND_BINDING	Genes involved in Peptide ligand-binding re	Both	Both
REACTOME_SIGNALING_BY_GPCR	Genes involved in Signaling by GPCR	Both	Both
REACTOME_SLC_MEDIATED_TRANSME	Genes involved in SLC-mediated transmem	Both	Both
REACTOME_TRANSMEMBRANE_TRANS	Genes involved in Transmembrane transpo	Both	Both
GO:0001653	peptide receptor activity	More	Both
GO:0005102	receptor binding	More	Both
GO:0007186	G-protein coupled receptor signaling pathw	More	Both
GO:0016020	membrane	More	Both
REACTOME_G_ALPHA_Q_SIGNALLING_	Genes involved in G alpha (q) signalling ev	More	Both
REACTOME_TRANSMISSION_ACROSS_	Genes involved in Transmission across Che	More	Both
GO:0001816	cytokine production	NA	Less
GO:0001878	response to yeast	NA	Less
GO:0002020	protease binding	NA	Less
GO:0002275	myeloid cell activation involved in immune r	NA	Less
GO:0002407	dendritic cell chemotaxis	NA	Less
GO:0002685	regulation of leukocyte migration	NA	Less
GO:0002700	regulation of production of molecular media	NA	Less
GO:0002712	regulation of B cell mediated immunity	NA	Less
GO:0004252	serine-type endopeptidase activity	NA	Less
GO:0004497	monooxygenase activity	NA	Less
GO:0004522	pancreatic ribonuclease activity	NA	Less
GO:0004620	phospholipase activity	NA	Less
GO:0004629	phospholipase C activity	NA	Less
GO:0004857	enzyme inhibitor activity	NA	Less
GO:0005164	tumor necrosis factor receptor binding	NA	Less
GO:0006805	xenobiotic metabolic process	NA	Less
GO:0007159	leukocyte cell-cell adhesion	NA	Less
GO:0008236	serine-type peptidase activity	NA	Less
GO:0008289	lipid binding	NA	Less
GO:0009055	electron carrier activity	NA	Less
GO:0009264	deoxyribonucleotide catabolic process	NA	Less
GO:0009593	detection of chemical stimulus	NA	Less
GO:0009605	response to external stimulus	NA	Less
GO:0010466	negative regulation of peptidase activity	NA	Less
GO:0010528	regulation of transposition	NA	Less
GO:0010529	negative regulation of transposition	NA	Less
GO:0010896	regulation of triglyceride catabolic process	NA	Less
GO:0016298	lipase activity	NA	Less
GO:0016810	hydrolase activity, acting on carbon-nitroger	NA	Less
GO:0017171	serine hydrolase activity	NA	Less
GO:0020037	heme binding	NA	Less
GO:0030246	carbohydrate binding	NA	Less
GO:0032101	regulation of response to external stimulus	NA	Less
GO:0032103	positive regulation of response to external s	NA	Less
GO:0032649	regulation of interferon-gamma production	NA	Less
GO:0032680	regulation of tumor necrosis factor productio	NA	Less
GO:0032760	positive regulation of tumor necrosis factor	NA	Less
GO:0032813	tumor necrosis factor receptor superfamily b	NA	Less
GO:0032946	positive regulation of mononuclear cell proli	NA	Less
GO:0033003	regulation of mast cell activation	NA	Less
GO:0034097	response to cytokine stimulus	NA	Less
GO:0034367	macromolecular complex remodeling	NA	Less
GO:0034368	protein-lipid complex remodeling	NA	Less
GO:0034369	plasma lipoprotein particle remodeling	NA	Less
GO:0036150	phosphatidylserine acyl-chain remodeling	NA	Less
GO:0036336	dendritic cell migration	NA	Less
GO:0042102	positive regulation of T cell proliferation	NA	Less
GO:0042345	regulation of NF-kappaB import into nucleus	NA	Less
GO:0042379	chemokine receptor binding	NA	Less
GO:0042592	homeostatic process	NA	Less
GO:0046135	pyrimidine nucleoside catabolic process	NA	Less
GO:0046889	positive regulation of lipid biosynthetic proc	NA	Less
GO:0046906	tetrapyrrole binding	NA	Less
GO:0047760	butyrate-CoA ligase activity	NA	Less
GO:0048584	positive regulation of response to stimulus	NA	Less
GO:0050671	positive regulation of lymphocyte proliferatio	NA	Less
GO:0050709	negative regulation of protein secretion	NA	Less
GO:0050727	regulation of inflammatory response	NA	Less
GO:0050729	positive regulation of inflammatory respons	NA	Less
GO:0050853	B cell receptor signaling pathway	NA	Less
GO:0050864	regulation of B cell activation	NA	Less
GO:0050906	detection of stimulus involved in sensory pe	NA	Less
GO:0050907	detection of chemical stimulus involved in s	NA	Less
GO:0050911	detection of chemical stimulus involved in s	NA	Less
GO:0051050	positive regulation of transport	NA	Less
GO:0051222	positive regulation of protein transport	NA	Less

GO:0051606	detection of stimulus	NA	Less
GO:0051852	disruption by host of symbiont cells	NA	Less
GO:0051873	killing by host of symbiont cells	NA	Less
GO:0052547	regulation of peptidase activity	NA	Less
GO:0052548	regulation of endopeptidase activity	NA	Less
GO:0065008	regulation of biological quality	NA	Less
GO:0070330	aromatase activity	NA	Less
GO:0070665	positive regulation of leukocyte proliferation	NA	Less
GO:0072529	pyrimidine-containing compound catabolic p	NA	Less
GO:0090207	regulation of triglyceride metabolic process	NA	Less
GO:2000106	regulation of leukocyte apoptotic process	NA	Less
REACTOME_PHASE1_FUNCTIONALIZAT	Genes involved in Phase 1 - Functionalizati	NA	Less
KEGG_ARACHIDONIC_ACID_METABOLIS	Arachidonic acid metabolism	NA	Less
REACTOME_PLATELET_ACTIVATION_SI	Genes involved in Platelet activation, signal	NA	Less
KEGG_COMPLEMENT_AND_COAGULAT	Complement and coagulation cascades	NA	Less
KEGG_DRUG_METABOLISM_CYTOCHR(	Drug metabolism - cytochrome P450	NA	Less
KEGG_AUTOIMMUNE_THYROID_DISEAS	Autoimmune thyroid disease	NA	Less
PID_CD8TCRDOWNSTREAMPATHWAY	Downstream signaling in naïve CD8+	NA	Less
GO:0001533	cornified envelope	Less	Less
GO:0001637	G-protein coupled chemoattractant receptor	Less	Less
GO:0001775	cell activation	Less	Less
GO:0001817	regulation of cytokine production	Less	Less
GO:0001906	cell killing	Less	Less
GO:0002237	response to molecule of bacterial origin	Less	Less
GO:0002252	immune effector process	Less	Less
GO:0002253	activation of immune response	Less	Less
GO:0002263	cell activation involved in immune response	Less	Less
GO:0002274	myeloid leukocyte activation	Less	Less
GO:0002366	leukocyte activation involved in immune res	Less	Less
GO:0002376	immune system process	Less	Less
GO:0002429	immune response-activating cell surface re	Less	Less
GO:0002526	acute inflammatory response	Less	Less
GO:0002682	regulation of immune system process	Less	Less
GO:0002683	negative regulation of immune system proc	Less	Less
GO:0002684	positive regulation of immune system proce	Less	Less
GO:0002694	regulation of leukocyte activation	Less	Less
GO:0002695	negative regulation of leukocyte activation	Less	Less
GO:0002696	positive regulation of leukocyte activation	Less	Less
GO:0002697	regulation of immune effector process	Less	Less
GO:0002699	positive regulation of immune effector proce	Less	Less
GO:0002703	regulation of leukocyte mediated immunity	Less	Less
GO:0002704	negative regulation of leukocyte mediated i	Less	Less
GO:0002706	regulation of lymphocyte mediated immunity	Less	Less
GO:0002714	positive regulation of B cell mediated immu	Less	Less
GO:0002757	immune response-activating signal transdu	Less	Less
GO:0002819	regulation of adaptive immune response	Less	Less
GO:0002822	regulation of adaptive immune response ba	Less	Less
GO:0002861	regulation of inflammatory response to anti	Less	Less
GO:0002889	regulation of immunoglobulin mediated imm	Less	Less
GO:0002891	positive regulation of immunoglobulin media	Less	Less
GO:0003823	antigen binding	Less	Less
GO:0004866	endopeptidase inhibitor activity	Less	Less
GO:0004867	serine-type endopeptidase inhibitor activity	Less	Less
GO:0004950	chemokine receptor activity	Less	Less
GO:0004984	olfactory receptor activity	Less	Less
GO:0005125	cytokine activity	Less	Less
GO:0005126	cytokine receptor binding	Less	Less
GO:0005882	intermediate filament	Less	Less
GO:0006935	chemotaxis	Less	Less
GO:0006950	response to stress	Less	Less
GO:0006952	defense response	Less	Less
GO:0006953	acute-phase response	Less	Less
GO:0006954	inflammatory response	Less	Less
GO:0006955	immune response	Less	Less
GO:0006959	humoral immune response	Less	Less
GO:0006968	cellular defense response	Less	Less
GO:0008009	chemokine activity	Less	Less
GO:0008329	signaling pattern recognition receptor activi	Less	Less
GO:0009595	detection of biotic stimulus	Less	Less
GO:0009607	response to biotic stimulus	Less	Less
GO:0009611	response to wounding	Less	Less
GO:0009615	response to virus	Less	Less
GO:0009617	response to bacterium	Less	Less
GO:0009620	response to fungus	Less	Less
GO:0009897	external side of plasma membrane	Less	Less
GO:0016712	oxidoreductase activity, acting on paired do	Less	Less
GO:0019221	cytokine-mediated signaling pathway	Less	Less
GO:0019864	IgG binding	Less	Less
GO:0019865	immunoglobulin binding	Less	Less
GO:0030414	peptidase inhibitor activity	Less	Less
GO:0030593	neutrophil chemotaxis	Less	Less
GO:0030595	leukocyte chemotaxis	Less	Less
GO:0031294	lymphocyte costimulation	Less	Less
GO:0031295	T cell costimulation	Less	Less
GO:0031347	regulation of defense response	Less	Less
GO:0031349	positive regulation of defense response	Less	Less
GO:0031424	keratinization	Less	Less
GO:0031640	killing of cells of other organism	Less	Less
GO:0032496	response to lipopolysaccharide	Less	Less
GO:0032732	positive regulation of interleukin-1 productio	Less	Less
GO:0032944	regulation of mononuclear cell proliferation	Less	Less
GO:0034341	response to interferon-gamma	Less	Less
GO:0038187	pattern recognition receptor activity	Less	Less
GO:0042110	T cell activation	Less	Less
GO:0042116	macrophage activation	Less	Less
GO:0042129	regulation of T cell proliferation	Less	Less
GO:0042330	taxis	Less	Less
GO:0042737	drug catabolic process	Less	Less
GO:0042742	defense response to bacterium	Less	Less
GO:0043901	negative regulation of multi-organism proce	Less	Less
GO:0044126	regulation of growth of symbiont in host	Less	Less
GO:0044130	negative regulation of growth of symbiont in	Less	Less
GO:0044144	modulation of growth of symbiont involved i	Less	Less

GO:0044146	negative regulation of growth of symbiont in	Less	Less
GO:0044364	disruption of cells of other organism	Less	Less
GO:0045071	negative regulation of viral genome replicat	Less	Less
GO:0045087	innate immune response	Less	Less
GO:0045095	keratin filament	Less	Less
GO:0045321	leukocyte activation	Less	Less
GO:0046133	pyrimidine ribonucleoside catabolic process	Less	Less
GO:0046649	lymphocyte activation	Less	Less
GO:0050670	regulation of lymphocyte proliferation	Less	Less
GO:0050704	regulation of interleukin-1 secretion	Less	Less
GO:0050707	regulation of cytokine secretion	Less	Less
GO:0050708	regulation of protein secretion	Less	Less
GO:0050714	positive regulation of protein secretion	Less	Less
GO:0050715	positive regulation of cytokine secretion	Less	Less
GO:0050716	positive regulation of interleukin-1 secretion	Less	Less
GO:0050764	regulation of phagocytosis	Less	Less
GO:0050766	positive regulation of phagocytosis	Less	Less
GO:0050776	regulation of immune response	Less	Less
GO:0050778	positive regulation of immune response	Less	Less
GO:0050829	defense response to Gram-negative bacteri	Less	Less
GO:0050830	defense response to Gram-positive bacteri	Less	Less
GO:0050832	defense response to fungus	Less	Less
GO:0050851	antigen receptor-mediated signaling pathwa	Less	Less
GO:0050863	regulation of T cell activation	Less	Less
GO:0050865	regulation of cell activation	Less	Less
GO:0050866	negative regulation of cell activation	Less	Less
GO:0050867	positive regulation of cell activation	Less	Less
GO:0050868	negative regulation of T cell activation	Less	Less
GO:0050870	positive regulation of T cell activation	Less	Less
GO:0050896	response to stimulus	Less	Less
GO:0050900	leukocyte migration	Less	Less
GO:0051249	regulation of lymphocyte activation	Less	Less
GO:0051250	negative regulation of lymphocyte activation	Less	Less
GO:0051251	positive regulation of lymphocyte activation	Less	Less
GO:0051607	defense response to virus	Less	Less
GO:0051704	multi-organism process	Less	Less
GO:0051707	response to other organism	Less	Less
GO:0060326	cell chemotaxis	Less	Less
GO:0061134	peptidase regulator activity	Less	Less
GO:0061135	endopeptidase regulator activity	Less	Less
GO:0070098	chemokine-mediated signaling pathway	Less	Less
GO:0070663	regulation of leukocyte proliferation	Less	Less
GO:0071216	cellular response to biotic stimulus	Less	Less
GO:0071219	cellular response to molecule of bacterial or	Less	Less
GO:0071222	cellular response to lipopolysaccharide	Less	Less
GO:0071346	cellular response to interferon-gamma	Less	Less
GO:0071621	granulocyte chemotaxis	Less	Less
GO:1902105	regulation of leukocyte differentiation	Less	Less
GO:2000107	negative regulation of leukocyte apoptotic p	Less	Less
KEGG_APOPTOSIS	Apoptosis	Less	Less
KEGG_CELL_ADHESION_MOLECULES_(	Cell adhesion molecules (CAMs)	Less	Less
KEGG_CHEMOKINE_SIGNALING_PATHW	Chemokine signaling pathway	Less	Less
KEGG_CYTOKINE_CYTOKINE_RECEPTO	Cytokine-cytokine receptor interaction	Less	Less
KEGG_GRAFT_VERSUS_HOST_DISEAS	Graft-versus-host disease	Less	Less
KEGG_HEMATOPOIETIC_CELL_LINEAGE	Hematopoietic cell lineage	Less	Less
KEGG_JAK_STAT_SIGNALING_PATHWAY	Jak-STAT signaling pathway	Less	Less
KEGG_LEISHMANIA_INFECTION	Leishmania infection	Less	Less
KEGG_LEUKOCYTE_TRANSENDOTHELI	Leukocyte transendothelial migration	Less	Less
KEGG_NATURAL_KILLER_CELL_MEDIAT	Natural killer cell mediated cytotoxicity	Less	Less
KEGG_T_CELL_RECEPTOR_SIGNALING	T cell receptor signaling pathway	Less	Less
KEGG_TOLL_LIKE_RECEPTOR_SIGNALI	Toll-like receptor signaling pathway	Less	Less
PID_CD8TCRPATHWAY	TCR signaling in naïve CD8+ T cells	Less	Less
PID_CXCR4_PATHWAY	CXCR4-mediated signaling events	Less	Less
PID_TCR_PATHWAY	TCR signaling in naïve CD4+ T cells	Less	Less
REACTOME_ADAPTIVE_IMMUNE_SYSTE	Genes involved in Adaptive Immune System	Less	Less
REACTOME_BIOLOGICAL_OXIDATIONS	Genes involved in Biological oxidations	Less	Less
REACTOME_CELL_SURFACE_INTERACT	Genes involved in Cell surface interactions	Less	Less
REACTOME_CHEMOKINE_RECEPTORS	Genes involved in Chemokine receptors bin	Less	Less
REACTOME_CYTOCHROME_P450_ARRA	Genes involved in Cytochrome P450 - arran	Less	Less
REACTOME_CYTOKINE_SIGNALING_IN_	Genes involved in Cytokine Signaling in Imm	Less	Less
REACTOME_IMMUNE_SYSTEM	Genes involved in Immune System	Less	Less
REACTOME_IMMUNOREGULATORY_INT	Genes involved in Immunoregulatory intera	Less	Less
REACTOME_INNATE_IMMUNE_SYSTEM	Genes involved in Innate Immune System	Less	Less
REACTOME_INTERFERON_ALPHA_BETA	Genes involved in Interferon alpha/beta sig	Less	Less
REACTOME_INTERFERON_SIGNALING	Genes involved in Interferon Signaling	Less	Less
REACTOME_METABOLISM_OF_LIPIDS_A	Genes involved in Metabolism of lipids and	Less	Less
GO:0000977	RNA polymerase II regulatory region seque	NA	More
GO:0001525	angiogenesis	NA	More
GO:0001649	osteoblast differentiation	NA	More
GO:0001942	hair follicle development	NA	More
GO:0003705	RNA polymerase II distal enhancer sequenc	NA	More
GO:0005581	collagen	NA	More
GO:0006022	aminoglycan metabolic process	NA	More
GO:0006814	sodium ion transport	NA	More
GO:0007588	excretion	NA	More
GO:0008146	sulfotransferase activity	NA	More
GO:0008284	positive regulation of cell proliferation	NA	More
GO:0008328	ionotropic glutamate receptor complex	NA	More
GO:0009966	regulation of signal transduction	NA	More
GO:0009968	negative regulation of signal transduction	NA	More
GO:0010469	regulation of receptor activity	NA	More
GO:0015103	inorganic anion transmembrane transporter	NA	More
GO:0016055	Wnt receptor signaling pathway	NA	More
GO:0016323	basolateral plasma membrane	NA	More
GO:0016782	transferase activity, transferring sulfur-conta	NA	More
GO:0030002	cellular anion homeostasis	NA	More
GO:0030018	Z disc	NA	More
GO:0030178	negative regulation of Wnt receptor signalin	NA	More
GO:0030203	glycosaminoglycan metabolic process	NA	More
GO:0030540	female genitalia development	NA	More
GO:0030574	collagen catabolic process	NA	More
GO:0030823	regulation of cGMP metabolic process	NA	More
GO:0030901	midbrain development	NA	More

GO:0031233	intrinsic to external side of plasma membra	NA	More
GO:0032281	alpha-amino-3-hydroxy-5-methyl-4-isoxazo	NA	More
GO:0034035	purine ribonucleoside bisphosphate metabo	NA	More
GO:0035137	hindlimb morphogenesis	NA	More
GO:0043235	receptor complex	NA	More
GO:0044243	multicellular organismal catabolic process	NA	More
GO:0044449	contractile fiber part	NA	More
GO:0046658	anchored to plasma membrane	NA	More
GO:0048168	regulation of neuronal synaptic plasticity	NA	More
GO:0048585	negative regulation of response to stimulus	NA	More
GO:0048771	tissue remodeling	NA	More
GO:0048863	stem cell differentiation	NA	More
GO:0050427	3'-phosphoadenosine 5'-phosphosulfate me	NA	More
GO:0050680	negative regulation of epithelial cell prolifer	NA	More
GO:0050808	synapse organization	NA	More
GO:0060537	muscle tissue development	NA	More
GO:0070293	renal absorption	NA	More
GO:0070848	response to growth factor stimulus	NA	More
GO:0071363	cellular response to growth factor stimulus	NA	More
GO:0072089	stem cell proliferation	NA	More
GO:0072507	divalent inorganic cation homeostasis	NA	More
REACTOME_NCAM1_INTERACTIONS	Genes involved in NCAM1 interactions	NA	More
KEGG_MELANOGENESIS	Melanogenesis	NA	More
REACTOME_CELL_CELL_JUNCTION_OF	Genes involved in Cell-cell junction organiz	NA	More
REACTOME_GENERIC_TRANSCRIPTION	Genes involved in Generic Transcription Pa	NA	More
GO:0000904	cell morphogenesis involved in differentiatio	More	More
GO:0000975	regulatory region DNA binding	More	More
GO:0000976	transcription regulatory region sequence-sp	More	More
GO:0000981	sequence-specific DNA binding RNA polym	More	More
GO:0000982	RNA polymerase II core promoter proximal	More	More
GO:0001067	regulatory region nucleic acid binding	More	More
GO:0001071	nucleic acid binding transcription factor acti	More	More
GO:0001077	RNA polymerase II core promoter proximal	More	More
GO:0001228	RNA polymerase II transcription regulatory	More	More
GO:0001501	skeletal system development	More	More
GO:0001508	regulation of action potential	More	More
GO:0001658	branching involved in ureteric bud morphog	More	More
GO:0001664	G-protein coupled receptor binding	More	More
GO:0001667	ameboidal cell migration	More	More
GO:0001708	cell fate specification	More	More
GO:0001755	neural crest cell migration	More	More
GO:0001763	morphogenesis of a branching structure	More	More
GO:0001764	neuron migration	More	More
GO:0001822	kidney development	More	More
GO:0001947	heart looping	More	More
GO:0002009	morphogenesis of an epithelium	More	More
GO:0002791	regulation of peptide secretion	More	More
GO:0003002	regionalization	More	More
GO:0003006	developmental process involved in reprodu	More	More
GO:0003008	system process	More	More
GO:0003012	muscle system process	More	More
GO:0003013	circulatory system process	More	More
GO:0003014	renal system process	More	More
GO:0003018	vascular process in circulatory system	More	More
GO:0003143	embryonic heart tube morphogenesis	More	More
GO:0003156	regulation of organ formation	More	More
GO:0003700	sequence-specific DNA binding transcriptio	More	More
GO:0004714	transmembrane receptor protein tyrosine ki	More	More
GO:0004716	receptor signaling protein tyrosine kinase a	More	More
GO:0005201	extracellular matrix structural constituent	More	More
GO:0005215	transporter activity	More	More
GO:0005216	ion channel activity	More	More
GO:0005230	extracellular ligand-gated ion channel activi	More	More
GO:0005231	excitatory extracellular ligand-gated ion cha	More	More
GO:0005244	voltage-gated ion channel activity	More	More
GO:0005245	voltage-gated calcium channel activity	More	More
GO:0005249	voltage-gated potassium channel activity	More	More
GO:0005251	delayed rectifier potassium channel activity	More	More
GO:0005261	cation channel activity	More	More
GO:0005262	calcium channel activity	More	More
GO:0005267	potassium channel activity	More	More
GO:0005326	neurotransmitter transporter activity	More	More
GO:0005328	neurotransmitter:sodium symporter activity	More	More
GO:0005509	calcium ion binding	More	More
GO:0005539	glycosaminoglycan binding	More	More
GO:0005578	proteinaceous extracellular matrix	More	More
GO:0006029	proteoglycan metabolic process	More	More
GO:0006140	regulation of nucleotide metabolic process	More	More
GO:0006811	ion transport	More	More
GO:0006812	cation transport	More	More
GO:0006813	potassium ion transport	More	More
GO:0006836	neurotransmitter transport	More	More
GO:0006928	cellular component movement	More	More
GO:0006936	muscle contraction	More	More
GO:0006937	regulation of muscle contraction	More	More
GO:0007154	cell communication	More	More
GO:0007155	cell adhesion	More	More
GO:0007156	homophilic cell adhesion	More	More
GO:0007165	signal transduction	More	More
GO:0007166	cell surface receptor signaling pathway	More	More
GO:0007187	G-protein coupled receptor signaling pathw	More	More
GO:0007188	adenylate cyclase-modulating G-protein co	More	More
GO:0007189	adenylate cyclase-activating G-protein coup	More	More
GO:0007200	phospholipase C-activating G-protein coupl	More	More
GO:0007204	elevation of cytosolic calcium ion concentra	More	More
GO:0007218	neuropeptide signaling pathway	More	More
GO:0007267	cell-cell signaling	More	More
GO:0007268	synaptic transmission	More	More
GO:0007270	neuron-neuron synaptic transmission	More	More
GO:0007389	pattern specification process	More	More
GO:0007399	nervous system development	More	More
GO:0007411	axon guidance	More	More
GO:0007422	peripheral nervous system development	More	More

GO:0007600	sensory perception	More	More
GO:0007610	behavior	More	More
GO:0007611	learning or memory	More	More
GO:0007612	learning	More	More
GO:0007613	memory	More	More
GO:0007616	long-term memory	More	More
GO:0007626	locomotory behavior	More	More
GO:0007631	feeding behavior	More	More
GO:0008076	voltage-gated potassium channel complex	More	More
GO:0008150	biological_process	More	More
GO:0008188	neuropeptide receptor activity	More	More
GO:0008201	heparin binding	More	More
GO:0008217	regulation of blood pressure	More	More
GO:0008227	G-protein coupled amine receptor activity	More	More
GO:0008324	cation transmembrane transporter activity	More	More
GO:0008344	adult locomotory behavior	More	More
GO:0008509	anion transmembrane transporter activity	More	More
GO:0008528	G-protein coupled peptide receptor activity	More	More
GO:0009187	cyclic nucleotide metabolic process	More	More
GO:0009653	anatomical structure morphogenesis	More	More
GO:0009719	response to endogenous stimulus	More	More
GO:0009887	organ morphogenesis	More	More
GO:0009888	tissue development	More	More
GO:0009952	anterior/posterior pattern specification	More	More
GO:0009953	dorsal/ventral pattern formation	More	More
GO:0010646	regulation of cell communication	More	More
GO:0010647	positive regulation of cell communication	More	More
GO:0010648	negative regulation of cell communication	More	More
GO:0010769	regulation of cell morphogenesis involved in	More	More
GO:0010810	regulation of cell-substrate adhesion	More	More
GO:0010817	regulation of hormone levels	More	More
GO:0010959	regulation of metal ion transport	More	More
GO:0010975	regulation of neuron projection developmen	More	More
GO:0014070	response to organic cyclic compound	More	More
GO:0015075	ion transmembrane transporter activity	More	More
GO:0015077	monovalent inorganic cation transmembran	More	More
GO:0015079	potassium ion transmembrane transporter a	More	More
GO:0015081	sodium ion transmembrane transporter acti	More	More
GO:0015085	calcium ion transmembrane transporter acti	More	More
GO:0015267	channel activity	More	More
GO:0015276	ligand-gated ion channel activity	More	More
GO:0015291	secondary active transmembrane transporte	More	More
GO:0015293	symporter activity	More	More
GO:0015370	solute:sodium symporter activity	More	More
GO:0015672	monovalent inorganic cation transport	More	More
GO:0016202	regulation of striated muscle tissue develop	More	More
GO:0016247	channel regulator activity	More	More
GO:0016324	apical plasma membrane	More	More
GO:0016337	cell-cell adhesion	More	More
GO:0016477	cell migration	More	More
GO:0019199	transmembrane receptor protein kinase act	More	More
GO:0019233	sensory perception of pain	More	More
GO:0021515	cell differentiation in spinal cord	More	More
GO:0021527	spinal cord association neuron differentiatio	More	More
GO:0021953	central nervous system neuron differentiatio	More	More
GO:0022404	molting cycle process	More	More
GO:0022405	hair cycle process	More	More
GO:0022414	reproductive process	More	More
GO:0022603	regulation of anatomical structure morphoge	More	More
GO:0022604	regulation of cell morphogenesis	More	More
GO:0022610	biological adhesion	More	More
GO:0022803	passive transmembrane transporter activity	More	More
GO:0022804	active transmembrane transporter activity	More	More
GO:0022832	voltage-gated channel activity	More	More
GO:0022834	ligand-gated channel activity	More	More
GO:0022836	gated channel activity	More	More
GO:0022838	substrate-specific channel activity	More	More
GO:0022839	ion gated channel activity	More	More
GO:0022843	voltage-gated cation channel activity	More	More
GO:0022857	transmembrane transporter activity	More	More
GO:0022890	inorganic cation transmembrane transporte	More	More
GO:0022891	substrate-specific transmembrane transport	More	More
GO:0022892	substrate-specific transporter activity	More	More
GO:0023051	regulation of signaling	More	More
GO:0023052	signaling	More	More
GO:0023056	positive regulation of signaling	More	More
GO:0023057	negative regulation of signaling	More	More
GO:0023061	signal release	More	More
GO:0030001	metal ion transport	More	More
GO:0030054	cell junction	More	More
GO:0030111	regulation of Wnt receptor signaling pathwa	More	More
GO:0030154	cell differentiation	More	More
GO:0030155	regulation of cell adhesion	More	More
GO:0030165	PDZ domain binding	More	More
GO:0030182	neuron differentiation	More	More
GO:0030198	extracellular matrix organization	More	More
GO:0030199	collagen fibril organization	More	More
GO:0030326	embryonic limb morphogenesis	More	More
GO:0030334	regulation of cell migration	More	More
GO:0030425	dendrite	More	More
GO:0030510	regulation of BMP signaling pathway	More	More
GO:0030534	adult behavior	More	More
GO:0030594	neurotransmitter receptor activity	More	More
GO:0030672	synaptic vesicle membrane	More	More
GO:0030799	regulation of cyclic nucleotide metabolic pro	More	More
GO:0030801	positive regulation of cyclic nucleotide meta	More	More
GO:0030802	regulation of cyclic nucleotide biosynthetic p	More	More
GO:0030804	positive regulation of cyclic nucleotide biosy	More	More
GO:0030808	regulation of nucleotide biosynthetic proces	More	More
GO:0030810	positive regulation of nucleotide biosyntheti	More	More
GO:0030814	regulation of cAMP metabolic process	More	More
GO:0030816	positive regulation of cAMP metabolic proce	More	More
GO:0030817	regulation of cAMP biosynthetic process	More	More

GO:0030819	positive regulation of cAMP biosynthetic pro	More	More
GO:0030855	epithelial cell differentiation	More	More
GO:0031012	extracellular matrix	More	More
GO:0031128	developmental induction	More	More
GO:0031279	regulation of cyclase activity	More	More
GO:0031344	regulation of cell projection organization	More	More
GO:0031362	anchored to external side of plasma membr	More	More
GO:0031644	regulation of neurological system process	More	More
GO:0032355	response to estradiol stimulus	More	More
GO:0032501	multicellular organismal process	More	More
GO:0032502	developmental process	More	More
GO:0032879	regulation of localization	More	More
GO:0033267	axon part	More	More
GO:0033555	multicellular organismal response to stress	More	More
GO:0033993	response to lipid	More	More
GO:0034220	ion transmembrane transport	More	More
GO:0034702	ion channel complex	More	More
GO:0034703	cation channel complex	More	More
GO:0034705	potassium channel complex	More	More
GO:0034765	regulation of ion transmembrane transport	More	More
GO:0035107	appendage morphogenesis	More	More
GO:0035108	limb morphogenesis	More	More
GO:0035113	embryonic appendage morphogenesis	More	More
GO:0035115	embryonic forelimb morphogenesis	More	More
GO:0035136	forelimb morphogenesis	More	More
GO:0035150	regulation of tube size	More	More
GO:0035239	tube morphogenesis	More	More
GO:0035725	sodium ion transmembrane transport	More	More
GO:0036342	post-anal tail morphogenesis	More	More
GO:0040011	locomotion	More	More
GO:0040012	regulation of locomotion	More	More
GO:0042127	regulation of cell proliferation	More	More
GO:0042383	sarcolemma	More	More
GO:0042391	regulation of membrane potential	More	More
GO:0042472	inner ear morphogenesis	More	More
GO:0042995	cell projection	More	More
GO:0043005	neuron projection	More	More
GO:0043025	neuronal cell body	More	More
GO:0043062	extracellular structure organization	More	More
GO:0043196	varicosity	More	More
GO:0043269	regulation of ion transport	More	More
GO:0043270	positive regulation of ion transport	More	More
GO:0043565	sequence-specific DNA binding	More	More
GO:0043627	response to estrogen stimulus	More	More
GO:0043679	axon terminus	More	More
GO:0044057	regulation of system process	More	More
GO:0044212	transcription regulatory region DNA binding	More	More
GO:0044297	cell body	More	More
GO:0044306	neuron projection terminus	More	More
GO:0044420	extracellular matrix part	More	More
GO:0044456	synapse part	More	More
GO:0044463	cell projection part	More	More
GO:0044699	single-organism process	More	More
GO:0044700	single organism signaling	More	More
GO:0044707	single-multicellular organism process	More	More
GO:0044708	single-organism behavior	More	More
GO:0044763	single-organism cellular process	More	More
GO:0044765	single-organism transport	More	More
GO:0044767	single-organism developmental process	More	More
GO:0045165	cell fate commitment	More	More
GO:0045168	cell-cell signaling involved in cell fate comm	More	More
GO:0045202	synapse	More	More
GO:0045211	postsynaptic membrane	More	More
GO:0045595	regulation of cell differentiation	More	More
GO:0045596	negative regulation of cell differentiation	More	More
GO:0045597	positive regulation of cell differentiation	More	More
GO:0045664	regulation of neuron differentiation	More	More
GO:0045666	positive regulation of neuron differentiation	More	More
GO:0045761	regulation of adenylate cyclase activity	More	More
GO:0045785	positive regulation of cell adhesion	More	More
GO:0045981	positive regulation of nucleotide metabolic p	More	More
GO:0045995	regulation of embryonic development	More	More
GO:0046058	cAMP metabolic process	More	More
GO:0046873	metal ion transmembrane transporter activit	More	More
GO:0046883	regulation of hormone secretion	More	More
GO:0046903	secretion	More	More
GO:0048167	regulation of synaptic plasticity	More	More
GO:0048468	cell development	More	More
GO:0048484	enteric nervous system development	More	More
GO:0048485	sympathetic nervous system development	More	More
GO:0048513	organ development	More	More
GO:0048514	blood vessel morphogenesis	More	More
GO:0048545	response to steroid hormone stimulus	More	More
GO:0048562	embryonic organ morphogenesis	More	More
GO:0048598	embryonic morphogenesis	More	More
GO:0048608	reproductive structure development	More	More
GO:0048634	regulation of muscle organ development	More	More
GO:0048646	anatomical structure formation involved in m	More	More
GO:0048663	neuron fate commitment	More	More
GO:0048665	neuron fate specification	More	More
GO:0048666	neuron development	More	More
GO:0048704	embryonic skeletal system morphogenesis	More	More
GO:0048705	skeletal system morphogenesis	More	More
GO:0048729	tissue morphogenesis	More	More
GO:0048731	system development	More	More
GO:0048732	gland development	More	More
GO:0048754	branching morphogenesis of an epithelial tu	More	More
GO:0048856	anatomical structure development	More	More
GO:0048869	cellular developmental process	More	More
GO:0048870	cell motility	More	More
GO:0050678	regulation of epithelial cell proliferation	More	More
GO:0050767	regulation of neurogenesis	More	More
GO:0050789	regulation of biological process	More	More

GO:0050793	regulation of developmental process	More	More
GO:0050794	regulation of cellular process	More	More
GO:0050796	regulation of insulin secretion	More	More
GO:0050804	regulation of synaptic transmission	More	More
GO:0050877	neurological system process	More	More
GO:0050880	regulation of blood vessel size	More	More
GO:0050890	cognition	More	More
GO:0051046	regulation of secretion	More	More
GO:0051049	regulation of transport	More	More
GO:0051093	negative regulation of developmental proce	More	More
GO:0051094	positive regulation of developmental proces	More	More
GO:0051216	cartilage development	More	More
GO:0051239	regulation of multicellular organismal proces	More	More
GO:0051270	regulation of cellular component movement	More	More
GO:0051339	regulation of lyase activity	More	More
GO:0051480	cytosolic calcium ion homeostasis	More	More
GO:0051588	regulation of neurotransmitter transport	More	More
GO:0051893	regulation of focal adhesion assembly	More	More
GO:0051899	membrane depolarization	More	More
GO:0051952	regulation of amine transport	More	More
GO:0051960	regulation of nervous system development	More	More
GO:0051969	regulation of transmission of nerve impulse	More	More
GO:0055024	regulation of cardiac muscle tissue develop	More	More
GO:0055074	calcium ion homeostasis	More	More
GO:0055085	transmembrane transport	More	More
GO:0060021	palate development	More	More
GO:0060065	uterus development	More	More
GO:0060284	regulation of cell development	More	More
GO:0060341	regulation of cellular localization	More	More
GO:0060536	cartilage morphogenesis	More	More
GO:0060688	regulation of morphogenesis of a branching	More	More
GO:0061061	muscle structure development	More	More
GO:0061138	morphogenesis of a branching epithelium	More	More
GO:0061448	connective tissue development	More	More
GO:0065007	biological regulation	More	More
GO:0070838	divalent metal ion transport	More	More
GO:0071495	cellular response to endogenous stimulus	More	More
GO:0071855	neuropeptide receptor binding	More	More
GO:0071868	cellular response to monoamine stimulus	More	More
GO:0071870	cellular response to catecholamine stimulus	More	More
GO:0072509	divalent inorganic cation transmembrane tra	More	More
GO:0072511	divalent inorganic cation transport	More	More
GO:0086010	membrane depolarization involved in regula	More	More
GO:0086012	membrane depolarization involved in regula	More	More
GO:0090087	regulation of peptide transport	More	More
GO:0090109	regulation of cell-substrate junction assemb	More	More
GO:0090257	regulation of muscle system process	More	More
GO:0090276	regulation of peptide hormone secretion	More	More
GO:0097060	synaptic membrane	More	More
GO:0097305	response to alcohol	More	More
GO:0097367	carbohydrate derivative binding	More	More
GO:0097458	neuron part	More	More
GO:0097485	neuron projection guidance	More	More
GO:1900371	regulation of purine nucleotide biosynthetic	More	More
GO:1900373	positive regulation of purine nucleotide bios	More	More
GO:1900542	regulation of purine nucleotide metabolic pr	More	More
GO:1900544	positive regulation of purine nucleotide meta	More	More
GO:1901681	sulfur compound binding	More	More
GO:1901861	regulation of muscle tissue development	More	More
GO:1901888	regulation of cell junction assembly	More	More
GO:2000026	regulation of multicellular organismal develo	More	More
GO:2000027	regulation of organ morphogenesis	More	More
GO:2000145	regulation of cell motility	More	More
KEGG_CALCIUM_SIGNALING_PATHWAY	Calcium signaling pathway	More	More
KEGG_DILATED_CARDIOMYOPATHY	Dilated cardiomyopathy	More	More
KEGG_ECM_RECEPTOR_INTERACTION	ECM-receptor interaction	More	More
KEGG_FOCAL_ADHESION	Focal adhesion	More	More
KEGG_REGULATION_OF_ACTIN_CYTOS	Regulation of actin cytoskeleton	More	More
KEGG_VASCULAR_SMOOTH_MUSCLE_C	Vascular smooth muscle contraction	More	More
KEGG_WNT_SIGNALING_PATHWAY	Wnt signaling pathway	More	More
PID_INTEGRIN1_PATHWAY	Beta1 integrin cell surface interactions	More	More
REACTOME_AMINE_LIGAND_BINDING_R	Genes involved in Amine ligand-binding rec	More	More
REACTOME_AMYLOIDS	Genes involved in Amyloids	More	More
REACTOME_AXON_GUIDANCE	Genes involved in Axon guidance	More	More
REACTOME_CELL_CELL_COMMUNICAT	Genes involved in Cell-Cell communication	More	More
REACTOME_CELL_JUNCTION_ORGANIZ	Genes involved in Cell junction organization	More	More
REACTOME_CLASS_B_2_SECRETIN_FA	Genes involved in Class B/2 (Secretin famil	More	More
REACTOME_COLLAGEN_FORMATION	Genes involved in Collagen formation	More	More
REACTOME_DEVELOPMENTAL_BIOLOG	Genes involved in Developmental Biology	More	More
REACTOME_EXTRACELLULAR_MATRIX	Genes involved in Extracellular matrix orga	More	More
REACTOME_G_ALPHA_S_SIGNALLING_	Genes involved in G alpha (s) signalling eve	More	More
REACTOME_INTEGRATION_OF_ENERGY	Genes involved in Integration of energy met	More	More
REACTOME_NCAM_SIGNALING_FOR_N	Genes involved in NCAM signaling for neur	More	More
REACTOME_NEUROTRANSMITTER_REC	Genes involved in Neurotransmitter Recept	More	More
REACTOME_OPIOID_SIGNALLING	Genes involved in Opioid Signalling	More	More
REACTOME_PHOSPHOLIPASE_C_MEDIA	Genes involved in Phospholipase C-mediat	More	More
REACTOME_POTASSIUM_CHANNELS	Genes involved in Potassium Channels	More	More
REACTOME_REGULATION_OF_INSULIN	Genes involved in Regulation of Insulin Sec	More	More
REACTOME_SIGNALLING_BY_NGF	Genes involved in Signalling by NGF	More	More
REACTOME_TRANSPORT_OF_GLUCOS	Genes involved in Transport of glucose and	More	More
REACTOME_TRANSPORT_OF_INORGAN	Genes involved in Transport of inorganic ca	More	More
REACTOME_VOLTAGE_GATED_POTASS	Genes involved in Voltage gated Potassium	More	More
WNT_SIGNALING	Genes related to Wnt-mediated signal trans	More	More
GO:0001819	positive regulation of cytokine production	Less	NA
GO:0002707	negative regulation of lymphocyte mediated	Less	NA
GO:0002710	negative regulation of T cell mediated immu	Less	NA
GO:0032490	detection of molecule of bacterial origin	Less	NA
GO:0042035	regulation of cytokine biosynthetic process	Less	NA
GO:0042108	positive regulation of cytokine biosynthetic p	Less	NA
GO:0045069	regulation of viral genome replication	Less	NA
GO:0048525	negative regulation of viral process	Less	NA
GO:0050706	regulation of interleukin-1 beta secretion	Less	NA
GO:0050718	positive regulation of interleukin-1 beta secr	Less	NA

KEGG_ALLOGRAFT_REJECTION	Allograft rejection	Less	NA
KEGG_CYTOSOLIC_DNA_SENSING_PAT	Cytosolic DNA-sensing pathway	Less	NA
KEGG_TYPE_I_DIABETES_MELLITUS	Type I diabetes mellitus	Less	NA
KEGG_VIRAL_MYOCARDITIS	Viral myocarditis	Less	NA
PID_IL12_2PATHWAY	IL12-mediated signaling events	Less	NA
PID_IL12_STAT4PATHWAY	IL12 signaling mediated by STAT4	Less	NA
PID_P53DOWNSTREAMPATHWAY	Direct p53 effectors	Less	NA
REACTOME_APOPTOSIS	Genes involved in Apoptosis	Less	NA
REACTOME_COSTIMULATION_BY_THE_	Genes involved in Costimulation by the CD	Less	NA
GO:0001502	cartilage condensation	More	NA
GO:0001945	lymph vessel development	More	NA
GO:0001952	regulation of cell-matrix adhesion	More	NA
GO:0003148	outflow tract septum morphogenesis	More	NA
GO:0004993	serotonin receptor activity	More	NA
GO:0005179	hormone activity	More	NA
GO:0005184	neuropeptide hormone activity	More	NA
GO:0005234	extracellular-glutamate-gated ion channel a	More	NA
GO:0005891	voltage-gated calcium channel complex	More	NA
GO:0006816	calcium ion transport	More	NA
GO:0006939	smooth muscle contraction	More	NA
GO:0007212	dopamine receptor signaling pathway	More	NA
GO:0007517	muscle organ development	More	NA
GO:0007605	sensory perception of sound	More	NA
GO:0008015	blood circulation	More	NA
GO:0008016	regulation of heart contraction	More	NA
GO:0008066	glutamate receptor activity	More	NA
GO:0009725	response to hormone stimulus	More	NA
GO:0010632	regulation of epithelial cell migration	More	NA
GO:0010721	negative regulation of cell development	More	NA
GO:0010976	positive regulation of neuron projection dev	More	NA
GO:0014821	phasic smooth muscle contraction	More	NA
GO:0016849	phosphorus-oxygen lyase activity	More	NA
GO:0021781	glial cell fate commitment	More	NA
GO:0022898	regulation of transmembrane transporter ac	More	NA
GO:0030072	peptide hormone secretion	More	NA
GO:0030336	negative regulation of cell migration	More	NA
GO:0030424	axon	More	NA
GO:0030878	thyroid gland development	More	NA
GO:0032409	regulation of transporter activity	More	NA
GO:0032412	regulation of ion transmembrane transporte	More	NA
GO:0032844	regulation of homeostatic process	More	NA
GO:0032989	cellular component morphogenesis	More	NA
GO:0034199	activation of protein kinase A activity	More	NA
GO:0034329	cell junction assembly	More	NA
GO:0034762	regulation of transmembrane transport	More	NA
GO:0040013	negative regulation of locomotion	More	NA
GO:0042493	response to drug	More	NA
GO:0042693	muscle cell fate commitment	More	NA
GO:0042886	amide transport	More	NA
GO:0043266	regulation of potassium ion transport	More	NA
GO:0043271	negative regulation of ion transport	More	NA
GO:0046879	hormone secretion	More	NA
GO:0048265	response to pain	More	NA
GO:0048588	developmental cell growth	More	NA
GO:0048806	genitalia development	More	NA
GO:0050433	regulation of catecholamine secretion	More	NA
GO:0050954	sensory perception of mechanical stimulus	More	NA
GO:0051271	negative regulation of cellular component m	More	NA
GO:0051924	regulation of calcium ion transport	More	NA
GO:0060023	soft palate development	More	NA
GO:0060037	pharyngeal system development	More	NA
GO:0060078	regulation of postsynaptic membrane poten	More	NA
GO:0060411	cardiac septum morphogenesis	More	NA
GO:0060420	regulation of heart growth	More	NA
GO:0070588	calcium ion transmembrane transport	More	NA
GO:0086001	regulation of cardiac muscle cell action pote	More	NA
GO:0086002	regulation of cardiac muscle cell action pote	More	NA
GO:0086009	membrane repolarization	More	NA
GO:0086011	membrane repolarization involved in regula	More	NA
GO:0086013	membrane repolarization involved in regula	More	NA
GO:0086014	regulation of atrial cardiac muscle cell actio	More	NA
GO:0086036	regulation of cardiac muscle cell membrane	More	NA
GO:0090092	regulation of transmembrane receptor prote	More	NA
GO:1901700	response to oxygen-containing compound	More	NA
GO:2000021	regulation of ion homeostasis	More	NA
GO:2000146	negative regulation of cell motility	More	NA
REACTOME_DOWNSTREAM_SIGNALING	Genes involved in Downstream signaling of	More	NA
REACTOME_PLATELET_HOMEOSTASIS	Genes involved in Platelet homeostasis	More	NA
REACTOME_SIGNALING_BY_FGFR	Genes involved in Signaling by FGFR	More	NA
REACTOME_SIGNALING_BY_FGFR_IN_	Genes involved in Signaling by FGFR in dis	More	NA

Significant PAM CpG	Cancer Type	Associated Gene
cg02706881	general kidney cancer	*C21orf123*
cg03562120	clear cell renal cell carcinoma	*WISP2*
cg04511534	both	*GGT6*
cg04598121	general kidney cancer	*PENK*
cg04988978	both	*MPO*
cg05379350	general kidney cancer	*GIT1*
cg06130787	general kidney cancer	*KLK10*
cg08749917	general kidney cancer	*RTP1*
cg10045881	clear cell renal cell carcinoma	*CHI3L2*
cg11098259	clear cell renal cell carcinoma	*AQP9*
cg12782180	general kidney cancer	*LEP*
cg12907644	general kidney cancer	*SAA2*
cg12939547	general kidney cancer	*VWA7*
cg13156411	general kidney cancer	*PTHR1*
cg14370448	clear cell renal cell carcinoma	*TBX6*
cg14391855	both	*RIN1*
cg14456683	general kidney cancer	*ZIC1*
cg15484375	both	*SAA1*
cg16592658	clear cell renal cell carcinoma	*EBI3*
cg17568996	general kidney cancer	*NFAM1*
cg18003231	general kidney cancer	*SLC25A18*
cg22628873	general kidney cancer	*GGT6*
cg22719623	general kidney cancer	*OPRM1*
cg23320056	general kidney cancer	*ARHGEF2*
cg26366091	clear cell renal cell carcinoma	*CHI3L2*
cg26514492	general kidney cancer	*GPR132*
cg26954174	clear cell renal cell carcinoma	*NOD2*
