# Supplementary figures and images for: DNA methylation profiling reveals novel diagnostic biomarkers in renal cell carcinoma
Source: BMC Med. 2014 Dec 4;12:235. doi: 10.1186/s12916-014-0235-x (PMC4265327; doi:10.1186/s12916-014-0235-x)

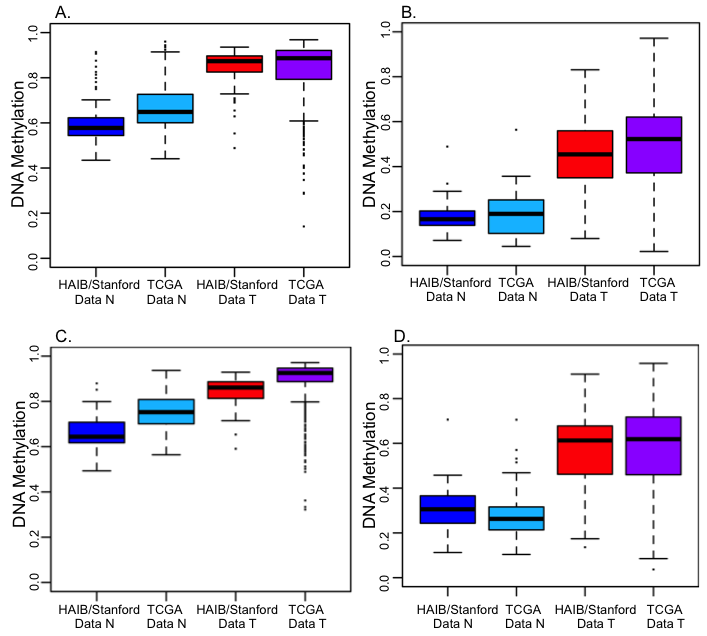

Supplement: Additional file 2: Figure S1. — Remaining diagnostic panel biomarker CpG values from models. DNA methylation of the five CpGs in both HudsonAlpha/Stanford patients and TCGA patients in the best RCC diagnostic model (cg13156411, cg14456683, cg18003231, cg12782180, and cg12782180; panel A–E), and the other three CpGs in both HudsonAlpha/Stanford patient and TCGA patients in the best ccRCC diagnostic model (cg11098259, cg14391855, and cg26366091; panel F–H). In all panels, HudsonAlpha/Stanford Data N versus HudsonAlpha/Stanford Data T and TCGA Data N versus TCGA Data T comparisons are significant (Mann-Whitney test; Bonferroni-adjusted P <0.0001). [file 12916_2014_235_MOESM2_ESM.zip › 1273016012136123_add1.tiff]

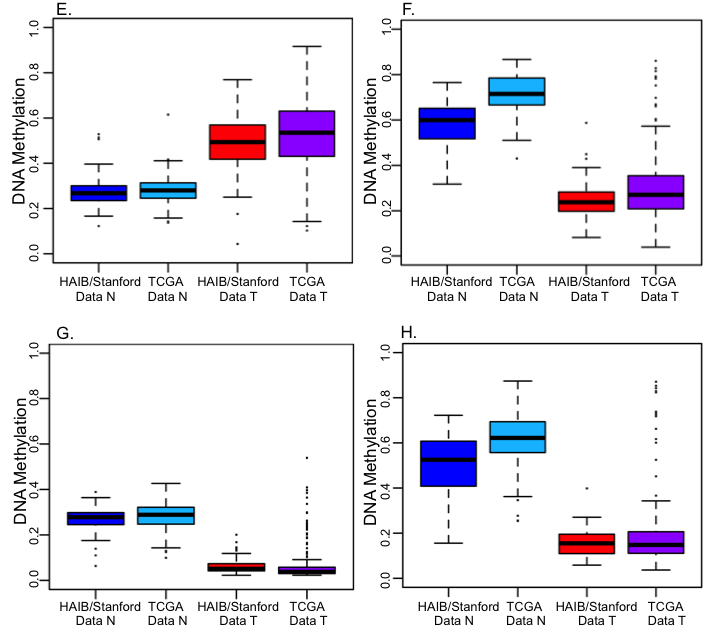

Supplement: Additional file 2: Figure S1. — Remaining diagnostic panel biomarker CpG values from models. DNA methylation of the five CpGs in both HudsonAlpha/Stanford patients and TCGA patients in the best RCC diagnostic model (cg13156411, cg14456683, cg18003231, cg12782180, and cg12782180; panel A–E), and the other three CpGs in both HudsonAlpha/Stanford patient and TCGA patients in the best ccRCC diagnostic model (cg11098259, cg14391855, and cg26366091; panel F–H). In all panels, HudsonAlpha/Stanford Data N versus HudsonAlpha/Stanford Data T and TCGA Data N versus TCGA Data T comparisons are significant (Mann-Whitney test; Bonferroni-adjusted P <0.0001). [file 12916_2014_235_MOESM2_ESM.zip › 1273016012136123_add3.tiff]

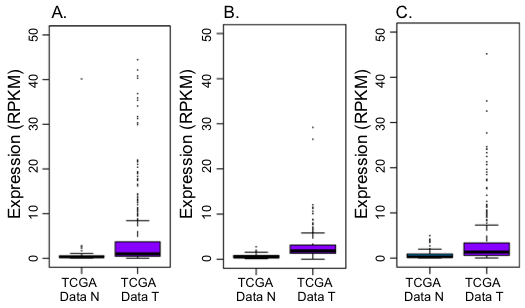

Supplement: Additional file 3: Figure S2. — RNA expression values for genes nearest other three predictive CpGs in ccRCC. RNA expression of the three other genes in TCGA patients in the best ccRCC diagnostic model (panel A–C, Mann-Whitney test; P <0.0001). The CpGs are all in the promoter regions of the respective genes (cg11098259 and AQP9; cg14391855 and RIN1; cg26366091 and CHI3L2; panel A–C). [file 12916_2014_235_MOESM3_ESM.tiff]
